# Molecular architecture and spatial organization of proteasomes in the human sperm nucleus

**DOI:** 10.1038/s41594-026-01870-z

**Published:** 2026-09-01

**Authors:** Piotr Kolata, Ália dos Santos, Oliver Knowles, Tom Dendooven, Matteo Allegretti

**Affiliations:** 1 Structural Studies Division, MRC Laboratory of Molecular Biology, Cambridge, UK; 2 Department of Bioengineering Sciences, Structural Biology Brussels, Vrije Universiteit Brussel, Brussels, Belgium; 3 VIB-VUB Center for Structural Biology, VIB, Brussels, Belgium

**Keywords:** Cryoelectron microscopy, Proteasome, Cryoelectron tomography, Developmental biology, Proteolysis

## Abstract

Proteasomes are fundamental for protein homeostasis and genome integrity and essential in spermatogenesis and fertilization. However, their presence, composition and role within the sperm nucleus are a subject of debate. Here we use in situ cryo-electron tomography in human sperm cells to elucidate the molecular architecture of nuclear proteasomes, which cluster in DNA-free, nuclear cavities within the sperm nucleus. We show that the main population of proteasomes consists of 20S core particles, with a smaller fraction of 20S capped by PA200 activator. Using single-particle cryo-electron microscopy of purified native human sperm proteasomes, we elucidate the features of the essential testis-specific subunit α4s, reporting the presence of a unique splice variant. We resolve a native peptide in the catalytic β2 subunit, providing insight into the proteolysis mechanism and PA200-mediated enhancement of trypsin activity. We show nuclear enrichment of proteasomes during sperm-cell differentiation in human testis tissue, with 20S and PA200 clustering following meiosis, at the spermatid stage. Our findings shed light on the organization and compositional diversity of nuclear proteasomes in human sperm cells, as well as their catalytic function.

## Abstract (web summary)

Proteasomes are essential for male fertility. Kolata, dos Santos, Dendooven and colleagues show large nuclear proteasomal assemblies segregated from DNA in human sperm, forming after meiosis and with a unique α4s isoform. A native substrate is bound at the 20S catalytic site.

## Main

The proteasome is an essential multicatalytic protease complex consisting of a barrel-shaped 20S core particle (CP) of ~750 kDa, capped by regulatory particles. In eukaryotes, the 20S core comprises 28 subunits arranged in four stacked rings (α1–7, β1–7)_2_, forming a hollow cylinder^1^. The outermost rings of α-subunits create two gated pores that control substrate entry, while the inner β-rings harbor the protease active sites. To degrade proteins, the 20S CP associates with proteasome activators that recognize substrates and open the pore to enable their entry. The main activator, the 19S regulatory particle, associates with the 20S to form a 26S holoenzyme. In addition to opening the gate, the 19S activator unfolds ubiquitylated proteins in an ATP-dependent manner and threads them into the 20S CP for proteolysis^2^.

The proteasome has critical roles in both cytoplasmic and nuclear protein quality control^3,4^. In the nucleus, proteasomes are crucial for degrading cell-cycle regulators, clearing misfolded proteins and dismantling protein complexes at DNA repair foci^3,5^. The nuclear proteasome pool is dynamically regulated. Under acute cellular stress, such as heat shock, oxidative or osmotic stress, nutrient starvation or inhibition of proteasome activity or nuclear export, proteasomes can reorganize into intranuclear, membraneless condensates through ubiquitin-mediated liquid–liquid phase separation^4,6–8^.

In the context of reproduction, a growing body of evidence indicates that spermatozoa harbor enzymatically active proteasomes that are essential both during spermatogenesis and after fertilization^9^. Consistent with this, inhibition of proteasome function leads to a dose-dependent reduction in motility^10^ and disrupts sperm-cell capacitation^11–14^. Proteasomes have been detected in multiple compartments of the sperm cell, including the acrosome, periacrosomal surface, plasma membrane, perinuclear theca^13,15–19^ and the connecting piece and tail^18,20^. Studies across different mammalian model organisms propose a role for sperm proteasomes in the degradation of glycoproteins in the egg’s zona pellucida and show that inhibition of proteasome activity blocks sperm penetration, thereby preventing fertilization^15,16,20,21^.

While the cytosolic, extracellular and membrane-associated functions of sperm proteasomes have been well studied^9,16^, less is known about the role of nuclear proteasomes in mammalian sperm. Proteasomes are essential for nuclear DNA compaction during spermatogenesis, where the histones in the chromatin of postmeiotic germ cells are replaced with protamines. The timely removal of histones is facilitated by a testis-specific proteasome variant, in which the canonical α4 (PMSA7) proteasome subunit is replaced by the α4s (PSMA8) subunit in spermatocytes^22–24^. α4s appears to be essential for effective meiotic progression, being localized to and potentially involved in disassembly of the synaptonemal complex^22,24,25^. In spermatid nuclei, the PA200 activator engages the testis-specific proteasome, allowing acetylation-dependent proteolysis of core histones^26–29^. Moreover, both PA200 and α4s are thought to be essential for sperm differentiation and maturation. In mice lacking PA200, the removal of core histones from elongating spermatids is significantly delayed, while deletion of the α4s subunit leads not only to defective histone removal but also to meiotic arrest and male infertility^9,23–25,30^. How these nuclear proteasomes are organized in the dense sperm head remains unknown but proteasome components were found to colocalize to electron-bright regions of the sperm nucleus (‘lacunae’) using immunogold labeling and transmission electron microscopy (EM)^31^. The size of these nuclear lacunae has been proposed to correlate with levels of chromatin condensation, sperm motility and fertility outcomes^32,33^.

Here, we used in situ cryo-electron tomography (cryo-ET) and subtomogram averaging to reveal large proteasome populations within nuclear lacunae of mature and motile human sperm cells. The lacunae were up to 1 µm in length and were enriched in 20S proteasomes, with an additional fraction of PA200-capped proteasomes. Using immunohistochemistry in human testis tissue, we show that proteasome-rich lacunae develop in postmeiotic cells at the spermatid stage. In addition, our high-resolution structures of native human sperm proteasomes revealed a unique splice variant of the testis-specific subunit α4s and an unexpected density in the β2 subunit active site. Collectively, our work maps and characterizes proteasome populations in the sperm nucleus, shedding light on substrate engagement and PA200-driven proteasome activation mechanisms.

## Results

### Nuclear lacunae in human sperm cells contain three populations of proteasome complexes

**Fig. 1:**
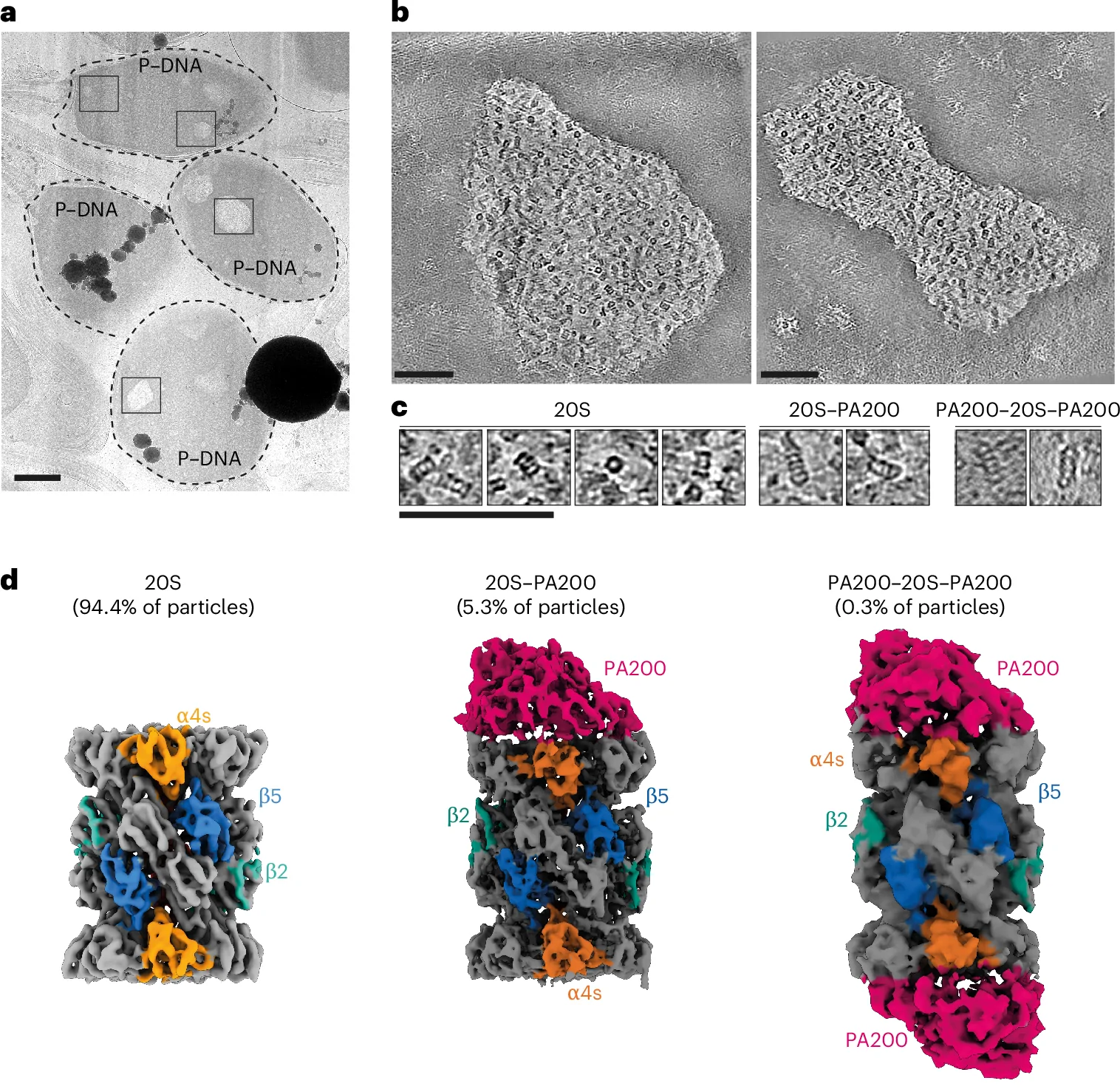
Nuclear lacunae in human sperm cells are proteasomal compartments. **a**, Cryo-EM low-magnification lamellae map showing four human sperm heads. Rectangles indicate regions with sperm lacunae targeted for tilt-series acquisition. The image is representative of >8 independent experiments, using samples from >10 donors. Scale bar, 1 μm. P–DNA, protamine–DNA. **b**, *z* slices from reconstructed tomograms showing barrel-shaped protein complexes inside membraneless nuclear compartments. Scale bars, 100 nm. **c**, Zoomed-in regions from tomograms showing barrel-shaped particles corresponding to 20S, 20S–PA200 and PA200–20S–PA200 inside lacunae. Scale bar, 100 nm. **d**, Subtomogram averaging classes showing three populations: 20S CP (43,128 particles, 94.4% of total particles), 20S–PA200 complexes (2,430 particles, 5.3% of total particles) and PA200–20S–PA200 (245 particles, 0.3% of total particles).

**Extended Data Fig. 1:**
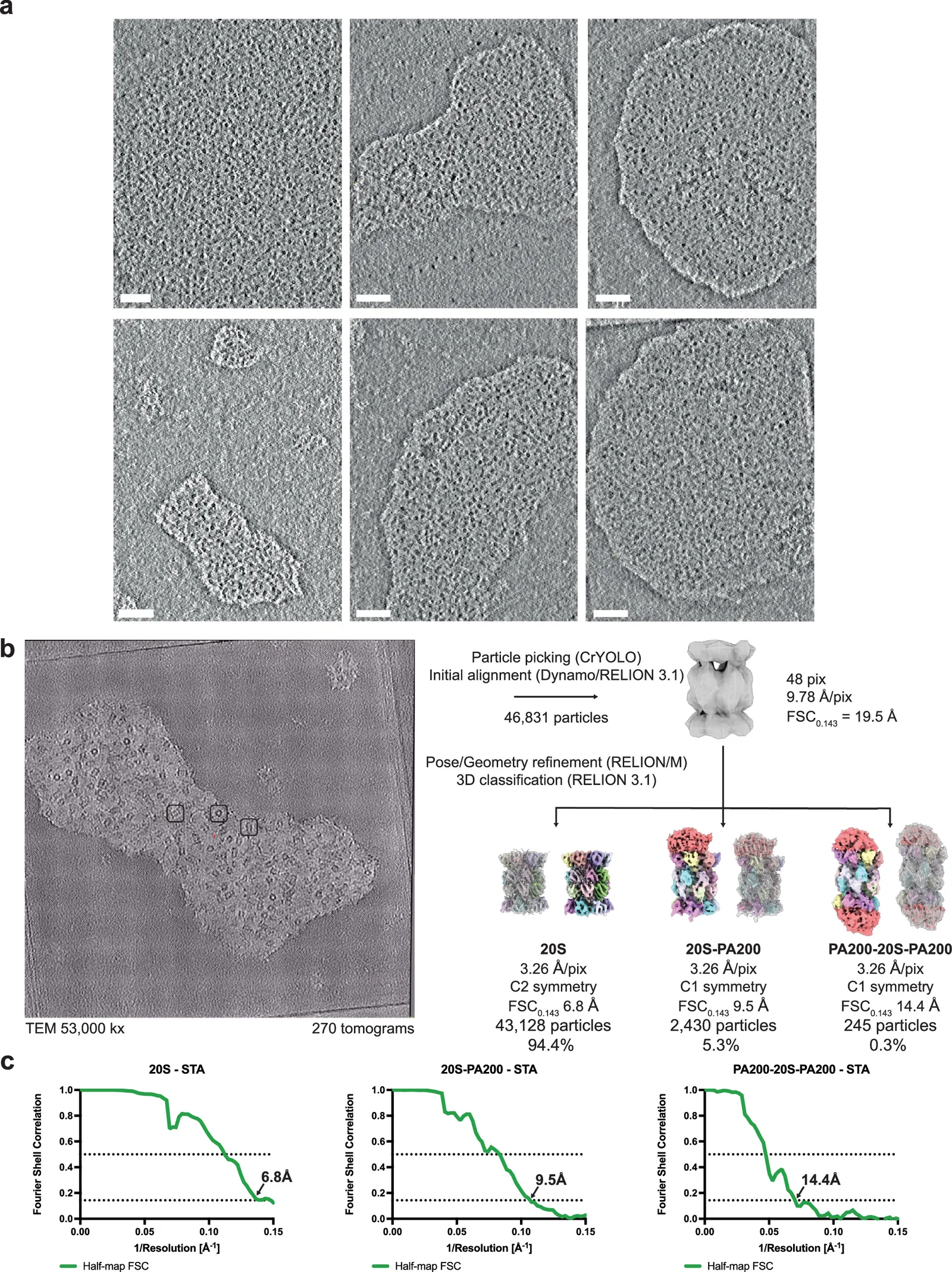
Gallery of lacunae and subtomogram averaging processing pipeline of nuclear sperm proteasomes. **a**, cryo-tomographic slices from deconvolved tomograms of six nuclear lacunae filled with barrel-shaped proteins. Scale bar = 100 nm. **b**, Details of the subtomogram averaging processing pipeline of 20S, 20S-PA200 and PA200-20S-PA200 proteasomes. Subtomogram averages with fitted PDBs are shown. **c**, Gold-standard Fourier Shell Correlation (FSC) curves for the Subtomogram Averaging (STA) maps: 20S, 20S-PA200 and PA200-20S-PA200. The resolution values at FSC = 0.143 (half-map FSC) are indicated. The 20S resolution is limited by Nyquist frequency. Source data

**Extended Data Fig. 2:**
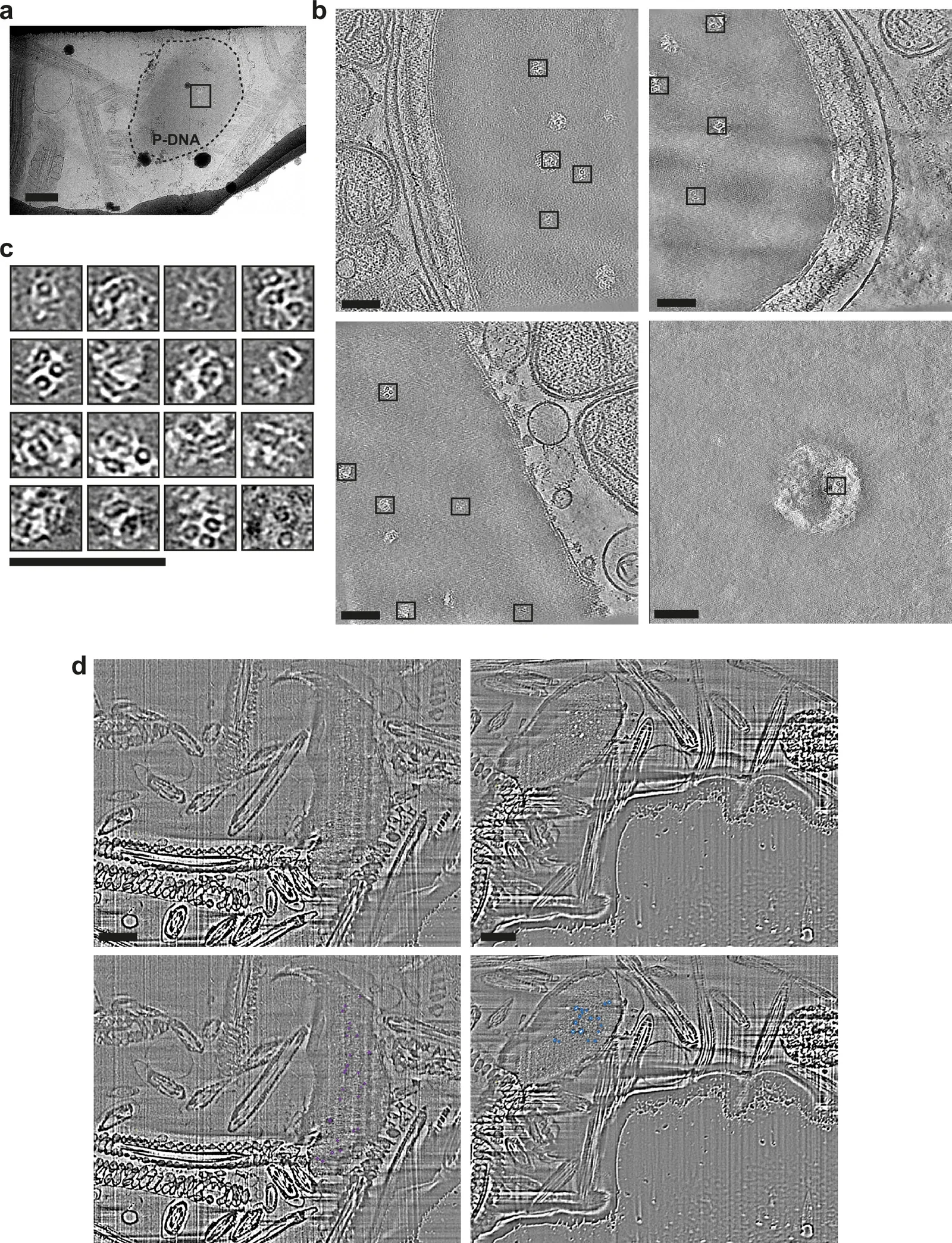
Proteasome complexes in mouse sperm nuclear *lacunae*. **a**, cryo-TEM low magnification lamellae map showing one mouse sperm head (dashed line). The square indicates a region with sperm *lacunae* targeted for tilt-series acquisition. Scale bar = 1 *μ*m. Image is representative of data collected >3 independent experiments and 2 biological replicates. **b**, z-slices from reconstructed tomograms showing barrel-shaped protein complexes inside membraneless nuclear compartments. Scale bar = 100 nm. **c**, zoom-in regions from tomograms shown in **(**b) with barrel-shaped proteins inside *lacunae*. Scale bar = 100 nm. **d**, representative z-slices of 3D reconstructed cryo-SEM volumetric data of mouse sperm cells. Small sperm *lacunae* are visible within the nucleus. Scale bar = 1 *μ*m. Bottom panels show detail of segmented *lacunae* in colour (purple and blue).

To investigate the localization and ultrastructure of nuclear proteasomes in human sperm cells, we used cryo-ET. Human sperm cells were subjected to cryo-focused ion beam (cryo-FIB) milling to generate thin lamellae. In the lamellae maps, we found electron-bright regions in the sperm nuclei, similar to the lacunae described by Haraguchi et al.^31^ (Fig. 1a). Reconstructed tomograms of these regions revealed that lacunae were segregated from the condensed protamine–DNA, as membraneless compartments, and enriched in barrel-shaped protein complexes (Fig. 1b and Extended Data Fig. 1a). Subtomogram averaging and three-dimensional (3D) classification of these complexes revealed three different proteasome populations: 20S proteasome CP (94.4% of assigned particles, 6.8-Å resolution), 20S capped by a single PA200 activator (20S–PA200, 5.3% particles, 9.5-Å resolution) and 20S capped by two PA200 activators (PA200–20S–PA200, 0.3% particles, 14.4-Å resolution) (Fig. 1c,d, Extended Data Fig. 1b,c, Supplementary Video 1 and Table 1). Next, we investigated whether nuclear proteasome lacunae were a conserved feature between species. For this, we performed cryo-ET on lamellae of mouse sperm cells (Extended Data Fig. 2a,b). Similar to human sperm cells, we found proteasome complexes in the nuclear lacunae of mouse sperm cells (Extended Data Fig. 2b,c). However, the size of mouse proteasomal lacunae was significantly smaller compared to human (Extended Data Figs. 1 and 2a,b).

**Table 1:** Cryo-ET data collection, data processing and refinement statistics

	(1) 20S (EMD-55396)	(2) 20S–PA200 (EMD-55397)	(3) PA200–20S–PA200 (EMD-55398)
**Data collection and processing**			
Microscope	TFS Krios G3i/G4	TFS Krios G3i/G4	TFS Krios G3i/G4
Magnification	53,000	53,000	53,000
Voltage (kV)	300	300	300
Electron fluence (e^−^ per Å^2^)	100	100	100
Detector	K3/Falcon4i	K3/Falcon4i	K3/Falcon4i
Energy filter	Bioquantum/SelectrisX	Bioquantum/SelectrisX	Bioquantum/SelectrisX
Defocus range (μm)	−2 to −5	−2 to −5	−2 to −5
Pixel size (Å)	1.63	1.63	1.63
Symmetry imposed	*C*_2_	*C*_1_	*C*_2_
Initial particle images (no.)	46,831		
Final particle images (no.)	43,128	2,430	245
Map resolution (Å)	6.8	9.5	14.4
FSC threshold	0.143	0.143	0.143
Map sharpening *B* factor (Å^2^)	−180	−180	−180

**Fig. 2:**
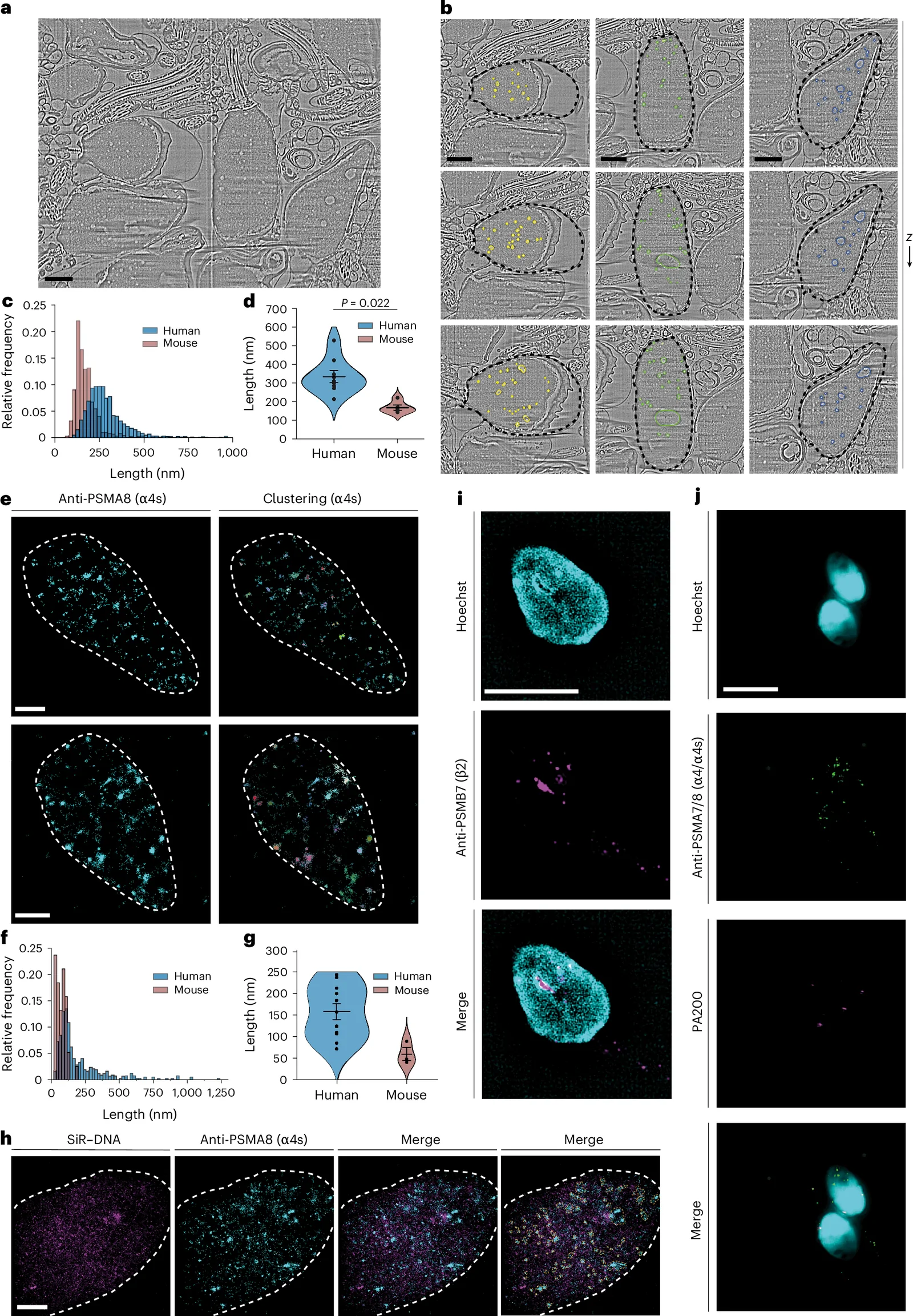
Human sperm nuclear lacunae are variable in size and positive for proteasomes with testis-specific subunit PSMA8, which segregates from DNA. **a**, A *z* slice of 3D reconstructed cryo-SEM volumetric data of human sperm cells. Sperm lacunae are visible in the nucleus. Scale bar, 1 μm. **b**, Detail of three human sperm heads from volumetric data shown in **a**. Segmented lacunae are shown in color (yellow, green and blue) at three different *z* heights. The sperm nucleus is shown in dashed black lines. Scale bars, 1 μm. **c**, Relative frequency plot of the Feret diameters of segmented human sperm lacunae (blue) and comparison to values from mouse data (red; Extended Data Fig. 2). Feret diameter was defined as the longest *xy* distance of a segmented lacuna. The histogram depicts the values for 2,147 lacunae segmented from nine human sperm cells and 866 lacunae segmented from five mouse sperm cells. The mean Feret diameter ± s.e.m. was 326 ± 6 nm for human lacunae and 173.3 ± 2.1 nm for mouse lacunae. **d**, Violin plots for data shown in **c**. Each data point represents the average lacunae diameter of an individual cell. The mean diameter ± s.e.m. is plotted for human (blue; 335.3 ± 30.5 nm, *n* = 9 cells) and mouse (red; 169.4 ± 13.6 nm, *n* = 5 cells) cells. Statistical analysis was conducted using a two-sided *t*-test (*P* = 0.0022). **e**, STORM of sperm-specific proteasome subunit α4s (PSMA8) in human sperm cells. The white dashed line shows the sperm nuclear region. Right: α4s cluster identification following DBSCAN analysis of α4s within nuclear region. Each cluster is shown in a different color. Scale bars, 1 μm (*n* = 11 cells from three independent experiments). **f**, Histogram of α4s clusters identified using STORM in human (blue) and mouse (red) cells (Extended Data Fig. 3a). The histogram represents values from 415 α4s clusters obtained from 11 human sperm cells and 37 α4s clusters from three mouse sperm cells. The mean Feret diameter ± s.e.m. was 199.0 ± 7.2 nm for human proteasome clusters and 72.0 ± 5.9 nm for mouse proteasome clusters. **g**, Violin plots for data shown in **f**, where data points represent the average Feret diameter of α4s clusters in each cell. The mean diameter ± s.e.m. is shown for human (blue; 157.3 ± 18.4 nm, *n* = 11 cells) and mouse (red; 59.7 ± 25.7 nm, *n* = 3 cells) cells. For mouse data, >10 cells were measured by STORM but clusters were only identified in three cells. Clusters were identified in all measured human cells. **h**, STORM imaging of DNA and α4s in a human sperm cell, using SiR–DNA and immunofluorescence staining against α4s. Regions of α4s density segregated from DNA are shown within dashed yellow lines in the rightmost panel. Scale bar, 1 μm. The image is representative of ten cells from three independent experiments. **i**, SIM showing proteasome subunit PSMB7 (β2) forming foci in the nucleus of sperm cells (magenta) in regions of no DNA staining (Hoechst). Scale bar, 5 μm. The image is representative of >50 cells from >3 biological replicates. **j**, SIM imaging of human sperm cells showing nuclear foci of α4/α4s (PSMA7/8) proteasome subunit (green) and partial colocalization with PA200 (magenta). Small α4/α4s foci were also present in the tail. Scale bar, 5 μm. The image is representative of >50 cells from >3 biological replicates. Source data

Lamellae prepared for cryo-ET (thickness ≈ 120–220 nm) only represent 5% of the total volume of a sperm cell. To assess the size and variability of proteasome-rich lacunae, we performed volumetric imaging of entire human sperm cells using cryo-FIB/SEM (scanning EM) (Fig. 2a and Supplementary Video 2)^34^. The reconstructed volumes revealed that nuclear lacunae (Fig. 2a,b) have an average Feret diameter (defined as the longest linear distance of a segmented lacuna) of 326 ± 6 nm (Fig. 2c,d). Among all segmented human sperm lacunae, 7% exceeded a Feret diameter of 500 nm and 2.3% were larger than 1 μm, consistent with our cryo-ET observations (Figs. 1a and 2c and Extended Data Fig. 1a). Similarly, segmented cryo-FIB/SEM volumes of mouse sperm cells showed a marked reduction in the average Feret diameter of lacunae to 173 ± 2 nm, with only 0.2% larger than 500 nm, none exceeding 600 nm and 42% smaller than 150 nm (Fig. 2c,d and Extended Data Fig. 2d).

**Extended Data Fig. 3:**
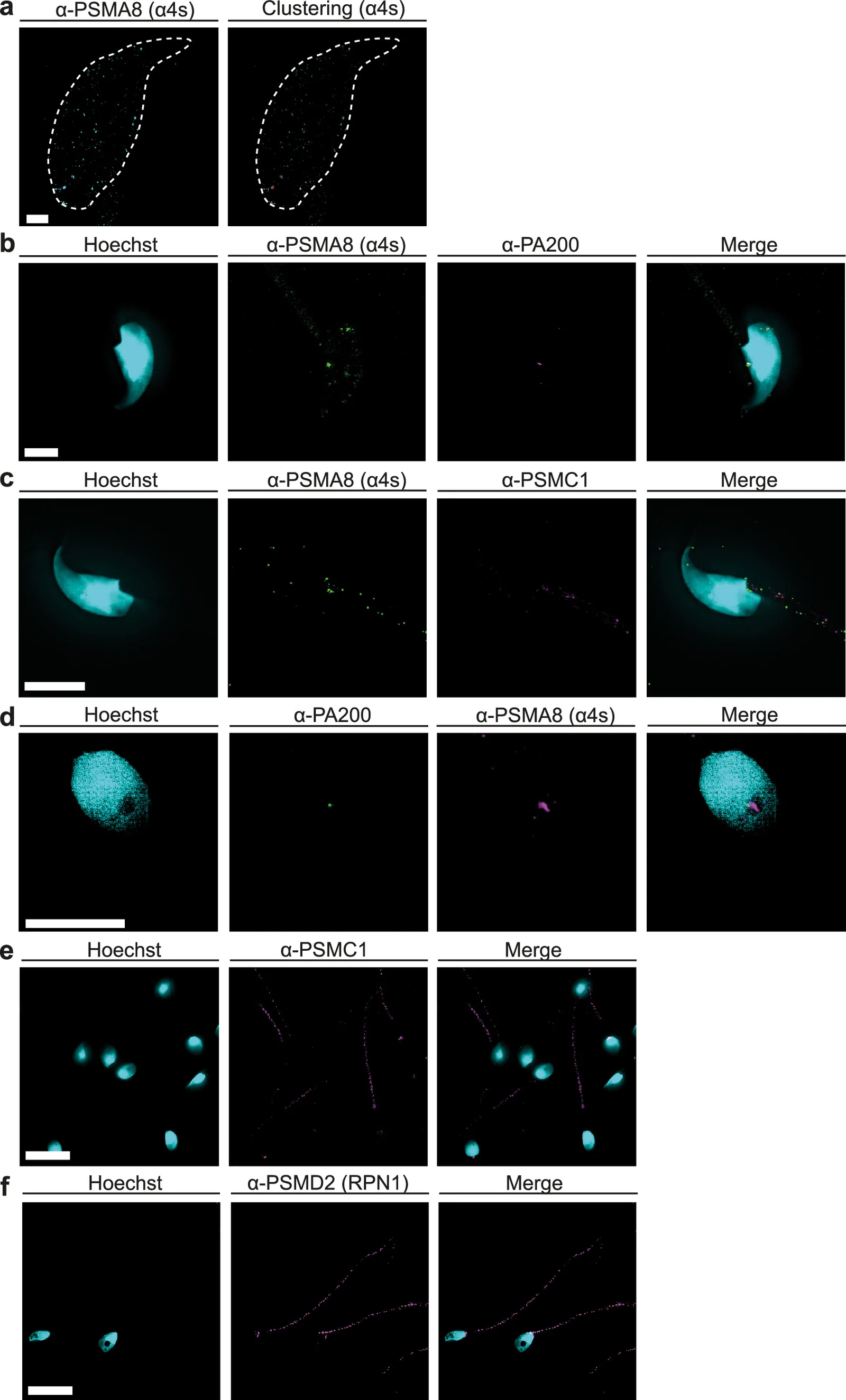
Proteasome distribution and clustering in mouse and human sperm cells. **a**, STORM of sperm-specific proteasome subunit PSMA8 (*α*4 s) in a mouse sperm cell. The white dashed line shows the sperm nuclear region. The right panel shows *α*4 s cluster identification in red, superimposed to non-clustered localisations (cyan/grey) following DBSCAN analysis. Scale bar = 1 *μ*m. Image is representative of data collected for >10 cells (3 independent experiments). **b**, SIM image of mouse sperm cell showing foci of *α*4 s (green) and partial co-localisation with less abundant PA200 (magenta). Scale bar = 5 *μ*m. Image is representative of > 20 cells, 3 biological replicates. **c**, SIM image of mouse sperm cell showing localisation of *α*4 s and 19S subunit PSMC1. Scale bar = 5 *μ*m. Image is representative of > 20 cells, 3 biological replicates. **d**, SIM image showing localisation of *α*4 s in the nucleus of human sperm cells, segregated from DNA signal (Hoechst, cyan). PA200 signal can be observed partially localising to the same region of the nucleus. Scale bar = 5 *μ*m. Image is representative of >50 cells (>3 biological replicates). **e**, SIM image showing localisation of the AAA-ATPase subunit of 19S, PSMC1 (magenta) in the tail of human sperm cells. Scale bar = 10 *μ*m. Image is representative of >20 cells (2 biological replicates). **f**, SIM image showing localisation of the 19S subunit PSMD2 (Rpn1) (magenta) in the tail of human sperm cells. Scale bar = 10 *μ*m. Image is representative of >20 cells (2 biological replicates).

To corroborate these results with an orthogonal method and measure the size distribution of nuclear proteasome clusters (rather than lacunae), we used stochastic optical reconstruction microscopy (STORM), followed by cluster analysis with density-based spatial clustering of applications with noise (DBSCAN)^35^. The sperm-specific 20S subunit PSMA8 (α4s) formed clusters within the nucleus of human sperm cells (Fig. 2e), displaying cluster size distributions comparable to those obtained with cryo-SEM volumetric data, albeit with smaller predicted dimensions (Fig. 2f,g). Human sperm α4s clusters had an average Feret diameter of 199 ± 7.2 nm, with 7.2% exceeding 500 nm and 0.7% larger than 1 μm. Furthermore, proteasome clusters did not colocalize with DNA staining, consistent with our cryo-ET observation that lacunae are segregated from DNA (Fig. 2h). Mouse sperm STORM data were also in line with cryo-SEM segmentation data, showing fewer clusters than in human sperm and a marked reduction in cluster size, with an average Feret diameter of 72 ± 5.9 nm, 97% smaller than 150 nm and none exceeding 200 nm in diameter (Fig. 2f,g and Extended Data Fig. 3a). In mouse sperm cells, PSMA8 (α4s) localizations did not always form regions of sufficient density to constitute clusters, measured by DBSCAN. Immunofluorescence staining of human sperm cells, followed by structured illumination microscopy (SIM), confirmed the presence of proteasome foci localized to regions largely devoid of DNA (Fig. 2i,j and Extended Data Fig. 3d), in agreement with STORM (Fig. 2h). A small subset of 20S foci colocalized with PA200 (Fig. 2j), consistent with the low fraction of capped proteasomes identified by subtomogram averaging (Fig. 1d). SIM imaging of mouse sperm cells revealed a similar distribution of 20S and PA200 (Extended Data Fig. 3b).

Interestingly, we did not detect nuclear signal for the 19S regulatory particle in ejaculated human sperm, using AAA-ATPase PSMC1 and PSMD2 (Rpn1) as markers. Instead, both proteins were enriched in the tail region (Extended Data Fig. 3e,f). In mouse sperm, the PSMC1 signal was substantially weaker overall and was also absent from the nucleus (Extended Data Fig. 3c).

Together, our integrative imaging data, spanning cryo-ET, cryo-SEM, STORM and SIM imaging, demonstrate three populations of proteasome complexes clustering within nuclear lacunae of human spermatozoa: uncapped 20S CP, 20S–PA200 and PA200–20S–PA200, with no detectable quantities of 26S complexes. Human nuclear lacunae were also larger on average than those of mouse sperm, emphasizing species-specific differences in gamete nuclear organization.

### Single-particle cryo-EM reveals unique features of proteasomes isolated from human sperm cells

**Fig. 3:**
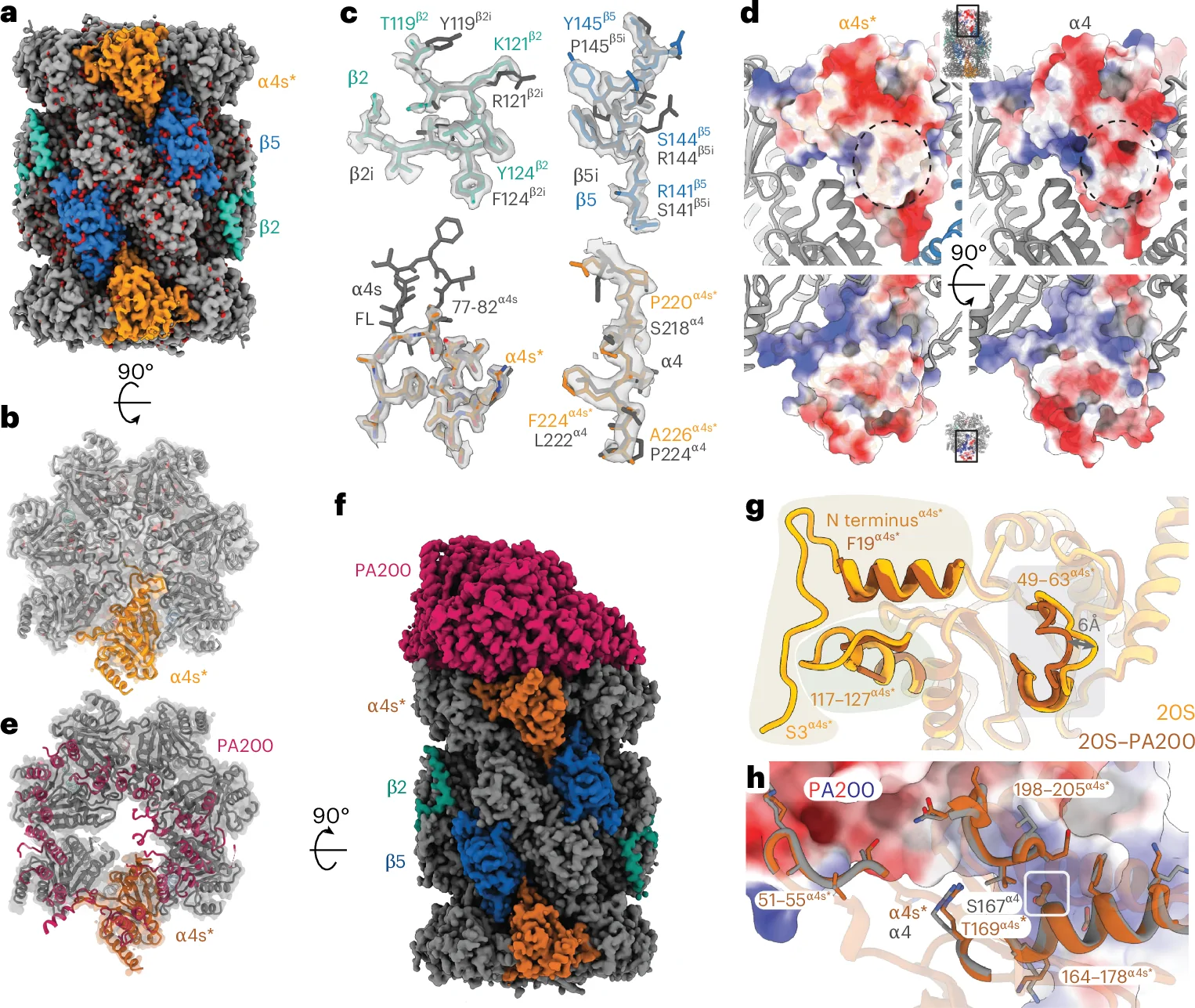
Single-particle cryo-EM structures of proteasomes isolated from human sperm cells. **a**, Segmented map of human sperm 20S CP at 1.85-Å resolution. Key subunits are indicated: α4s* (orange), β5 (blue) and β2 (cyan). Water molecules are shown in red. **b**, Constricted pore of the sperm 20S CP. The segmented map is shown as a transparent surface and the corresponding structure is shown in a cartoon representation. The map and structure are colored according to **a**. **c**, The canonical β2 and β5 subunits are present in the sperm proteasome and an alternatively spliced α4s variant, referred to as α4s*, replaces the α4 subunit. Residues 119–127^β2/β2i^, 141–149^β5/β5i^, 217–224^α4s*^/215–222^α4^ and 74–86^α4s*^/74–92^α4s FL^ are shown as sticks and colored as in **a**. The densities corresponding to these residue stretches in the sperm 20S CP are shown as a transparent surface. β2, β5 and α4s coordinates from sperm 20S CP are shown. β2i and β5i immunoproteasome coordinates (PDB 7B12)^76^ were rigid-body fitted into the sperm 20S map for comparison. The canonical α4s (UniProt Q8TAA3-1) is referred to as α4s FL. The alternatively spliced variant lacking the α4s-specific loop 77–82^α4s FL^ (UniProt Q8TAA3-5) is referred to as α4s*. α4s FL coordinates were modeled as described in Methods. Key residues that allowed subunit identification are indicated. **d**, Most substitutions in α4s occur at the solvent-exposed surface. α4s* (left) and α4 (right; PDB 5LE5) coordinates are shown as an electrostatic surface, while other proteasomal subunits are shown as cartoons. The calculated mean Coulombic electrostatic potential is −0.09 kcal mol^−1^ per e^−^ for α4 and 0.14 kcal mol^−1^ per e^−^ for α4s*. The substitutions D39^α4^→N41^α4s^, T141^α4^→I143^α4s^, E182^α4^→A184^α4s^, D185^α4^→S187^α4s^ and K227^α4^→L229^α4s^ create an extensive, uncharged patch on the α4s* surface, shown as a dashed circle. The side view (top) and top view (bottom) are shown. The regions of the structure shown are indicated in the insets by black rectangles. **e**,**f**, The structure of sperm 20S–PA200 proteasome at 2.9-Å resolution. In **e**, the opened pore of the 20S–PA200 proteasome is shown. The segmented map is shown as a transparent surface and the corresponding structure is shown as cartoons. α4s* is indicated and colored in dark orange. For PA200, only structural features interacting with the α-subunits are shown. In **f**, the segmented map is shown with key subunits indicated: PA200 (dark pink), α4s* (dark orange), β5 (dark blue) and β2 (dark cyan). **g**, Conformational changes in α4s* upon binding of PA200. α4s* from the sperm 20S CP is shown as orange cartoons, while that from 20S–PA200 is in dark orange. The disordered N-terminal (residues 3–18^α4s*^) and 117–127^α4s*^ loops of 20S–PA200, as well as the rearranged 49–63^α4s*^ loop, are indicated and highlighted over the regions that do not undergo conformational changes, shown as transparent cartoons. **h**, Upon docking, α4s* interacts with PA200 through mostly conserved interface formed by loops 51–55^α4s*^ and 198–205^α4s*^, as well as with the 164–178^α4s*^ helix. α4 (gray) and α4s* (dark orange) structural features interacting with PA200 are indicated and shown as cartoons and sticks. PA200 of sperm 20S–PA200 is shown as an electrostatic surface. The substitution S167^α4^→T169^α4s^ is indicated, with the T169^α4s^ side chain shown in ball-and-stick representation.

**Extended Data Fig. 4:**
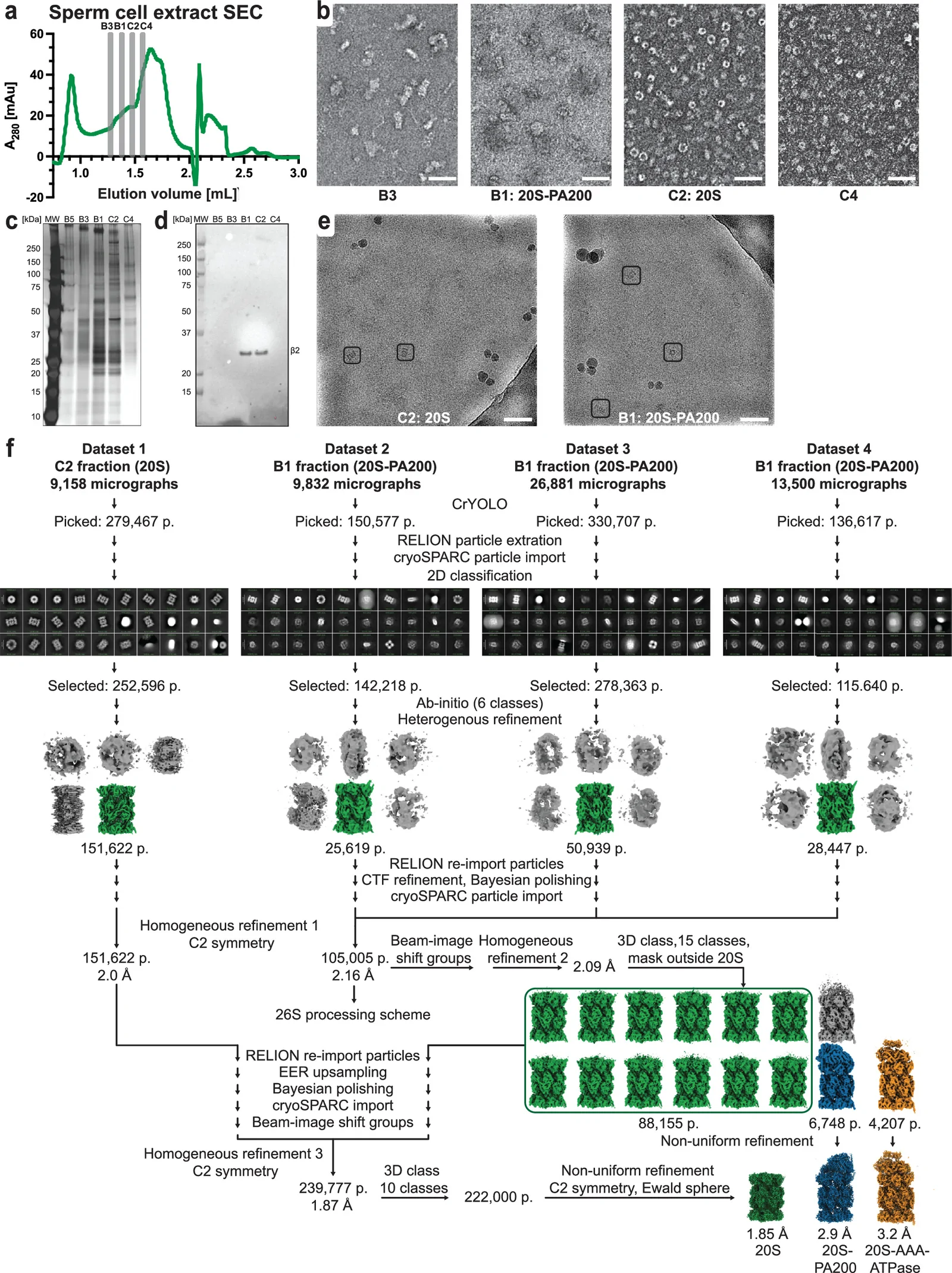
Purification and single-particle cryo-EM data processing of human sperm proteasomes. **a**, Size exclusion chromatography (SEC) of the sperm cell extract. The fractions are indicated. **b**, negative−stain transmission electron microscopy micrographs corresponding to SEC fractions from (**a**). Scale bar = 50 nm **c**, silver-stained SDS-PAGE of SEC fractions from (**a**)**. d**, immunoblotting against PSMB7 (*β*2) subunit of 20S confirmed proteasome elution in B1 and C2 fractions. The same fractions were loaded as in (**c**). **e**, cryo-EM micrographs of the C2 (left) and B1 (right) fractions. Protein particles are marked with a black box. Scale bar = 50 nm. Representative data from two independent experiments are shown in the **a-e** panels. **f**, single−particle cryo-EM data processing pipeline. “p.” - particle number at corresponding processing step. 2D class averages of the initial particle selection, as well as maps from key processing steps, are shown. “26S processing scheme” corresponds to the scheme in Extended Data Fig. 8a. Source data

**Extended Data Fig. 5:**
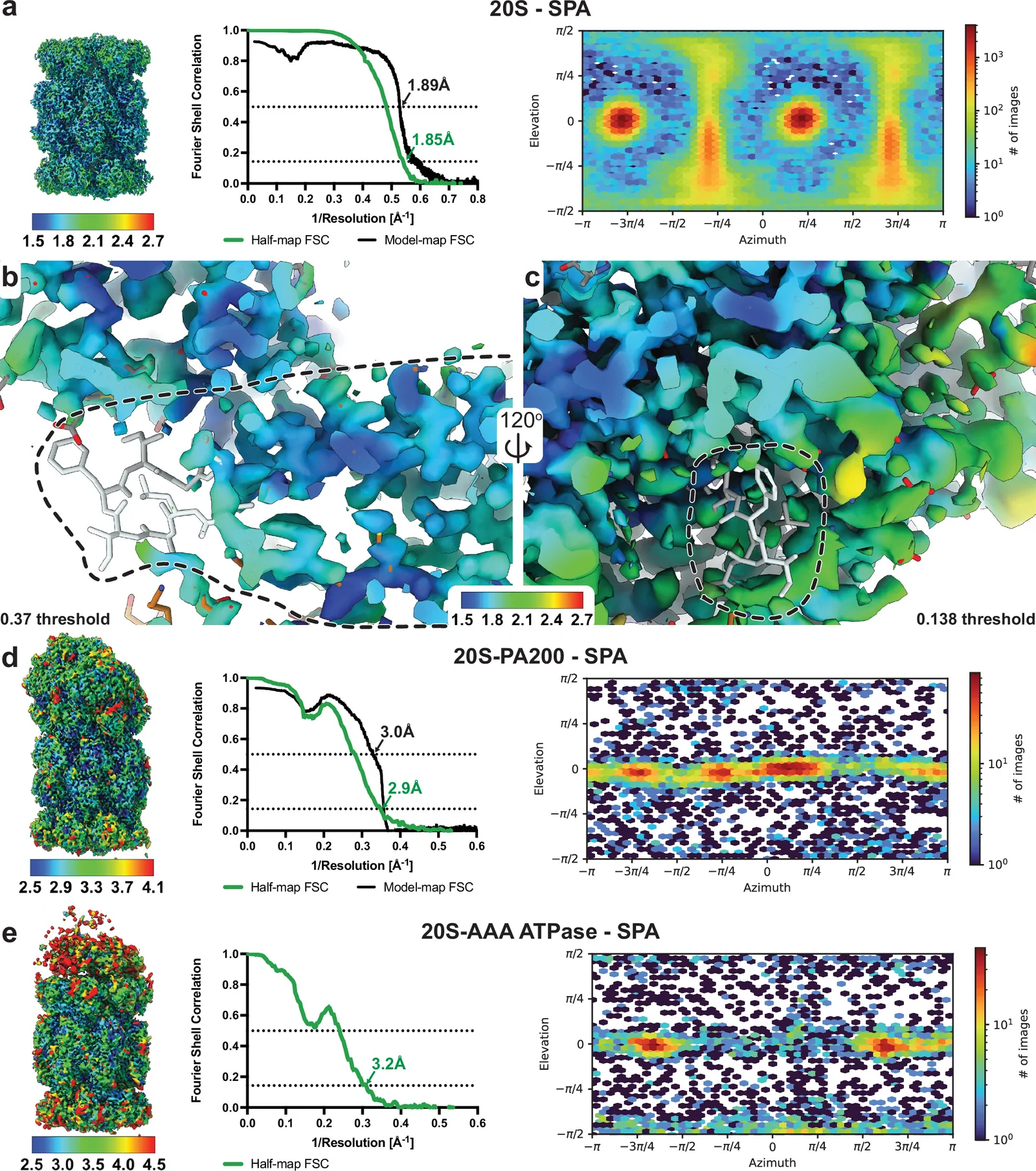
Properties of the final single-particle cryo-EM reconstructions (SPA). **a**, Local resolution map (left), Fourier Shell Correlation (FSC) plots (middle), and angular distribution map (right) for the 20S reconstruction. **b, c**, α4s FL-specific loop 77-82^α4s FL^ density is missing in the 20S human sperm core particle, confirming the presence of the α4s* splice variant. **b**, the density surrounding the missing 77-82^α4s FL^ loop is well-resolved at high resolution, indicating lack of variability in that region. **c**, the density surrounding the missing 77-82^α4s FL^ loop at a low map threshold value shows no residual signal corresponding to the 77-82^α4s FL^ residues, confirming that the loop is missing in human sperm proteasome. In **b, c**, the 20S human sperm core particle coordinates are shown as sticks, coloured by atoms. The α4s FL coordinates, modelled according to Materials and Methods, are shown as grey sticks. The density threshold values are indicated. The 77-82^α4s FL^ loop and its surrounding region are marked with dashed lines. **d, e**, Local resolution maps (left), Fourier Shell Correlation (FSC) plots (middle), and angular distribution maps (right) are shown for d) 20S-PA200 and e) 20S-AAA ATPase reconstructions. In **a, d**, both the gold-standard half-map FSC (green) and the model-map FSC (black) are shown with the resolution values at FSC = 0.143 (half-map FSC) and FSC = 0.5 (model-map FSC) indicated. In **e**, only the half-map FSC is shown as no coordinates were generated. In all panels, the maps are locally coloured and filtered according to the resolutions indicated on the colour key inset under each map. Source data

**Extended Data Fig. 6:**
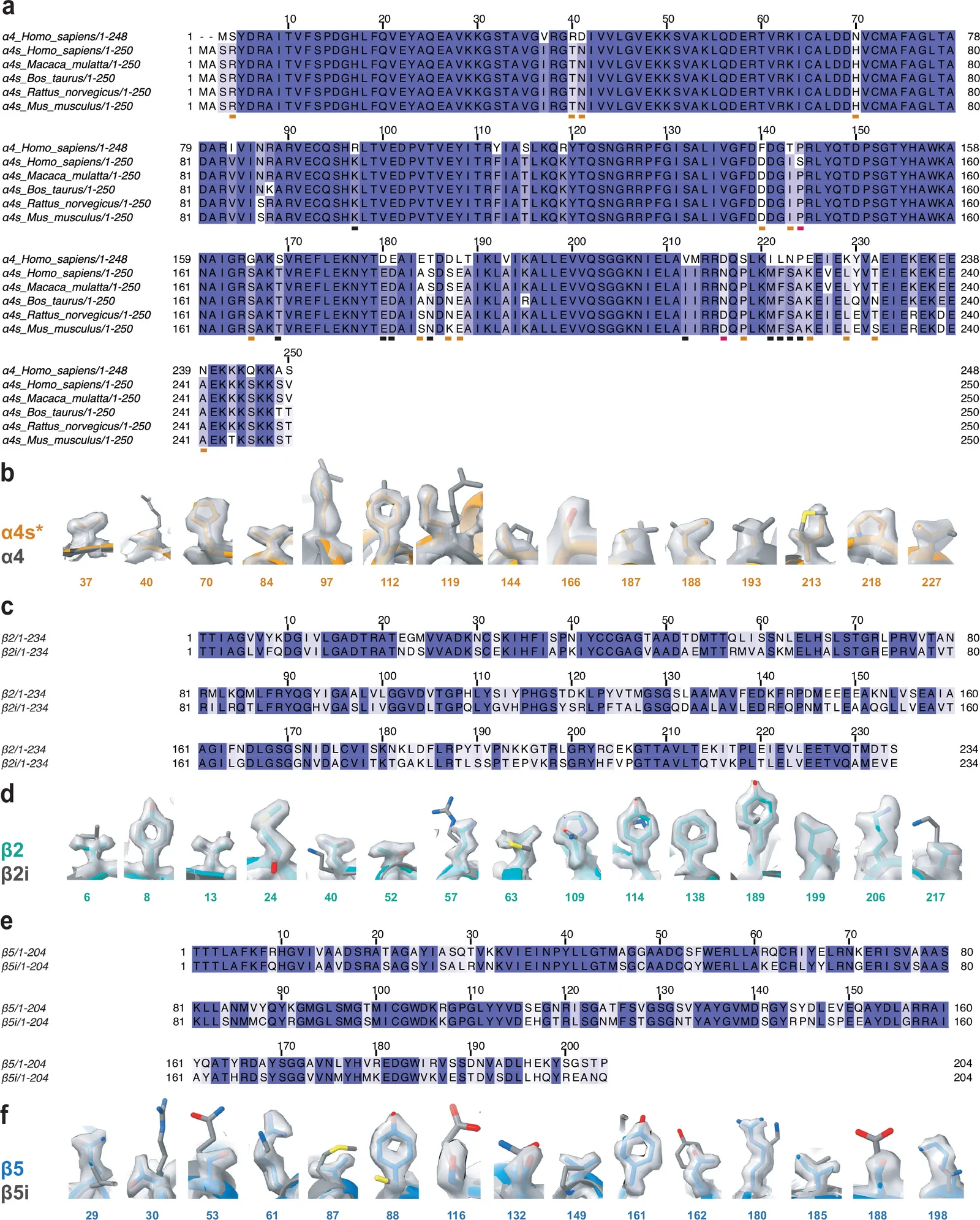
Subunit composition of the sperm proteasome. **a**, alignment of *Homo sapiens* α4 sequence (UniProt ID: O14818) with α4s sequences from five different species: *Homo sapiens* (α4s*, UniProt ID: Q8TAA3-5), *Macaca mulatta* (UniProt ID: A0A1D5Q503), *Bos taurus* (UniProt ID: A0AAA9S8Q9), *Rattus norvegicus* (UniProt ID: F1M6I7), *Mus musculus* (UniProt ID: Q9CWH6). The sequences are coloured by conservation. The underlines indicate solvent-exposed residues and are coloured by side-chain physicochemical class conservation between α4 with α4s: black – conserved, orange – different side-chain class conserved across all species, red – different side-chain class in some species. **b**, side-chain densities in the cryo-EM 20S proteasome map with modelled α4 (grey, PDB ID: 5LE5) and α4s* (yellow) side chains. The α4s* residue numbers are indicated. **c**, Sequence alignments of β2 (Uniprot ID: Q99436) and β2i (UniProt ID: P40306) coloured by conservation. The propeptide sequences are omitted. **d**, side-chain densities in the cryo-EM 20S proteasome map with modelled side chains of β2 (cyan) and β2i (grey, PDB ID: 7b12). **e**, Sequence alignment for β5 (UniProt ID: P28074) and β5i (UniProt ID: P28062), coloured by conservation. The propeptide sequences are omitted. **f**, side-chain densities for β5 (blue) and β5i (grey, PDB ID: 7b12). The residue numbers are indicated.

**Extended Data Fig. 7:**
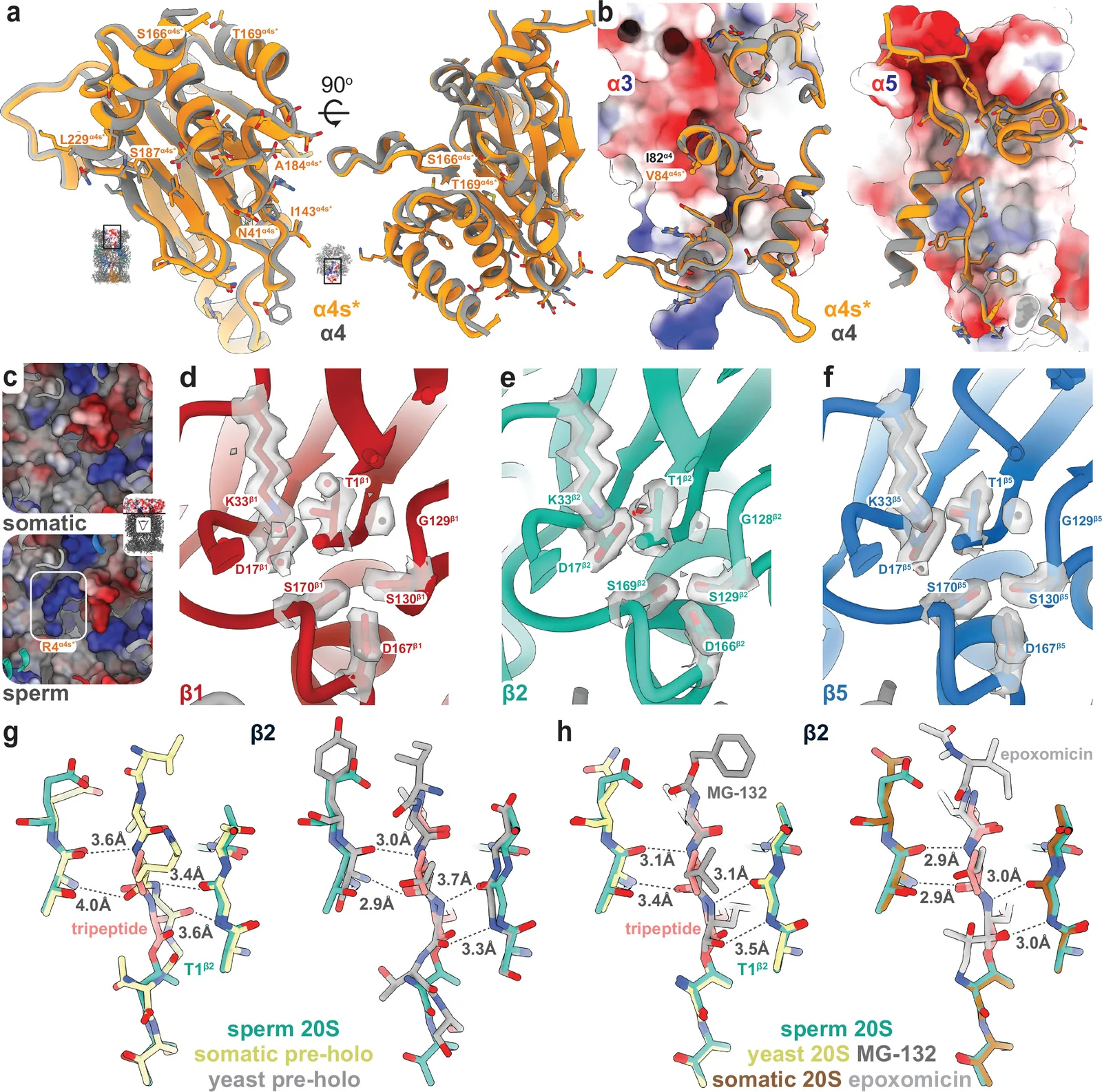
Structural features of the 20S sperm proteasome. **a**, α4s* structure (yellow) is highly conserved with α4 (grey, PDB ID: 5LE5). The models are shown in cartoon representation, and all non-conserved residues are shown as sticks. The solvent-exposed, non-conserved residues with a different physicochemical side-chain class are indicated, including the substitutions creating the uncharged patch on the surface of α4s*: Asp39^α4^ → Asn41^α4s*^, Thr141^α4^ → Ile143^α4s*^, Glu182^α4^ → Ala184^α4s*^, Asp185^α4^ → Ser187^α4s*^, and Lys227^α4^ → Leu229^α4s*^ **b**, interfaces between α4s* (shown as cartoon, yellow) or α4 (cartoon, grey) and α3 (left, shown as electrostatic surface) or α5 (right, electrostatic surface) subunits. All interfacial residues of α4s*/α4 are shown as sticks, and the Ile82^α4^ → Val84^α4s*^ substitution is indicated. **c**, the pores of somatic (PDB ID: 5LE5) and sperm 20S proteasome, shown as electrostatic surfaces and viewed from the catalytic chamber side, as indicated in the inset. R4^α4s*^ is indicated. **d, e, f**, sperm 20S proteasome active sites in β1 (red, **d**), β2 (cyan, **e**), and β5 (blue, **f**) subunits, shown as cartoons. Catalytic residues are shown as sticks, along with their corresponding map densities. The conserved catalytic water molecule is in grey. The corresponding residues in the somatic proteasome (PDB ID:5LE5) are shown as grey sticks. **g**, the β2 propeptides in human pre-holo somatic proteasome (yellow, PDB ID: 8IYI) and yeast pre-holo proteasome (grey, PDB ID: 8U6Y) are coordinated similarly to tripeptide substrate (pink) in sperm 20S core particle (cyan). **h**, proteasomal inhibitors, MG-132 (dark grey, bound to the yeast 20S proteasome, yellow, PDB ID: 5FGH) and epoxomicin (light grey, bound to the human 20S somatic proteasome, brown, PDB ID: 5LF0) are coordinated similarly to tripeptide substrate (pink) in sperm 20S core particle (cyan). In **g** and **h**, the dashed lines indicate hydrogen bonding (distances shown). In all panels, amino acids are marked using single-letter amino acid code.

To investigate the structure of sperm-specific proteasomes, we isolated native proteasomes from human sperm and performed cryo-EM analyses (Extended Data Fig. 4a–e). We obtained a 1.85-Å map of the sperm-specific 20S CP (Fig. 3a,b, Extended Data Figs. 4–6 and Table 2). The native sperm 20S proteasome did not contain immunoproteasome or thymoproteasome subunits. Specifically, we identified clear densities for the side chains corresponding to the canonical β5 and β2 subunits, instead of the immunoproteasome-specific β5i and β2i subunits (Fig. 3c and Extended Data Fig. 6c–f). Notably, our structure not only confirms that α4 is replaced by α4s in sperm (Fig. 3c and Extended Data Fig. 6a,b) but also shows clearly that the human sperm α4s subunit is an alternative splice variant (UniProt Q8TAA3-5) that lacks the α4s-specific 77–82^α4s^ loop, referred to as α4s* hereafter (Fig. 3c and Extended Data Fig. 5b,c). The density region adjacent to the missing loop is exceptionally well resolved and no residual signal corresponding to the loop is visible, corroborating that the region is compositionally homogeneous. α4s* shares a near-identical fold with the canonical α4 subunit (Extended Data Fig. 7a) (root-mean-square deviation (r.m.s.d.) between Cα atoms of α4s* and α4 (PDB 5LE5)^36^ = 0.567 Å). Accordingly, the overall fold of the sperm proteasome is highly conserved relative to the somatic form (r.m.s.d. between Cα atoms of an asymmetric unit = 0.538 Å). While the overall sequence conservation between the α4s* and α4 is very high (83.9% identity), 68% of the substituted amino acids are solvent exposed (Fig. 3d and Extended Data Fig. 7a). Most of these substitutions are conserved across mammals and render the surface of α4s* less negatively charged than that of α4 (Fig. 3d, dashed line, and Extended Data Fig. 6a). Intersubunit contacts between α4s* or α4 remain unchanged, except for a conserved I82^α4^-to-V84^α4s*^ substitution at the α4s–α3 interface (Extended Data Figs. 6a,b and 7b). Residues that interact with the proteasome assembly chaperone PAC4 were also preserved in α4s*, indicating that sperm 20S proteasome can also be assembled using the canonical machinery^37,38^. Similar to the canonical 20S CP, the gate of the sperm 20S is fully ordered and adopts a constricted conformation. However, a conserved N-terminal R^α4s*^ insertion creates a basic patch at the inner pore surface (Extended Data Figs. 6a and 7c), which may affect the kinetics of substrate entry or product release from the sperm proteasome. Lastly, the active sites of β1, β2 and β5 subunits closely match those of the mature somatic 20S CP, with propeptides cleaved and catalytic residues in the same conformation (Extended Data Fig. 7d–f).

**Table 2:** Statistics of cryo-EM data collection, data processing and model refinement

**Data collection**				
Microscope	TFS Titan Krios G4			
Acceleration voltage (kV)	300			
Energy filter	TFS SelectrisX			
Energy filter slit width (eV)	10			
Magnification	×105,000			
Detector	TFS Falcon 4i			
Physical pixel size (Å)	0.926			
Exposure time (s)	3			
Number of frames	61			
Total electron dose (e^−^ per Å^2^)	40			
Defocus range (µm)	1.0–2.0			
Number of micrographs collected	50,213			
Total number of particles extracted	897,368			
**Data processing**				
	**20S**	**20S–PA200**	**20S–AAA-ATPase**	**26S**
Protein Data Bank identifier	PDB 9TMN	PDB 9TMS	No model generated	No model generated
EM Data Bank identifier	EMD-56072	EMD-56074	EMD-56075	EMD-58412
Imposed symmetry	*C*_2_	*C*_1_	*C*_1_	*C*_1_
Final number of particles	222,000	6,748	4,207	1,842
Final resolution (Å), FSC = 0.143	1.85	2.9	3.2	4.3
Final resolution (Å), FSC = 0.5	2.1	3.5	4.1	9.1
Sharpening *B* factor [Å^2^]	−41	−28	−14	−26
Local resolution range [Å]	1.5–32.7	2.1–50.1	3.0–55.0	3.7–50.0
**Model refinement**				
Initial model	PDB 6RGQ	PDB 6KWY	–	–
Refinement package	PHENIX (version 2.0-5884) real-space refinement		–	–
Model resolution at FSC = 0.5 (Å)	1.89	3.1	–	–
Cross-correlation				
Mask	0.83	0.83	–	–
Volume	0.83	0.82	–	–
Model composition				
Nonhydrogen atoms	51,259	62,124	–	–
Protein residues	6,218	7,914	–	–
Waters	2,924	0	–	–
Ligands	MG: 3	IHP: 1	–	–
*B* factors mean (A^2^)				
Protein	32.32	73.72	–	–
Ligand	34.09	100.67	–	–
Waters	38.00	–	–	–
R.m.s.d.				
Bond lengths (Å)	0.005	0.003	–	–
Bond angles (^o^)	0.744	0.595	–	–
**Validation**				
MolProbity score	0.78	1.17	–	–
Clashscore	0.95	2.68	–	–
Poor rotamers (%)	0.06	0.7	–	–
Cβ outliers (%)	0	0	–	–
CaBLAM outliers (%)	1.05	1.8	–	–
Ramachandran plot (%)				
Favored	98.47	97.44	–	–
Allowed	1.53	2.56	–	–
Outliers	0.00	0.00	–	–

MG, magnesium ion; IHP, inositol hexakisphosphate.

**Fig. 4:**
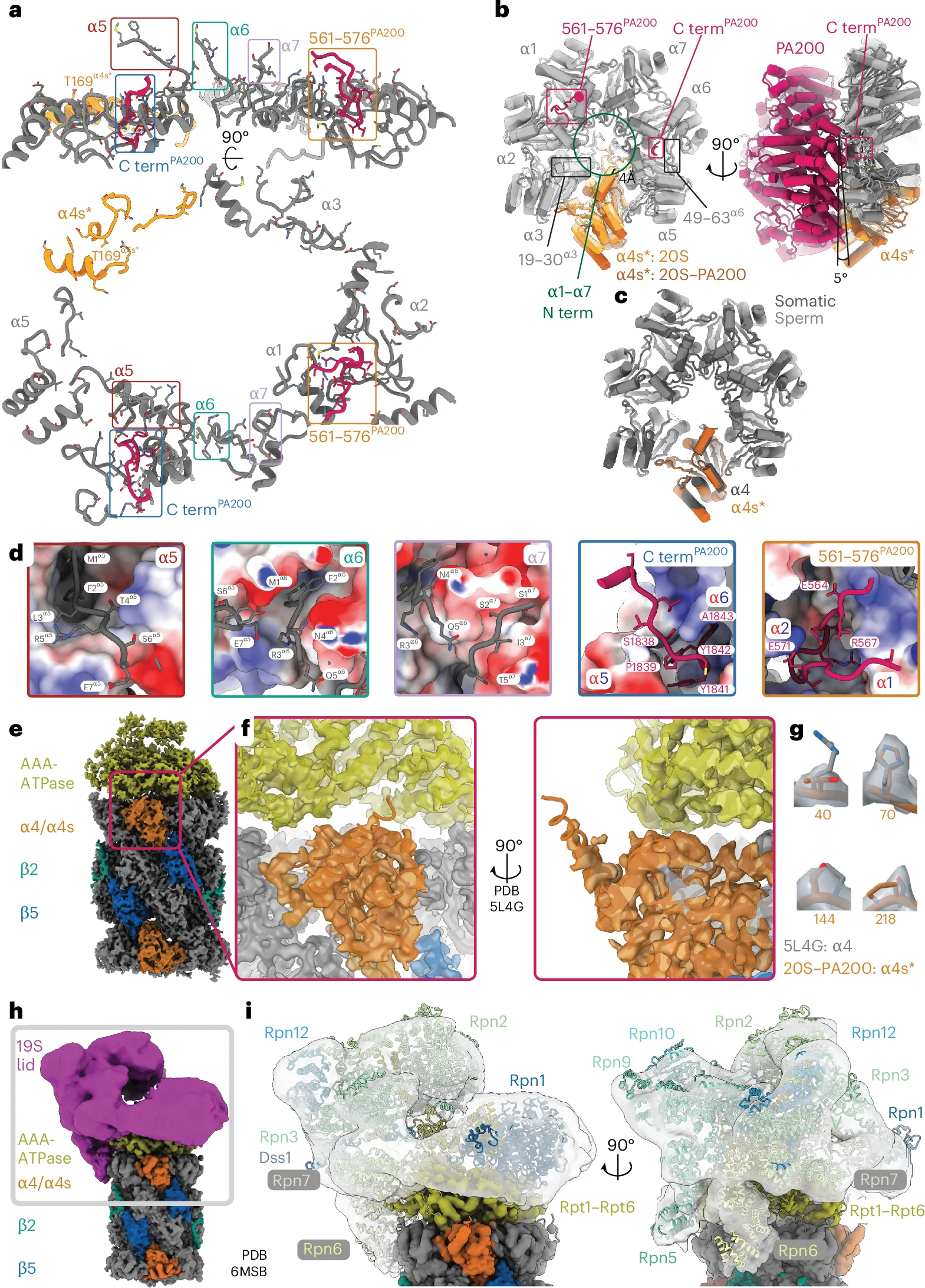
Interactions between the sperm 20S CP and proteasomal activators. **a**–**d**, Binding of PA200 to sperm 20S CP opens the proteasomal gate. To achieve this, the 561–576^PA200^ loop extends into the α1–α2 interface while the C-terminal 1838–1843^PA200^ (C term^PA200^) stretch displaces the α5–α6 subunits away from each other and shifts the 49–63^α6^ loop downward. Channel opening is further facilitated by disordering the gate-forming, N-terminal loops of all α-subunits (α1–α7 N term), the flexibility of the first α3 helix (residues 19–30^α3^) and incorporating the α5, α6 and α7 subunit N termini into the pockets on the PA200 surface. **a**, All the interactions between the α-subunits and PA200 activator in sperm 20S–PA200 structure. The α-subunit residues interacting with PA200 are shown as sticks. The secondary-structural features harboring these residues are shown as cartoons. The subunit names, as well as the only PA200-interacting residue in α4s* different to α4, T169^α4s*^ replacing S167^α4^, are indicated. The rectangles mark the structural features detailed in **d**. The main PA200 features interacting with the CP (that is, the PA200 C terminus and the 561–576 loop) are shown as purple cartoons and sticks. **b**, Conformational changes in the α-subunits occurring upon PA200 engagement led to a 4-Å horizontal shift and a 5° downward tilt of the α4s*. The structures are shown as cartoons with α-helices as tubes. The 20S–PA200 structure is opaque in darker tones, while the 20S is transparent and in brighter tones. The α4s is colored in orange tones. The key features undergoing conformational changes, as well as the shift and tilt of α4s*, are indicated. **c**, Comparison of somatic (PDB 6KWY; darker tones^39^) and sperm (brighter tones) 20S–PA200 structures at the α-ring. α4 (gray) and α4s* (dark orange) subunits are indicated. The r.m.s.d. between Cαs of all α-subunits is 0.599. **d**, The main features stabilizing the 20S–PA200 interface. The panels depict regions marked by rectangles in **a**. In α5, α6 and α7 panels, PA200 is shown as an electrostatic surface, while the 20S is shown as cartoons and sticks. In the C term^PA200^ and 561–576^PA200^ panels, the 20S subunits are shown as electrostatic surfaces, with names indicated, while PA200 is shown as purple cartoons and sticks. **e**, Cryo-EM map of the 20S AAA-ATPase complex isolated from human sperm. The AAA-ATPase (green), α4/α4s* (orange), β2 (cyan) and β5 (blue) subunits are indicated. **f**, The interface between AAA-ATPase subunits and α4/α4s*. The 20S–AAA-ATPase proteasome structure (PDB 5L4G) was rigid-body fitted in the map (model-map cross-correlation = 0.7759) and shown as cartoons. **g**, Side-chain densities in the sperm cryo-EM 26S proteasome map with modeled α4 (gray; PDB 5L4G) and α4s* (orange; 20S–PA200 coordinates) side chains. **h**, Cryo-EM reconstruction of the 26S proteasome isolated from human sperm. The segmented, local-resolution-filtered volume is shown. The same subunits as in **e**, as well as the remainder of the 19S activator (purple), are indicated. **i**, The fit of the entire 26S proteasome structure (PDB 6MSB, shown as cartoons) in the sperm 26S map. The 20S–AAA-ATPase density is opaque and colored as in **h**, while that corresponding to the remainder of the 19S activator is in transparent gray. The 19S subunits are indicated (model-map cross-correlation = 0.622).

We also solved the structure of the native 20S–PA200 complex at 2.9-Å resolution (Figs. 3e,f and 4a–d, Extended Data Figs. 4 and 5d and Table 2). Overall, the structure closely resembled that of the recombinant somatic 20S–PA200 (PDB 6KWY)^39^ (r.m.s.d. between Cα atoms = 0.639 Å), with an open α-ring gate that allows substrate entry (Fig. 3b,e). Gate opening is mediated by conformational changes in all α-subunits, equivalent to those in somatic 20S–PA200 (Figs. 3e–g and 4a–d). α4s* gets displaced (Fig. 4b), its 3–18^α4s*^ and 117–127^α4s*^ loops become disordered and the 49–63^α4s*^ loop shifts downward by 6 Å (Figs. 3g and 4b). All α-subunits form extensive contacts with PA200 (Fig. 4a,d). α4s* interacts with PA200 through numerous, mostly polar contacts which are conserved between α4 and α4s*, except for a conserved S167^α4^-to-T169^α4s*^ substitution (Figs. 3h and 4a and Extended Data Fig. 6a). Given the similarity of the two residues and the plethora of other interactions at the 20S–PA200 interface, we conclude that α4s* does not increase 20S affinity for the PA200 activator.

**Extended Data Fig. 8:**
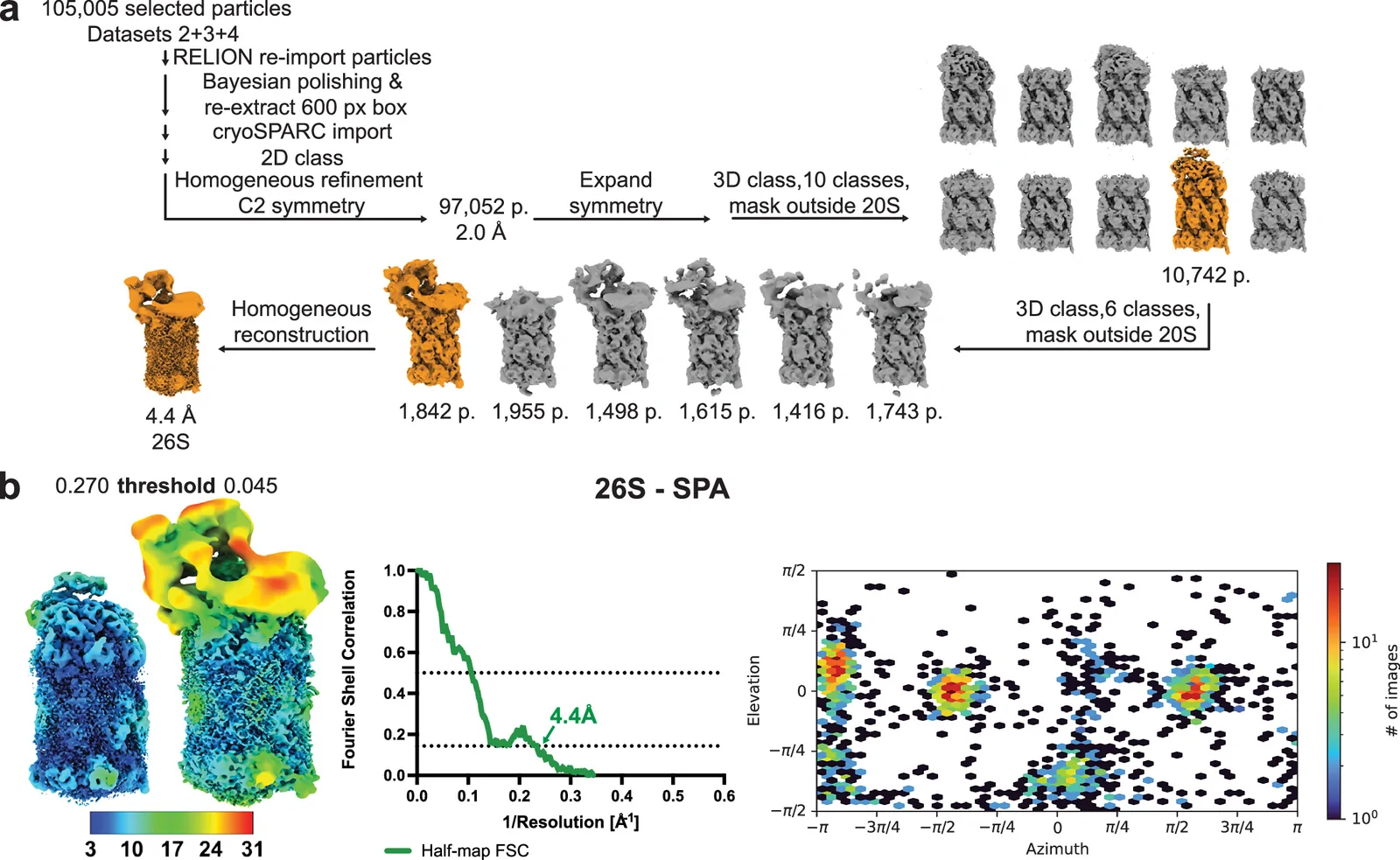
The processing pipeline and properties of the final single-particle cryo-EM reconstruction of the complete 26S proteasome from human sperm. **a**, single-particle cryo-EM data processing pipeline of the human sperm 26S proteasome. “p.” – particle number at corresponding processing step. The maps from key processing steps are shown. “105,005 selected particles Datasets 2+3+4” correspond to the particle selection from Extended Data Fig. 4f. **b**, local resolution maps (left), gold-standard half-map Fourier Shell Correlation (FSC) plot (middle), and angular distribution map (right) for the complete 26S human sperm proteasome. The resolution value at FSC = 0.143 is indicated. The local resolution maps show the same map at two threshold values (0.270 and 0.045, indicated above the maps) and are locally coloured and filtered according to the resolution of the colour key below the map. Source data

**Extended Data Fig. 9:**
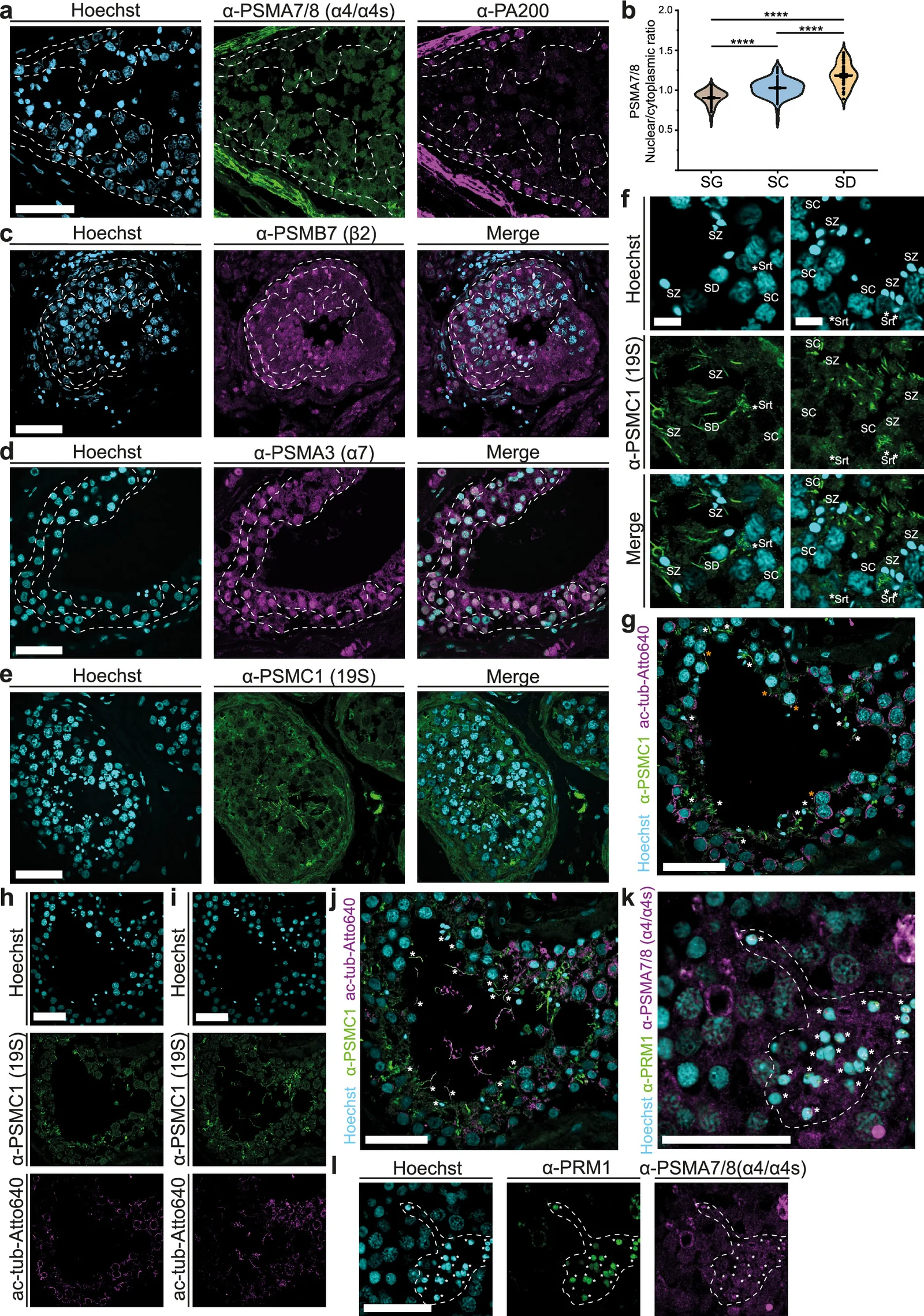
Distribution of proteasomal subunits and regulatory caps PA200 and 19S in human tissue during spermatogenesis. **a**, single-channel images for human seminiferous shown in Fig. 6b. PSMA7/8 (*α*4/*α*4 s) are shown in green, PA200 in magenta and DNA in cyan. White-dashed lines show different cell types. Scale bar = 50 *μ*m. **b**, Quantification of nuclear/cytoplasmic proteasome ratios in differentiating cells. Mean ± s.e.m.: Spermatogonia (SG) = 0.91 ± 0.01 (n = 83 cells, tissue from 3 donors); Spermatocyte (SC) = 1.03 ± 0.01 (n = 136 cells, tissue from 3 donors); Spermatid (SD) = 1.19 ± 0.02 (n = 70 cells, tissue from 3 donors). Statistical analysis: One-way ANOVA Bonferroni-corrected (****p < 0.0001). *p* = 2.6×10^−11^ (SG-SC); *p* = 1.9×10^−35^ (SG-SD); *p* = 2.9×10^−17^ (SC-SD). **c-d**, Distribution of proteasomal subunits in human testes. Dashed lines show different cell types. Scale bar = 50 *μ*m. DNA is shown in cyan. **c**, PSMB7 (β2) (magenta). Image representative of >50 seminiferous tubules, > 4 donors. **d**, PSMA3 (*α*7) (magenta). Image representative of >10 seminiferous tubules, 2 donors. **e**, Regulatory cap 19S subunit PSMC1 (green). Scale bar = 50 *μ*m. (>10 seminiferous tubules, from 2 donors imaged). **f**, PSMC1 localisation to spermatozoa (SZ) and SD tails, and nucleus of Sertoli cells (*Srt). Scale bar = 10 *μ*m. **g**, co-localisation of PSMC1 (green) with acetylated tubulin (magenta). Orange stars highlight putative SC cilia. White stars show co-localisation at SZ/SD tails. Scale bar = 50 *μ*m. Image representative of >10 seminiferous tubules, 2 independent experiments. **h**, individual channels for (*g)***. i**, additional example of PSMC1 and acetylated tubulin. Scale bar = 50 *μ*m. **j**, merge of images shown in (**i**). White stars show co-localisation. Scale bar = 50 *μ*m. **k**, nuclear PSMA7/8 clustering (magenta) relative to protamine (PRM1, green). SD/SZ are shown within white-dashed line. Scale bar = 50 *μ*m.**l**, single-channel images for merge shown in k. Scale bar = 50 *μ*m. Images are representative of >10 seminiferous tubules, from 2 donors. Source data

We identified a small fraction of 26S proteasomes in the 20S–PA200 sample. As 26S proteasomes were not detected by tomography within the nucleus (Fig. 1d), they likely originate from the sperm tail. This is supported by immunofluorescence and immunohistochemistry data showing enrichment of 19S subunits in the tail of both ejaculated human sperm and mature spermatozoa in seminiferous tubules of human testis (Extended Data Figs. 3e,f and 9e–j). We reconstructed the 20S–AAA-ATPase at 3.2-Å resolution with dynamic AAA-ATPase subunits resolved at 3.5–4.5 Å (Fig. 4e–g, Extended Data Fig. 5e and Table 2). Further processing revealed multiple, low-resolution proteasomal species with large, heterogeneous 19S-like activators, including a complete 26S proteasome (Fig. 4h,i and Extended Data Fig. 8a,b). The maps allowed rigid-body fitting of the somatic 26S proteasome structures^2,40^ into the sperm proteasome densities (Fig. 4f,i). Because of the resolution, we could not distinguish between the presence of α4 or α4s* in our map but we can exclude the presence of α4s. However, the fit allowed us to preclude major structural rearrangements in the sperm 26S relative to the somatic variant. Correspondingly, the α4/α4s* does not interact with the 19S cap in our map Fig. 4e–i).

### A tripeptide in the β2 subunit active site reveals insights into substrate recognition and PA200-mediated enhancement of trypsin catalytic activity

**Fig. 5:**
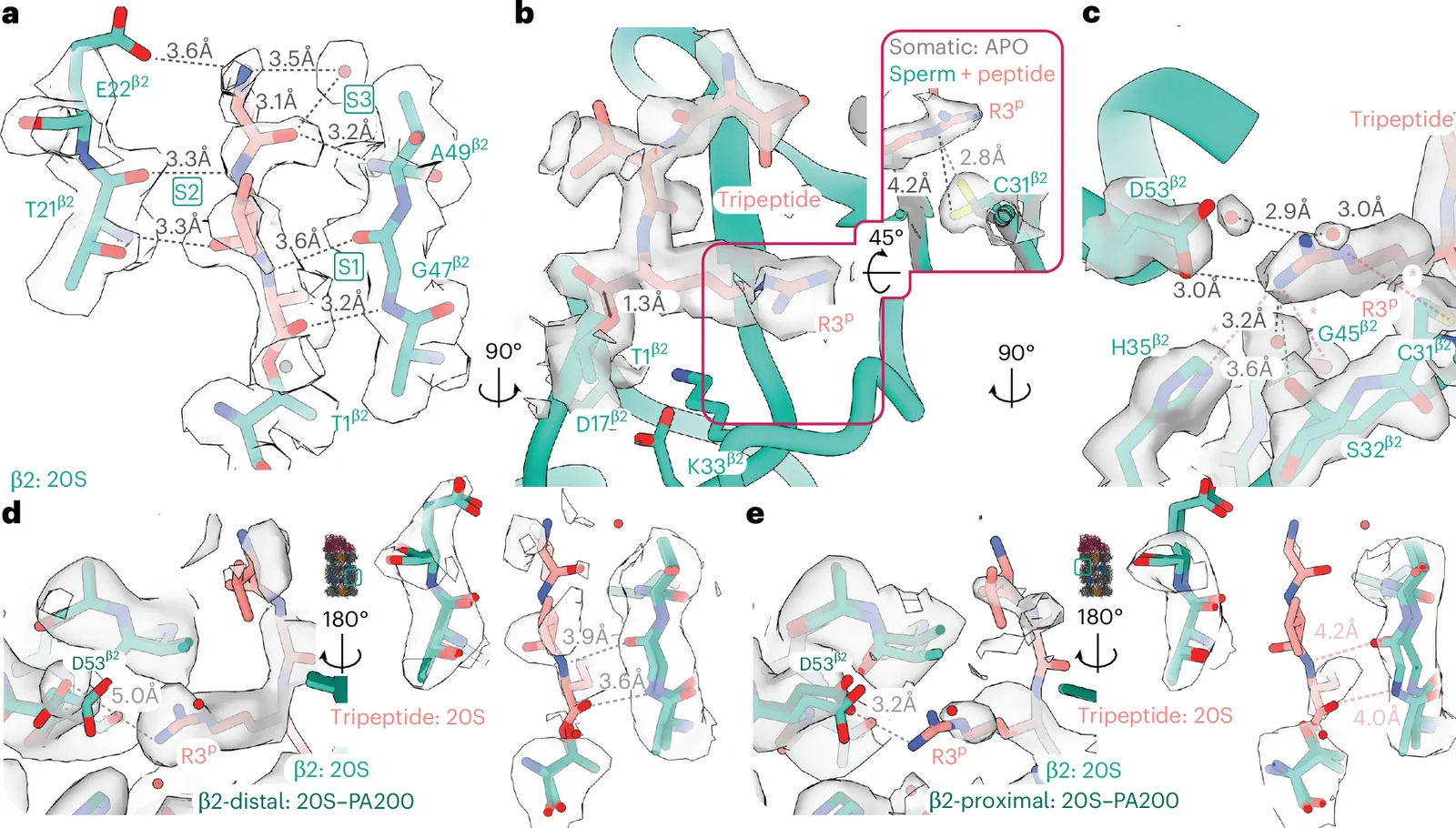
A peptide-like density in the β2 active site provides insights into substrate recognition and PA200-mediated enhancement of trypsin activity. **a**, The substrate tripeptide completes an antiparallel β-sheet in β2 subunit of sperm 20S CP by forming hydrogen bonds with T21^β2^, G47^β2^ and A49^β2^. The residues coordinating the substrate polypeptide backbone (cyan), as well as the substrate itself (pink), are shown as sticks with the corresponding densities shown as transparent surfaces. The substrate-coordinating S1, S2 and S3 positions are indicated. The dashed lines indicate hydrogen bonding (distances shown). The water molecule, previously identified as a catalytic nucleophile for deacylation, is shown in gray. **b**, Density protruding from the T1^β2^ β-hydroxyl group is identified as a tripeptide acyl-enzyme intermediate. It contains a clear arginine density (R3^P^), with its putative carbonyl carbon ~1.3 Å away from the nucleophilic oxygen, consistent with a single C–O covalent linkage^77^. The tripeptide is shown as pink sticks, covalently linked to the catalytic T1^β2^ β-hydroxyl. The corresponding density is shown as a transparent surface. The catalytic residues T1^β2^, D17^β2^ and K33^β2^ are indicated and shown as cyan sticks. The β2 subunit is shown as cyan cartoons. Inset: the C31^β2^ rearrangement is required to incorporate the arginine side chain. In gray, the somatic 20S proteasome structure in the APO state is shown (PDB 5LE5). The corresponding region in the sperm 20S CP is shown in cyan (the enzyme) and pink (the tripeptide). C31^β2^ and the tripeptide are shown as sticks, with their corresponding densities shown as transparent surfaces. The distance between the thiol group and N^δ^ of arginine is shown for both the sperm 20S CP (black dashed line) and the putative interaction in the APO somatic enzyme that would lead to a steric clash (gray dashed line). **c**, Coordination of the substrate arginine side chain in the β2 active site of sperm 20S CP. The arginine and its coordinating D53^β2^, S32^β2^ and water molecules are shown as sticks, with corresponding densities shown as transparent surfaces. Black and gray dashed lines indicate hydrogen bonds and salt bridges with distances shown. The distances between N^ω^ of arginine and H35^β2^ (4.0 Å), N^ω^ of arginine and G45^β2^ carbonyl (5.8 Å) and N^δ^ of arginine and C31^β2^ (4.2 Å) preclude interactions and are indicated as red stars and red dashed lines. **d**, The structure of the β2 subunit distal from the PA200 activator (β2-distal) is conserved with the sperm 20S CP β2. The main difference is the apparent rearrangement of the D53^β2^ side chain away from the arginine side chain. The β2 subunits from the sperm 20S structure (cyan), β2-distal from 20S–PA200 structure (dark cyan) and the tripeptide from the 20S structure (pink) are shown as sticks. The corresponding 20S–PA200 density is shown as a transparent surface. Inset: the position of β2-distal in the 20S–PA200 structure. Left: same view as in **c**. The distance between the N^ω^ of arginine and the D53^β2^ side chain in β2-distal is marked with a dashed line and indicated. Right: conformational conservation of the substrate-coordinating S1, S2 and S3 sites between the sperm 20S β2 subunits and β2-distal of the sperm 20S–PA200. The distances between the substrate backbone and S1 residues are marked with gray dashed lines and indicated. **e**, Engaging PA200 causes conformational changes in the β2 subunit proximal to the activator (β2-proximal), expanding the substrate-binding site to facilitate product release. The same features as in **d** are shown for the β2-proximal subunit of sperm 20S–PA200. The tripeptide from the 20S structure is shown for reference, as only residual substrate density is present in the β2-proximal site. The distances between the hypothetical substrate and the key coordinating residues are marked with dashed lines and indicated. In **d**,**e**, the map is shown at a 0.291 density threshold level.

In the β2 subunit of the sperm 20S CP, we observed an unexpected, continuous and branched density protruding from the nucleophilic T1^β2^ β-hydroxyl group toward the substrate-coordinating S1, S2 and S3 sites (Fig. 5a–c). The interaction with the S1–S3 sites resembled that of propeptides or peptide-like inhibitors^1,36,41–43^ (Extended Data Fig. 7g,h). The conformation of the catalytic T1^β2^ and lack of any polypeptide density extending upstream of its carbonyl carbon precluded the density from being a propeptide (Extended Data Fig. 7g). The map also did not correspond to known proteasomal peptide-like inhibitors forming hemiacetal linkages^41^ (Extended Data Fig. 7h). Instead, we noticed that the density protruding from Cα adjacent to T1^β2^ was very extended, in line with the specificity of the β2 subunit for cleaving peptide bonds at the C terminus of large, basic amino acid residues, primarily arginine and lysine. Unlike lysine, the arginine side chain fitted the density exceptionally well; thus, we modeled the density as an arginine-containing tripeptide, which fitted with very good geometry (Fig. 5a–c). Regardless of its identity, the density revealed the coordination of a native-like substrate in the S1 pocket. The arginine side chain is stabilized by the D53^β2^ carboxyl group, S32^β2^ carbonyl oxygen and a network of water molecules (Fig. 5c). S1 pocket broadening is required to engage the substrate. This is achieved by rearranging the C31^β2^ side chain (Fig. 5b, inset). In our map, the density allows modeling of the C31^β2^ side chain in both conformations, indicating that the substrate is present with partial occupancy (Fig. 5b). The remaining residues of the tripeptide bind the S2 and S3 pockets in a configuration similar to that seen in inhibitor-bound and propeptide-containing structures inhibitors^1,36,41–43^ (Fig. 5a and Extended Data Fig. 7g,h). Interestingly, the catalytic water that was proposed to perform a nucleophilic attack on the ester linkage is not positioned at an appropriate geometry in relation to the carbonyl group to resolve the acyl-enzyme intermediate^44^ (Fig. 5a). This likely explains trapping of the intermediate in the active site.

Engaging the PA200 cap increases the proteasome trypsin activity at least threefold^26,45^. Aligning the 20S β2 subunit to the corresponding regions in the 20S–PA200 structure reveals the possible reasons for this (Fig. 5d,e). In particular, the structure is almost entirely conserved with 20S in the activator-distal β2 subunit, including the presence of the peptide density (Fig. 5d). In turn, in the activator-proximal β2 subunit, the D53^β2^-containing helix is tilted away from the putative product, while the β-strands of S2 and S3 sites widen apart. These conformational changes likely reduce the coordination strength and allow efficient release of the product. In line with this, only the residual signal is detected in the S1 pocket of the activator-proximal β2, while the S2 and S3 positions are unoccupied (Fig. 5e), indicating an increased peptide processing rate.

Altogether, our 20S structure reveals crucial insights into the binding of a native-like peptide into the trypsin catalytic site of the human proteasome and uncovers a distinct substrate recognition mechanism by β2. Moreover, the structure of the 20S–PA200 proteasome from a native source explains the molecular determinants of trypsin activity enhancement upon cap engagement.

### Human proteasome lacunae with 20S and PA200 form during the spermatid stage of spermatogenesis

**Fig. 6:**
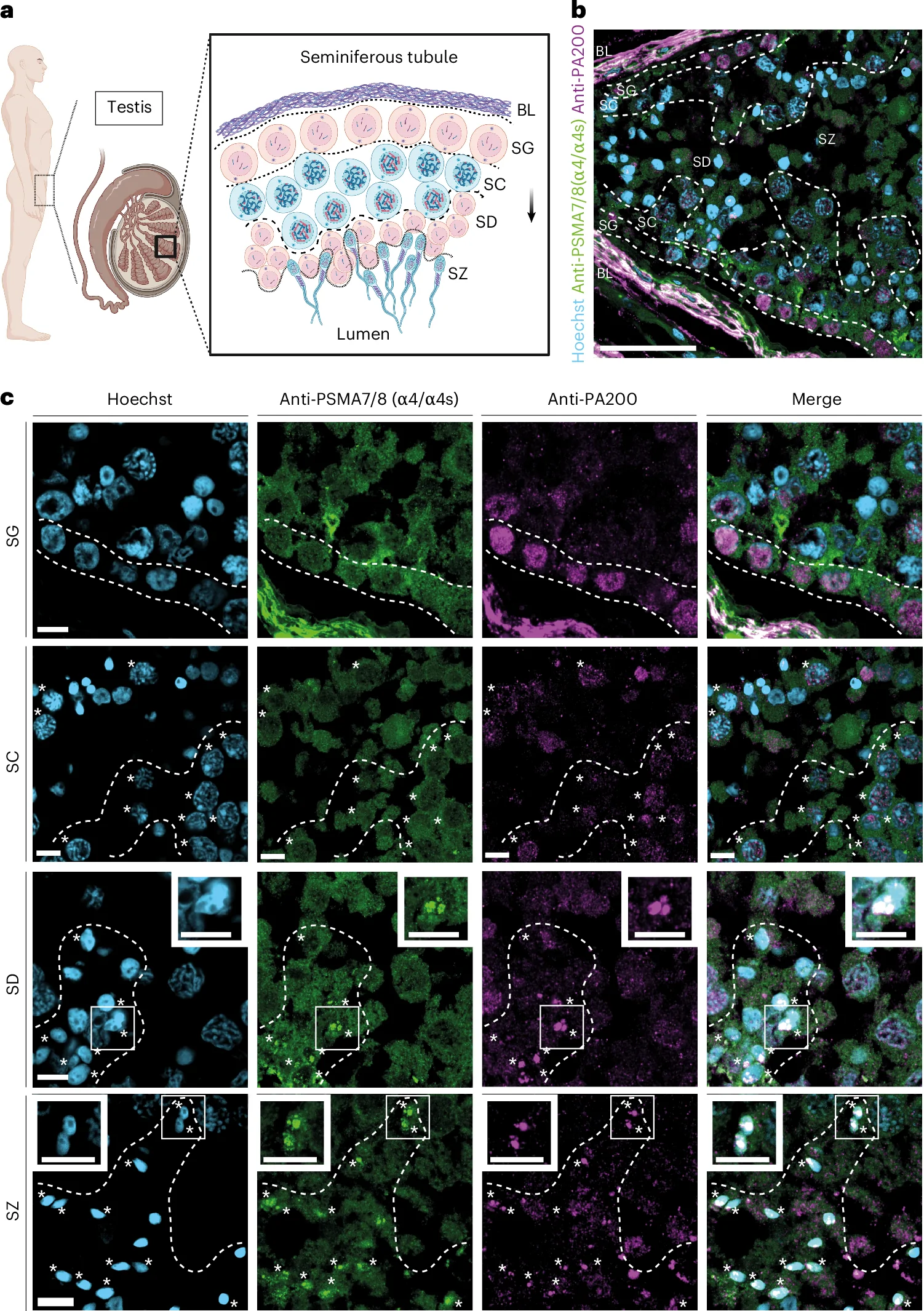
Proteasome reorganization and clustering during human sperm-cell differentiation. **a**, Diagram showing the organization of seminiferous tubules in human testes. Cells within seminiferous tubules are surrounded by a basal lamina (BL). Spermatogenesis progresses from the outer region of the tubule toward the central lumen. Spermatogonia (SG), localized close to the BL, undergo two rounds of meiosis. Spermatocytes (SC; meiotic cells) occupy the median region of the tubules and postmeiotic haploid spermatids (SD) lie close to the lumen, where they differentiate into mature spermatozoa (SZ). **b**, Confocal microscopy image showing an overview of a seminiferous tubule from a healthy human donor. Immunohistochemistry staining shows distribution of proteasome subunit PSMA7/8 (α4/α4s) in green, proteasome adaptor PA200 in magenta and DNA (Hoechst) in cyan. Individual channels for this image are shown in Extended Data Fig. 9. White dashed lines represent boundaries between different cell types. Scale bar, 50 μm. **c**, Distribution of PSMA7/8 (α4/α4s) and PA200 at different differentiation stages in human testes. Panels represent zoomed-in regions of the overview shown in **b**. Each cell type (SG, SC, SD and SZ) is represented within white dashed line boundaries and/or by white stars. Insets: zoomed-in regions of the areas inside the white rectangles in the SD (right) and SZ (left) panels. Scale bars, 5 μm. Images are representative of >50 seminiferous tubules from >4 donors. Panel **a** created in BioRender; Dos Santos, A. https://BioRender.com/rb78rwd (2026).

**Extended Data Fig. 10:**
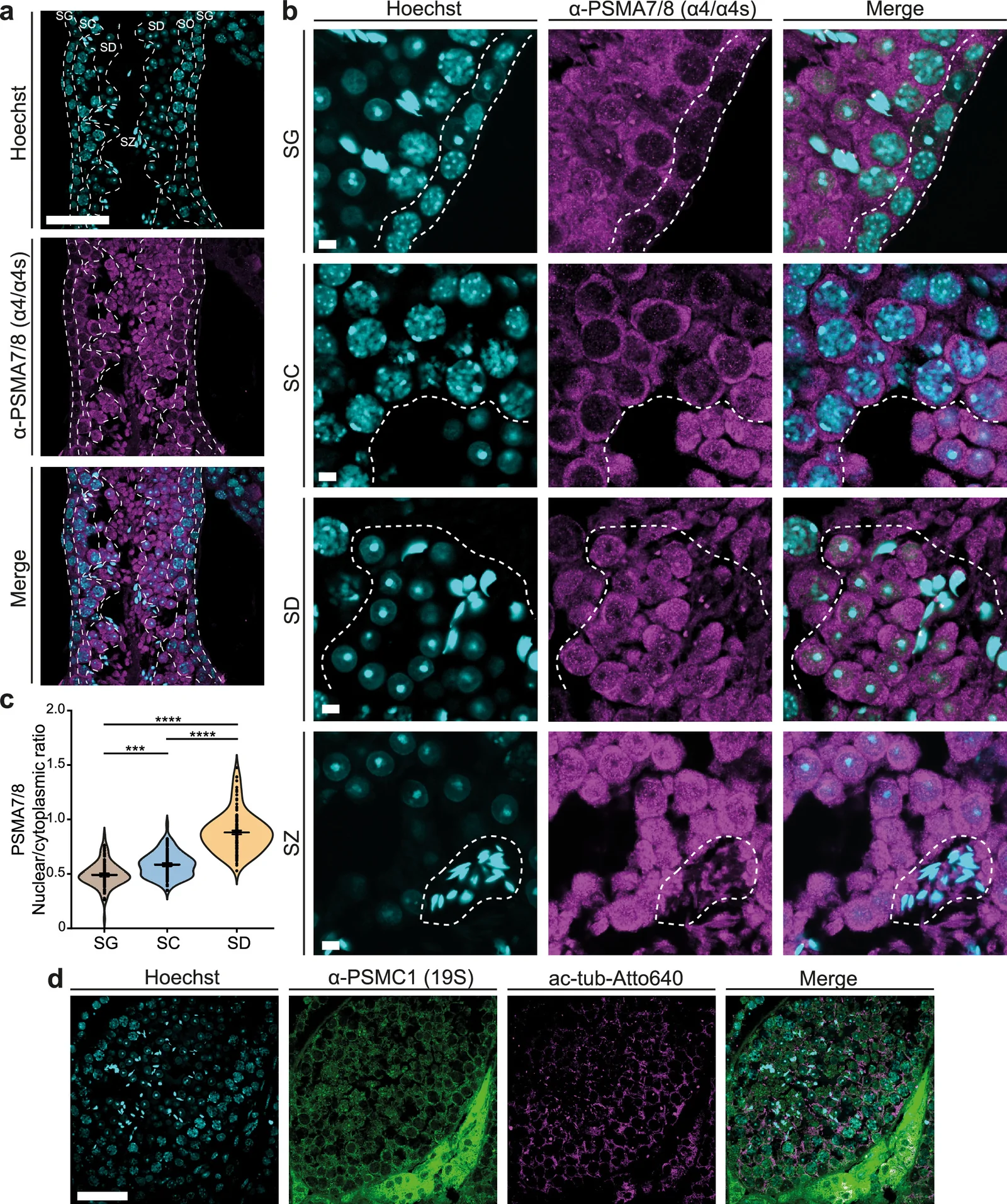
Distribution of proteasomal subunits and regulatory cap 19S during mouse spermatogenesis. **a**, confocal microscopy image showing a seminiferous tubule from mouse FFPE tissue and distribution of proteasome PSMA7/8 (*α*4/ *α*4 s) subunits. White-dashed lines show tissue sub-compartments enriched for different germ cells: Spermatogonia (SG), Spermatocytes (SC), Spermatids (SD) and spermatozoa (SZ). Scale bar = 50 *μ*m. **b**, zoom-in region of mouse seminiferous tubules showing proteasome subunit PSMA7/8 (*α*4/ *α*4 s) distribution during differentiation. White-dashed lines show regions with the different cell types: SG, SC, SD and SZ. Scale bar = 10 *μ*m. **c**, quantification, from confocal immunohistochemistry mouse data, of nuclear/cytoplasmic proteasome ratios in germ cells. Mean ± s.e.m. values are as follows: SG = 0.49 ± 0.01 (n = 80 cells, 3 independent experiments); SC = 0.58 ± 0.01 (n = 88 cells, 3 independent experiments); SD = 0.88 ± 0.02 (n = 112 cells, 3 independent experiments). Statistical analysis: One-way ANOVA Bonferroni-corrected *p* = 2.7 ×10^−4^ (SG-SC); *p* = 2.3 ×10^−48^ (SG-SD); *p* = 1.7 ×10^−34^ (SC-SD), shown as ***p < 0.001; ****p < 0.0001. **d**, distribution of PSMC1 subunit of regulatory cap 19S (green) in mouse sperm testis, relative to acetylated tubulin (magenta). Scale bar = 50 *μ*m. Images are representative of >10 seminiferous tubules, from 2 independent experiments. Source data

To investigate how the distribution and levels of proteasome complexes change during human sperm-cell differentiation (Fig. 6a), we obtained healthy human testis tissue and performed immunohistochemistry, followed by confocal microscopy (Fig. 6b and Extended Data Fig. 9a–l). The 20S subunits PSMA7/8 (α4/α4s), PSMB7 (β2) and PSMA3 (α7) were present in all cells at the different stages of differentiation from spermatogonia to spermatozoa (Fig. 6b,c and Extended Data Fig. 9a–d). Interestingly, there was an enrichment in nuclear 20S CPs as differentiation progressed, with notably higher levels in the nucleus of spermatocytes and spermatids, relative to spermatogonia (Fig. 6c and Extended Data Fig. 9b). Furthermore, large clusters of 20S were clearly present in the nuclei of both spermatids and mature spermatozoa in human tissue (Fig. 6c, stars in SD and SZ panels), which coincided with the presence of protamine in the nucleus (PRM1) (Extended Data Fig. 9k,l). These clusters, similar to what we described in ejaculated sperm (Fig. 2e–j), appeared to preferentially form in nuclear regions without DNA (that is, lacunae; Fig. 6c, SD and SZ stars). In mouse testes, we observed a similar pattern, with significant enrichment in nuclear 20S CP as differentiation progressed from the spermatogonia to the spermatid stage (Extended Data Fig. 10a–c). However, proteasomal clustering within the nuclei of postmeiotic cells was markedly reduced compared to human tissue, particularly in mature spermatozoa (Extended Data Fig. 10b, SD and SZ panels, dashed lines). In somatic cells, PA200 is enriched in the nucleus, where it has been shown to facilitate histone degradation^26–29^. Our human tissue data confirm the presence of PA200 in nuclei of both spermatogonia and differentiating sperm cells. In human spermatids and spermatozoa, PA200 formed clusters that colocalize with 20S (Extended Data Fig. 9a and Figs. 2j and 6c).

Next, we investigated the localization of 19S in human tissue, as neither our light microscopy nor our cryo-ET data from ejaculated sperm cells showed the presence of this adaptor in the nucleus (Fig. 1d and Extended Data Fig. 3e,f). Our data show that 19S is highly enriched in the lumen of seminiferous tubules (Extended Data Fig. 9e–j) where it colocalizes with acetylated tubulin in the tail of mature spermatozoa and the growing tail of spermatids (Extended Data Fig. 9f–j). Interestingly, we found that, in Sertoli cells within human testis tissue, the 19S adaptor is enriched in the nucleus (Extended Data Fig. 9e,f,h). We also observed localization of 19S in spermatocyte protrusions, positive for acetylated tubulin, which could represent primary cilia^46^ (Extended Data Fig. 9g, orange stars). Selective enrichment of 19S or 26S proteasomes at these regions would provide a distinct proteostatic environment relative to the nucleus of differentiating cells. Contrastingly, in mice, we detected lower signal for 19S in the tail of mature spermatozoa (Extended Data Fig. 3c) and we could not observe colocalization with acetylated tubulin in differentiating spermatids or spermatozoa (Extended Data Figs. 3c and 10d). This further highlights differences between organisms and suggests that requirements for paternal proteasomes may differ between species after fertilization.

Overall, our human tissue data reveal that clustering of both the 20S sperm proteasome and the activator PA200 in nuclear lacunae occurs from the spermatid stage, which coincides with DNA protamination. Conversely, in mouse tissue, clustering is minimal in spermatids and was not detected in spermatozoa using immunohistochemistry, further emphasizing differences between the two species. Lastly, 19S was mostly present in the tail of human spermatids and spermatozoa.

## Discussion

Previous studies indicated that nuclear proteasomal activity is indispensable for chromatin remodeling and sperm development^27^. However, the presence, localization and function of nuclear proteasomes in sperm cells remained unexplored. The pioneering work of Haraguchi et al.^31^ proposed the presence of proteasomes within nuclear cavities of both rat and human sperm heads. Chemes and Alvarez Sedo^32^ further annotated these membraneless cavities in sperm DNA as lacunae and linked them to dysregulated proteolytic activity^32^. Here, we show that human sperm nuclear lacunae are large assemblies of proteasome complexes and we characterize their molecular architecture.

Subtomogram averaging of proteasomes in situ has previously revealed important aspects of cytoplasmic and nuclear proteasome biology^47–49^. In this study, we used cryo-ET and subtomogram averaging to establish that nuclear sperm lacunae are populated by a mixture of 20S CP and 20S–PA200 proteasomes (Fig. 1, Extended Data Figs. 1 and 2, Supplementary Video 1 and Table 1). Volume imaging and super-resolution STORM microscopy further revealed that proteasome-rich lacunae vary significantly in size and morphology (Fig. 2a–g) and coincide with areas devoid of DNA, in agreement with our cryo-ET data (Figs. 1a,b and 2h–j). Furthermore, we show that nuclear proteasome clustering is conserved in mice but reveal species-specific differences, whereby mouse sperm lacunae are markedly smaller and fewer in number (Extended Data Fig. 3b,c).

In sperm proteasome, the canonical α4 subunit is replaced with the testis-specific α4s. This substitution is conserved across species and is essential for sperm differentiation and fertility^23^. Our near-atomic-resolution cryo-EM structure of the human 20S proteasome CP (Fig. 3a–d and Extended Data Figs. 5–7) reveals the exclusive presence of a specific splice variant in human, which we term α4s*. Consistent with our structural observations, this splice variant is the predominant α4s isoform transcribed during spermatogenesis^50^ and a published proteomics dataset from human sperm cells identified unique peptides for this variant but not for full-length (FL) α4s^51^. The main amino acid substitutions in α4s*, relative to α4, are located on the external surface of the 20S (Fig. 3d and Extended Data Figs. 6a,b and 7a,b), suggesting a possible role in recruiting specific factors during spermatogenic differentiation. This would be consistent with reports that α4s deletion leads to meiotic arrest and data showing preferential interactomes of α4s proteasomes purified from testes^9,22,24,25^. As proteasomes in mature sperm nuclei are free of such factors, as shown by our cryo-ET data (Fig. 1), it will be important to investigate proteasome structures in differentiating germ cells to understand the nature of α4s-mediated interactions.

Previous studies reported that β1i, β2i and β5i immunosubunits replace the canonical proteolytic β-subunits in the sperm proteasome^27^. However, our sperm 20S proteasome structure did not contain immunoproteasome or thymoproteasome subunits (Fig. 3c and Extended Data Fig. 6c–f).

Remarkably, our high-resolution 20S structure consists of an arginine-containing tripeptide covalently linked to the catalytic threonine of the β2 subunit (Fig. 5). There are several possible reasons for this. First, our gentler purification method, compared to previous studies^36,52^, preserves proteasomes in their active state throughout the process and may also supply substrate up to the cryo-EM sample preparation step (Extended Data Fig. 4a–e and Methods). In particular, nuclease treatment at the beginning of the purification, combined with a reducing environment throughout the process, likely released monomeric protamines. These small peptides could enter the 20S CP for degradation and remain visible in the active site because of low proteolytic activity of the 20S CP^53^. While the sperm 20S gate in our structure adopts a closed conformation, various studies indicate that it can be opened by oxidized and unfolded proteins with exposed hydrophobic patches^54–57^ or by peptides containing the C-terminal HbYX (hydrophobic–tyrosine–any amino acid) motif^58,59^. Indeed, although protamines are highly positively charged proteins, they do include a version of the XbYX motif in their sequence (residues 15–18, YYR; UniProt P04553). During proteolysis by the β2 subunit, the protamine degradation product containing the YYR motif at the C terminus could be formed and released, opening the proteasomal gate. Similarly, an early study suggested that polycationic substances, including protamines, enhance proteasomal activity^60^ and more recent data propose proteasomal activation by polyamines^61^. This supports the idea that protamines could be degraded by the sperm 20S CP. On the basis of our map, we hypothesize that the active site of the β2 subunit might be occupied by a protamine fragment, for example, CCR motif (residues 6–8) or other Arg-containing motifs. However, further work will be required to validate this hypothesis and determine the biochemical identity of the peptide. The sperm proteasome gate consists of an N-terminal insertion in α4s* that positions a positively charged R4^α4s*^ at the inner surface, whereas the same region is neutral in the canonical variant (Extended Data Fig. 7c). This may represent an adaptation for processing cationic protamine substrates, affecting both substrate access and the release of cleavage products. In mature spermatozoa, protamines are crosslinked by disulfide bridges, preventing their degradation^62^. The process may instead be initiated after fertilization.

Post-translational modifications at the pore region or in the active site, acquired during sperm-cell differentiation, could also affect product entry or release. Although close inspection of these regions did not reveal additional densities protruding from side chains, further biochemical analysis and mass spectrometry studies might be needed to clarify this matter.

The peptide observed in the active site reveals a native substrate in the β2 subunit. Unexpectedly, C31^β2^, G45^β2^ and H35^β2^ do not coordinate the substrate (Fig. 5c), distinguishing β2 from other catalytic subunits^1,41,52^. This is likely because of the distal positioning of the positively charged group on the substrate side chain. In yeast, a G45^β2^-to-A45^β2^ substitution does not affect cell growth, subunit folding or ligand binding, consistent with the proposal that G45^β2^ is not necessary for substrate recognition^63^. The position of the water molecule previously proposed to act as a nucleophile resolving the acyl-enzyme intermediate^1,41,64^ is suboptimal for efficient nucleophilic attack. A similar conclusion was drawn in a study reporting the high-resolution structure of a somatic proteasome in complex with inhibitors^36^. While Schrader et al. proposed that a water molecule hydrogen-bonded to the active site Y169 and T21 has a critical, yet unknown role, the corresponding water molecule does not coordinate the peptide in our structure. Altogether, the mechanism of resolving the acyl-enzyme intermediate requires further study and may involve conformational changes, likely facilitated by engagement of proteasomal activators.

In our 20S–PA200 structure, the peptide density was nearly absent in the activator-proximal β2 subunits, suggesting that it represents a trapped catalytic intermediate in the 20S CP. Decreased substrate occupancy is accompanied by conformational changes in the activator-proximal β2 active site of 20S–PA200 structure (Fig. 5e). While previous work on the canonical 20S–PA200 structure^45^ identified rearrangements of the trypsin site upon PA200 binding, the presence of a substrate in the active site revealed the mechanistic implications of these conformational changes. To explore this, we directly compared the effect of conformational changes in PA200-distal and PA200-proximal β2 subunits on substrate binding in the single map (Fig. 5d,e). The conformation-dependent differences in substrate processing rates proposed here may also apply to canonical proteasomes because of the conservation of the β2 subunit between sperm and somatic 20S–PA200 proteasomes.

Previous studies have suggested that α4s may favor association with the PA200 activator^24,25^. However, in our structure the residues at the α4s*–PA200 interface are largely conserved relative to those in the α4 subunit (Figs. 3h and 4a–d and Extended Data Fig. 6a–c). Our data, therefore, suggest that α4s* may not alter the intrinsic binding preferences of the 20S proteasome toward PA200, in agreement with Zivkovic et al.^22^.

Interestingly, in human testes, the 19S AAA-ATPase subunit PSMC1 was enriched in the tail of differentiating spermatids and mature spermatozoa (Extended Data Fig. 9f–j). Furthermore, both PSMC1 and PSMD2 (Rpn1), other 19S subunits, localized exclusively to the tail of ejaculated sperm (Extended Data Fig. 3e,f). This enrichment was not shared by 20S subunits (Fig. 6b,c and Extended Data Fig. 9a–d). Several studies have proposed that α4s confers sperm 20S preference for 19S^22,65^. However, our structural data do not support this notion, as there are no interactions between α4/α4s* and AAA-ATPase subunits of 19S in our map (Fig. 4e–g). Therefore, it remains unclear how specific nuclear clustering between 20S and PA200, but not between 20S and 19S, occurs in the sperm head during differentiation. The lack of 19S complexes in the nucleus and their exclusive localization at the human sperm tail may be because of the presence of additional, so far undetermined factors, which could be recruited by α4s*; however, further work is required to validate this hypothesis.

Our human primary tissue data place proteasomal cluster formation concurrent with protamination and hypercondensation of DNA in round spermatids (Extended Data Fig. 9k,l). At this stage, histones are replaced first with transition proteins and subsequently with protamines^66,67^, a process that is dependent on proteasomal activity^27^. In agreement with this, we show that postmeiotic nuclear proteasome clusters in human tissue colocalize with the PA200 activator (Fig. 6b,c), which confers specificity for histones targeted for removal through hyperacetylation^27,66^. The replacement of histones with protamines ultimately results in hypercompaction of chromatin^68^. Therefore, it is possible that, during DNA compaction, proteasome complexes become excluded from the DNA, leading to the formation of lacunae. In our cryo-ET and STORM data of spermatozoa nuclei (Figs. 1 and 2), proteasome-containing lacunae are indeed found deeply embedded in the highly condensed protamine–DNA phase. They may be retained there because of extreme DNA condensation, protamine crosslinking and disrupted nucleocytoplasmic transport^69^.

Various studies have proposed roles for the sperm proteasome during and after fertilization^15,18,65^. Our data did not reveal proteasome complexes in the acrosomal region; rather, proteasome clusters were found almost exclusively in the nucleus, with some also present in the tail. Further studies of capacitated sperm could help clarify what role sperm proteasomes may have in the degradation of the zona pellucida, as previously proposed^15,70^. A recent report proposed that the sperm 20S proteasome binds oocyte-derived 19S regulatory particles to degrade fetuin B and prevent polyspermy^65^. However, 20S proteasomes have been shown to be present in oocytes^65,71–73^ and, according to our structural data, α4s* does not promote preferential binding to 19S. It is, however, plausible that α4s* interactors in the cytoplasm of the zygote drive proteasomal activity after fertilization. Future structural work of zygote proteasomes is needed to clarify these pathways.

Lastly, as protamine disulfide bonds are reduced in mature oocytes^74,75^, 20S sperm proteasomes from lacunae may be responsible for protamine degradation in the zygote, driving decondensation of the paternal genome^65,71–73^.

Overall, our integrated structural and cellular analyses reveal the in situ structure, organization and compositional variance of the nuclear human sperm proteasome and uncover sperm-specific features. Our high-resolution structures provide molecular insight into substrate processing, as well as the roles of PA200 and the testis-specific subunit α4s, which we find to be a specific splice variant in human sperm cells. Together, our study provides a structural framework for future studies on proteasome function and regulation during sperm-cell differentiation and early embryonic development.

## Methods

### Human sperm and testis tissue samples

Human sperm cells were obtained from the European Sperm Bank (ESB). The samples were preprocessed by the ESB using density gradient centrifugation before cryopreservation. Donors were also tested by the ESB according to national requirements and the EU Commission directive 2006/17/EC. Donor consent to use the samples for research purposes was obtained by the ESB. Human sperm cells were checked for motility using an optical microscope and used immediately after thawing. Sperm cells from over 25 donors were used in this study.

Formalin-fixed paraffin-embedded FFPE testis sections (5 µm thick) from >10 donors, with no known history of infertility, 18–55 years of age, were obtained from the Human Research Tissue Bank (Cambridge University Hospitals), the Imperial College Healthcare Tissue Bank (Department of Surgery and Cancer) or commercially (AMSBIO, HP-401 and BP-401). All experimental procedures involving human tissue were performed under Research Ethics Committee approval (24/WM/0226, Integrated Research Application System (IRAS) project no. 340922) and the Imperial College Healthcare Tissue Biobank (ICHTB; approval no. 22/WA/0214, Human Tissue Authority license no. 12275).

### Mouse sperm and tissue

Sperm cells from C57BL/6 JAX mice were obtained from the Laboratory of Molecular Biology (LMB) Ares facility. After thawing, sperm cells checked for motility and used immediately in downstream experiments. Mouse (C57BL/6) testis FFPE sections (5 µm thick) were purchased from AMSBIO. All animal procedures were performed in accordance with institutional guidelines and the UK Animals (Scientific Procedures) Act 1986.

### Sample preparation and cryo-FIB milling

Quantifoil R1/4 Au200 EM grids (Quantifoil Micro Tools) were glow-discharged at 30 mA for 45 s from both sides in the Edwards S150B glow discharger. A total of 1 × 10^5^–5 × 10^5^ sperm cells were applied on the grid, blotted for 9–12 s and vitrified in liquid ethane using either a Leica GP2 (Leica) or a custom-made manual plunger. The vitrified cells were thinned using a Scios cryo-FIB/SEM (Thermo Fisher Scientific), an Aquilos II cryo-FIB/SEM instrument (Thermo Fisher Scientific) or a Crossbeam 550 cryo-FIB/SEM instrument (Zeiss) as previously described^69,78–80^. Briefly, inorganic platinum was sputtered on the grid in a Quorum chamber (Quorum Technologies) for the Scios and the Crossbeam 550 or in the main chamber for the Aquilos II. Next, the cells were coated with organometallic platinum using a gas injection system. The lamellae were milled at a milling angle of 7°–9° relative to the grid’s plane with a stepwise staircase pattern at currents of 1 nA, 0.5 nA, 0.3 nA, 0.1 nA and 30 pA for subsequent milling steps until a thickness of 150–200 nm.

### Cryo-ET data collection, tomogram reconstruction and subtomogram averaging

Cryo-ET data were collected on either a 300-kV Titan Krios G3i transmission EM instrument (Thermo Fisher Scientific) equipped with a Bioquantum energy filter (Gatan) and a K3 direct electron detector (Gatan) or a 300-kV Titan Krios G4 microscope equipped with a SelectrisX energy filter (EF) and Falcon 4i post-EF direct electron detector (Thermo Fisher Scientific). The tilt series were collected using a dose-symmetric scheme with a total fluence of 100 e^−^ per Å^2^ (3 e^−^ per Å^2^ per tilt) and defocus range of −2 to −5 µm using SerialEM PACE-tomo script^81^. A total of 270 tilt series were obtained and processed with Warp (version 1.0.9)^82^. The tilt series were aligned with AreTomo2 (ref. ^83^) and tomograms were reconstructed at ~10 Å per pixel through weighted backprojection and filtered to 60 Å for particle picking. Proteasome particles were picked using crYOLO^84^. A total of 46,831 particles were used for initial map generation and alignment with Dynamo^85^. RELION (version 3.1)^86^ was used for refining the initial poses, resulting in a 19.5-Å map. Masked 3D classification in RELION resulted in three classes (20S with 43,128 particles, 20S–PA200 with 2,430 particles and PA200–20S–PA200 with 245 particles) whose poses were refined with M (poses, image warp and stage angles)^87^ to obtain the final maps.

### Cryo-SEM tomography

Cryo-FIB/SEM volume imaging was performed with a Crossbeam 550 cryo-FIB/SEM instrument (Zeiss)^34^, equipped with a PP3010Z cryo stage and loading system (Quorum Technologies). A carpet of vitrified sperm cells on the grid was milled at a stage tilt of −4° with a current of 300 pA at 30 kV and a slice thickness of 16 nm. The pixel size for SEM imaging was 4.7 nm for human and 4.98 nm for mouse sperm, using an InLens detector at 2.3 kV and 50 pA with line averaging. Volumes were processed in Fiji as previously described^88^. Briefly, contrast-limited adaptive histogram equalization was used to enhance contrast^89^; then, images were aligned using the SIFT algorithm^90^. Horizontal and vertical curtain stripes were removed by stripe suppression using bandpass filtering. Nine human and mouse sperm cells were segmented manually using Imod^91^.

### Proteasome purification from human sperm cells

All the steps were performed at 4 °C. A total of 200 × 10^6^ IUI-ready human sperm cells (ESB) were centrifuged at 300*g* for 15 min. The pellet was resuspended in buffer L (25 mM HEPES pH 7.4, 10% (v/v) glycerol, 1 mM ATP, 1 mM DTT, 125 U per ml Benzonase nuclease (Merck) and 1 mg ml^−1^ DNase I (Merck)) and incubated for 30 min. The cells were sonicated for 5 min, homogenized and incubated for 30 min. Next, the homogenate was centrifuged at 20,000*g* for 10 min and the supernatant was concentrated using Amicon Ultra centrifugal filter (100-kDa molecular weight cutoff (MWCO; Merck). The concentrated sample was loaded on Superose 6 increase 3.2/300 (Cytiva) equilibrated in buffer A (25 mM HEPES pH 7.4, 10% (v/v) glycerol, 100 mM NaCl, 1 mM ATP, 1 mM DTT and 5 mM MgCl_2_). The eluted fractions were assayed for proteasome presence using immunoblotting and negative-stain EM (NS-EM). Selected fractions were concentrated using Amicon Ultra centrifugal filter (100-kDa MWCO) and loaded on Superose 6 increase 3.2/300 (Cytiva) equilibrated in buffer B (25 mM HEPES pH 7.4, 100 mM NaCl, 1 mM ATP, 1 mM DTT and 5 mM MgCl_2_). The fractions were assayed for proteasome presence using immunoblotting and NS-EM, concentrated and immediately used for single-particle cryo-EM sample preparation.

### Immunoblotting

The protein fractions were mixed at a 1:1 ratio with 1× NUPAGE LDS sample buffer (Invitrogen) supplemented with 50 mM DTT and separated on NUPAGE 4–12% Bis–Tris SDS–PAGE gels (Invitrogen). Subsequently, the samples were transferred onto PVDF membrane (Bio-Rad) using a Trans-Blot semidry transfer system (Bio-Rad) and immunoblotted using an iBind Flex system (Thermo Fisher Scientific) according to the manufacturer’s instructions. The primary antibody, 20S proteasome β7 (H-3) (sc-365725, Santa Cruz Biotechnology), was used at 1:1,000 dilution. The secondary antibody, goat anti-mouse IgG (horseradish peroxidase; ab97023), was used at 1:2,000 dilution. Immunoblots were imaged using Chemidoc MP device (Bio-Rad).

### NS-EM

To assess proteasome presence in the chromatographic fractions using NS-EM, EM copper grids (carbon films on 200-mesh copper grids, Agar Scientific) were glow-discharged in the Edwards S150B glow discharger for 30 s at 40 mA and 0.1 mbar. Next, a 3-µl protein sample was deposited on the glow-discharged grid and incubated for 30 s at room temperature, followed by three wash steps in double-distilled H_2_O and staining in 2% (w/v) uranyl acetate for 1 min at room temperature. The samples were screened using a 120-kV Tecnai Spirit transmission EM instrument (Thermo Fisher Scientific) equipped with an Orius charge-coupled device camera (Gatan) at ×21,000 magnification and 2.53 Å per pixel.

### Single-particle cryo-EM sample preparation

Quantifoil R1.2/1.3 300-mesh copper holey carbon grids were cleaned with chloroform, acetone and isopropanol as described previously^92^. A 2.1-nm-thick continuous carbon layer was evaporated on a mica sheet (Agar Scientific) using the Leica EM ACE600 carbon coater according to the manufacturer’s instructions. Next, the carbon layer was floated on water and transferred onto the cleaned grids. Subsequently, the continuous carbon-coated grids were glow-discharged in the Edwards S150B for 30 s at 40 mA and 0.1 mbar and placed in a Vitrobot Mark IV (Thermo Fisher Scientific) semiautomated plunger equilibrated at 4 °C and 100% relative humidity. Then, 3 μl of concentrated proteasome sample was applied to the grid, incubated for 30 s and blotted with Whatman no. 1 filter paper for 2 s at a blot force of −7. Finally, the grid was vitrified in liquid ethane maintained at 93 K using a cryostat device^93^ and stored in liquid nitrogen.

### Single-particle cryo-EM data collection

Cryo-EM data was collected on a 300-kV Titan Krios G4 microscope equipped with SelectrisX EF and Falcon 4i post-EF direct electron detector (Thermo Fisher Scientific) using aberration-free image shift. The energy filter slit was set at a width of 10 eV and the defocus varied between −1.0 µm and −2.0 µm. Zero-loss frames were collected at ×105,000 magnification and a corresponding calibrated pixel size of 0.926 Å per pixel in EER format with 40 e^−^ per Å^2^ total fluence over 3 s of exposure. Four datasets were acquired. Dataset 1 comprised 9,158 micrographs of the 20S proteasome fraction (Extended Data Fig. 4e). Datasets 2–4 comprised 9,832, 26,881 and 13,500 micrographs, respectively, of the 20S–PA200 proteasome fraction (Extended Data Fig. 4e).

### Single-particle cryo-EM data processing

Dataset 1 was preprocessed in RELION (version 4.0.1)^94^. Datasets 2–4 were preprocessed in RELION (version 5.0.1)^95^. For each dataset, 918 hardware EER frames were grouped into 45 fractions, resulting in a dose of 0.87 e^−^ per Å^2^ per fraction and motion-corrected using MotionCor2 in patch mode^96^. The contrast transfer function (CTF) parameters were estimated with CTFFIND (version 4.1)^97^. A total of micrographs of various defoci from dataset 1 were manually picked and used to train the neural network of crYOLO (version 1.9.7)^84^. The trained model was used for automated particle picking on each entire dataset. The particle number and map resolution for all processing steps are shown in Extended Data Fig. 4f. The particles were extracted in RELION, imported into cryoSPARC (version 4.4.1)^98^ and two-dimensionally (2D) classified into 100 classes to remove false-positive picks. The selected particles were used to generate five (dataset 1) or six (datasets 2–4) initial models. In each dataset, one of the maps corresponded to the intact, isotropic 20S reconstruction, while other maps consisted of defective and overrepresented particles. Heterogeneous refinement was used to assign particles corresponding to each map. The particles corresponding to the best class were selected and reimported into RELION using pyem^99^. Datasets 3 and 4 were combined into a single star file with two optics groups. Each particle subset was 3D autorefined followed by two iterations of per-particle CTF estimation, aberration corrections^86^ and 3D autorefinement. The particles from datasets 3 and 4 were split into separate star files and subjected to Bayesian polishing^100^. The polished particles were imported into cryoSPARC (version 4.4.1). Particles from dataset 1 were processed separately, while datasets 2–4 were combined with optic group separation. The sets were homogeneously refined with *C*_2_ symmetry, optimization of per-particle defocus, per-group CTF parameters and correction of the Ewald sphere with negative curvature^101^. Next, the particles of datasets 2–4 were split into 69 groups according to the beam-image shift parameters to refine the beam tilt, trefoil and spherical aberration parameters for each group separately, followed by per-particle CTF estimation and homogeneous refinement with parameters corresponding to the ‘homogeneous refinement 1’ step. The particles of datasets 2–4 were subjected to 3D classification with 15 classes and a mask encompassing the region outside the 20S CP. Class 1 corresponded to the 20S CP bound to the PA200 activator (6,748 particles), while class 2 represented the 20S proteasome with the AAA-ATPase part of the 19S activator (4,207 particles). Each of the two classes was subjected to nonuniform reconstruction with default parameters, resulting in maps of 2.9-Å and 3.3-Å global resolution at a Fourier shell correlation (FSC) of 0.143 for 20S–PA200 and 20S–AAA-ATPase, respectively. The remaining particles from datasets 2–4, as well as the particles from dataset 1 homogeneous refinement 1, were reimported in RELION using pyem. A RELION script, eer_trajectory_handler.py, was used to change the rendering mode to the 8 K grid and group each of the eight EER frames into a single fraction. Each particle set was subjected to Bayesian polishing with particle reextraction at a super-resolution pixel size of 0.668 Å per pixel and reimported into cryoSPARC. The particles from each dataset were split into 69 groups according to the beam-image shift parameters. Following homogeneous refinement, with *C*_2_ symmetry, minimization over per-particle scale, optimization of per-particle defocus and per-group CTF parameters (tilt, trefoil, spherical aberration, tetrafoil and anisotropic magnification), as well as Ewald sphere correction with a negative curvature, the particles were 3D classified into ten classes at 5-Å filtering resolution, with initial class similarity of 0.8 and the ‘autotune initial class similarity’ option turned on. This revealed a single main class of 222,000 particles that was used for nonuniform refinement with *C*_2_ symmetry and Ewald sphere correction. The refinement resulted in a 1.85-Å-resolution map at the FSC threshold of 0.143.

To obtain a complete 26S map, the proteasome particles from datasets 2–4 were reimported into RELION using pyem and subjected to Bayesian polishing with reextraction at the extended 600-pixel box size to incorporate the entire 26S proteasome. The particles were reimported into cryoSPARC, 2D classified to remove particles extracted near the edges of the micrographs and homogeneously refined with *C*_2_ symmetry. The particle number and map resolution for all processing steps are shown in Extended Data Fig. 8a. The particles were symmetry-expanded and 3D classified into ten classes with a mask encompassing the region outside of the CP. One of the classes corresponded to the 20S with AAA-ATPase part of the 19S activator. It was further 3D classified into six classes with a large mask encompassing the entire 19S activator. The best class was homogeneously reconstructed, revealing a 4.4-Å map of the entire 26S with the 19S activator at low resolution (>17 Å; Extended Data Fig. 8b).

### Model building

The asymmetric unit of the somatic 20S proteasome structure (PDB 6RGQ)^45^ was rigid-body fitted into the 1.85-Å map of the sperm 20S CP in UCSF ChimeraX (version 1.9)^102^. The α4s initial model was generated with AlphaFold2 (ref. ^103^) using the sequence of the alternatively spliced variant of α4s (UniProt Q8TAA3-5). The α4 subunit was replaced with α4s initial model in Coot (version 0.9)^104^ and refined in real space with PHENIX (version 1.21.1)^105^. Water molecules surrounding the asymmetric unit were added to the map and validated using the ‘check/delete waters’ tool in Coot. Following several iterations of manual rebuilding in Coot and real-space refinement in PHENIX (version 2.0-5884), the noncrystallographic symmetry operators were applied to generate a structure with *C*_2_ symmetry. Lastly, the chains were renamed to match the 20S–PA200 structure in Coot. To confirm the presence of the alternatively spliced α4s variant (UniProt Q8TAA3-5), referred as α4s*, rather than the canonical one (UniProt Q8TAA3-1, referred to as α4s FL), the asymmetric unit of the somatic 20S proteasome structure (PDB 6RGQ)^45^ was rigid-body fitted into the 1.85-Å map of the sperm 20S CP in UCSF ChimeraX (version 1.9)^102^. The α4 subunit was replaced with α4s AlphaFold2 model (AF-Q8TAA3-F1) in Coot (version 0.9)^104^, manually rebuilt and real-space-refined in PHENIX (version 1.21.1)^105^. To generate the 20S–PA200 structure, the somatic 20S–PA200 coordinates (PDB 6KWY)^39,45^ were rigid-body fitted into the 2.9-Å map of the sperm 20S–PA200 proteasome with UCSF Chimera (version 1.9). The α4 subunit was replaced with α4s* coordinates from sperm 20S CP with Coot (version 0.9), followed by multiple iterations of real-space refinement in PHENIX (version 2.0-5884) and manual rebuilding in Coot. The models were validated with MolProbity^106^. The molecular graphics and density map visualizations were generated in UCSF ChimeraX (version 1.9) under a noncommercial software license agreement. Final figure assembly was conducted in Adobe Illustrator under an institutional subscription.

### Immunofluorescence

Human and mouse sperm cells were seeded on no. 1.5 glass coverslips prefunctionalized with poly(L-lysine) and allowed to dry at room temperature for 5–10 min. Cells were then fixed in 4% (w/v) PFA in PBS for 10 min at room temperature, followed by three washes in PBS. Samples were quenched for 15 min at room temperature using 50 mM NH_4_Cl in PBS and then washed three times in PBS. Following this, cells incubated in 3% (w/v) BSA and 0.2% (v/v) Triton X-100 in PBS for 1 h at room temperature. Primary antibody incubations were performed for 2 h at room temperature or overnight at 4 °C and antibodies were diluted in 3% (w/v) BSA and 0.2% (v/v) Triton X-100 in PBS. Cells were then washed with 0.5% (w/v) BSA and 0.05% (v/v) Triton X-100 in PBS, followed by incubation with fluorescently labeled secondary antibodies for 1 h at room temperature. Secondary antibodies were also diluted in 3% (w/v) BSA, 0.2% (v/v) Triton X-100 in PBS. Cells were then washed three times in 0.5% (w/v) BSA and 0.05% (v/v) Triton X-100 in PBS, followed by PBS. For samples where DNA was stained, cells were incubated for 15 min at room temperature with Hoechst 33342 (Invitrogen) or SiR–DNA (SpyroChrome) diluted in PBS, followed by three washes in PBS. Coverslips were then washed once in distilled H_2_O and mounted in Prolong Gold mounting medium for SIM imaging (Thermo Fisher Scientific).

For STORM experiments, sperm cells in ibidi eight-well chamber slides prefunctionalized with poly(L-lysine) were allowed to dry. Cells were then prepared for imaging using a standard immunofluorescence protocol (described above) or using the ONI discovery kit for dSTORM (ONI, 900-00031), with only PFA used as a fixative (provided with kit), following the manufacturer’s protocol.

Primary antibodies used for immunofluorescence were as follows: mouse anti-20S Proteasome β7 (H-3) (Santa Cruz, sc-365725, 1:50), rabbit anti-PSMD2 (Invitrogen, PA5-27663, 1:100), rabbit PSME4/PA200 (Sigma, HPA060922, 1:100), rabbit anti-PSMC1 (abcam, ab223765, 1:100), mouse anti-PSMA8 (143–242) clone 5A8 (Abnova, H00143471-M04A, 1:100), rabbit anti-PSMA8 (Proteintech, 14022-1-AP, 1:100), mouse anti-PSMA3 (Enzo, BML-PW8110, 1:100), rabbit anti-PRM1 (Invitrogen, PA5-63074, 1:100) and acetylated tubulin-Atto640 (gift from Emmanuel Derivery). Secondary antibodies were as follows: donkey anti-mouse-Alexa488 (abcam, ab181289, 1:500), donkey anti-mouse-Alexa647 (abcam, ab150111, 1:500), donkey anti-rabbit-Alexa488 (abcam, 181346, 1:500), donkey anti-rabbit-Alexa647 (abcam, ab181347, 1:500), anti-rabbit F(ab′)2 secondary antibody labeled with AZ647 (ONI, 800-00090, 1:100) and anti-mouse F(ab′)2 secondary antibody labeled with CF583R (ONI, 800-00086, 1:100).

### STORM and cluster analysis

Immunofluorescently labeled cells were imaged using a Nanoimager (ONI) system, equipped with a ×100 (numerical aperture (NA):1.49) total internal fluorescence reflection oil-immersion objective and a Hamamatsu Orca Flash 4.0 v3 camera. STORM measurements were performed under highly inclined and laminated optical sheet (HILO) illumination, using 488-nm, 561-nm and 640-nm laser lines, with laser power 60–150 mW, for 15,000 frames (50,000 for DNA) and exposure time of 30 ms. ONI Bcubed buffer was used for imaging (ONI, 900-00031). For two-color STORM measurements, a calibration procedure with TetraSpeck beads (Invitrogen) was used for channel alignment, according to manufacturer’s guidance. STORM data were processed using CODI software (ONI), including drift correction and HDBSCAN analysis. For HDBSCAN^35^ analysis, a minimum cluster size was set at 15 molecules. Technical specifications for this and other optical microscopes used in this study can be found in Supplementary Table 1.

### SIM

A Carl Zeiss Elyra S.1 SR-SIM super-resolution inverted microscope (Carl Zeiss) was used for SIM. A ×63 (NA: 1.46) Plan-apochromat oil-immersion objective was used for data acquisition, with 405-nm, 488-nm, 561-nm and 647-nm laser lines and a PCO.edge complementary metal–oxide–semiconductor (CMOS) camera. Further microscope specifications can be found in Supplementary Table 1. Then, *z* stacks (0.091 µm) were collected with three or five rotations. For two-color imaging, channel alignment was performed with mounted TetraSpeck fluorescent beads (Invitrogen), using the affine method. Channel alignment, deconvolution and SR-SIM reconstruction were performed using Zen Black 2012 SP5 FP3 (Carl Zeiss).

### Immunohistochemistry

Premounted FFPE testis sections (5 µm thick) from human healthy donors or C57BL/6 mice (5 µm thick) were deparaffinized and rehydrated. Tissue sections, submerged in 10 mM sodium citrate and 0.05% Tween-20 (pH 6), were then subjected to heat-induced antigen retrieval. Following this, tissue slides were incubated in 0.1% (v/v) Triton X-100 in TBS for 15 min at room temperature. Blocking was then performed for 30 min at room temperature in 1% (w/v) BSA, 10% (v/v) donkey serum in TBS. Primary antibody incubation was performed overnight at 4 °C in a humidified environment. The above-listed antibodies were diluted in 1% (w/v) BSA in TBS. The next day, tissue sections were washed three times in at least 60 ml of 0.05% (v/v) Triton X-100 in TBS, for 10 min at room temperature, with rocking. Tissue sections were then incubated for 1 h at room temperature in a humidified environment, with fluorescently labeled secondary antibodies diluted in 1% (w/v) BSA in TBS. For DNA staining, tissue slides were incubated with Hoechst 33342 (Thermo Fisher Scientific) in 0.05% (v/v) Triton X-100 in TBS for 15 min at room temperature with rocking. This was followed by three 10-min washes in 0.05% (v/v) Triton X-100 in TBS at room temperature, a TBS wash and a final wash in distilled H_2_O. Samples were mounted using no. 1.5 coverslips and Prolong Gold mounting medium (Thermo Fisher Scientific).

### Confocal microscopy

Animal and human tissue slides were imaged using an Andor benchtop confocal BC43 (Oxford Instruments), equipped with a scientific CMOS detector Andor Zyla 4.2 Plus. For data collection, a ×60 (NA: 1.42) oil-immersion objective was used, together with 405-nm, 488-nm, 561-nm and 638-nm laser lines. Further details can be found in Supplementary Table 1. Andor Fusion (version 2.7.0; Oxford Instruments) software was used for image acquisition. Image analysis was performed using Fiji (ImageJ).

### Ethics declarations

Human mature sperm cells were purchased from the ESB (https://www.europeanspermbank.com/en). The samples were previously processed, tested by the bank according to the requirement of the national competent authorities, Good Manufacturing Practice and World Health Organization standards and EU directives 2006/86/EC, 2006/17/EC, 2004/23/EC and 2015/565. Sperm cells were screened for morphology, genetic diseases, blood-borne viruses and motility. Consent to use the samples for research purposes was given to the ESB. Human tissue samples used in this research project were obtained from the ICHTB. ICHTB is supported by the National Institute for Health Research Biomedical Research Center based at Imperial College Healthcare National Health Service Trust and Imperial College London. ICHTB is approved by Wales REC3 to release human material for research (22/WA/0214) and the samples for this project (R24008). This study was also conducted under Research Ethics Committee approval (24/PR/1237; IRAS project no. 340922).

### Reporting summary

Further information on research design is available in the Nature Portfolio Reporting Summary linked to this article.

## Online content

Any methods, additional references, Nature Portfolio reporting summaries, source data, extended data, supplementary information, acknowledgements, peer review information; details of author contributions and competing interests; and statements of data and code availability are available at https://doi.org/10.1038/s41594-026-01870-z.

## Supplementary information

Reporting Summary

Peer Review File

Supplementary Video 1 The molecular architecture of a large proteasome-rich nuclear lacuna. Summarizing video displaying the three proteasome maps from subtomogram averaging mapped back in a denoised cryo-tomogram of the sperm nucleus.

Supplementary Video 2 Volumetric cryo-EM of human sperm cells shows nuclear lacunae. Video displaying cryo slice-and-view imaging of several sperm cells with nuclear lacunae segmented in green in the central cell. Scale bar, 1 µm.

Supplementary Table 1 Light microscopy reporting table.

## Source data

Source Data Fig. 2 Excel file with lacuna size and clustering length.

Source Data Extended Data Fig. 1 Excel table with FSC measurements for STA maps.

Source Data Extended Data Fig. 4 Excel table with SEC elution absorbance values.

Source Data Extended Data Fig. 4 Full blots for Extended Data Fig. 4.

Source Data Extended Data Fig. 5 Excel table with FSC measurements for SPA 20S and PA200–20S maps.

Source Data Extended Data Fig. 8 Excel table with FSC measurements for SPA 26S map.

Source Data Extended Data Fig. 9 Excel table with nuclear/cytoplasmic fluorescent intensity ratios.

Source Data Extended Data Fig. 10 Excel table with nuclear/cytoplasmic fluorescent intensity.

## Data Availability

Subtomogram averages of the 20S, PA200 and PA200–20S–PA200 structures were deposited to the EM Data Bank with accession codes EMD-55396, EMD-55397 and EMD-55398, respectively. Single-particle cryo-EM maps for 20S, 20S–PA200, 20S–AAA-ATPase and 26S were deposited to the EM Data Bank with accession codes EMD-56072, EMD-56074, EMD-56075 and EMD-58412, respectively. The atomic coordinates of 20S and 20S–PA200 were deposited to the Protein Data Bank with accession codes PDB 9TMN and PDB 9TMS, respectively. Source data are provided with this paper.
