# Supplementary material for: Molecular architecture and spatial organization of proteasomes in the human sperm nucleus
Source: Nat Struct Mol Biol. 2026 Sep 1;33(9):1367–80. doi: 10.1038/s41594-026-01870-z (PMC13561864; doi:10.1038/s41594-026-01870-z)
Supplement: Supplementary file 1 [file 41594_2026_1870_MOESM1_ESM.pdf]

nature portfolio

Corresponding author(s): Matteo Allegretti, Alia dos Santos, Tom Dendooven

Last updated by author(s): Jul 23, 2026

Reporting Summary

Nature Portfolio wishes to improve the reproducibility of the work that we publish. This form provides structure for consistency and transparency in reporting. For further information on Nature Portfolio policies, see our Editorial Policies and the Editorial Policy Checklist.

Statistics

For all statistical analyses, confirm that the following items are present in the figure legend, table legend, main text, or Methods section.

n/a	Confirmed	
☐	☒	The exact sample size (n) for each experimental group/condition, given as a discrete number and unit of measurement
☐	☒	A statement on whether measurements were taken from distinct samples or whether the same sample was measured repeatedly
☐	☒	The statistical test(s) used AND whether they are one- or two-sided<br>*Only common tests should be described solely by name; describe more complex techniques in the Methods section.*
☒	☐	A description of all covariates tested
☐	☒	A description of any assumptions or corrections, such as tests of normality and adjustment for multiple comparisons
☐	☒	A full description of the statistical parameters including central tendency (e.g. means) or other basic estimates (e.g. regression coefficient) AND variation (e.g. standard deviation) or associated estimates of uncertainty (e.g. confidence intervals)
☐	☒	For null hypothesis testing, the test statistic (e.g. F, t, r) with confidence intervals, effect sizes, degrees of freedom and P value noted<br>*Give P values as exact values whenever suitable.*
☒	☐	For Bayesian analysis, information on the choice of priors and Markov chain Monte Carlo settings
☒	☐	For hierarchical and complex designs, identification of the appropriate level for tests and full reporting of outcomes
☒	☐	Estimates of effect sizes (e.g. Cohen's d, Pearson's r), indicating how they were calculated

Our web collection on statistics for biologists contains articles on many of the points above.

Software and code

Policy information about availability of computer code

Data collection	Serial EM 4.2.0 beta PACE tomo, EPU 3.13, Andor Fusion 2.7.0 (Oxford Instruments), ZEISS Zen Black 2012 SP5 FP3, NimOS version 1.19.15, SmartSEM/SmartFIB (Zeiss)
Data analysis	Warp 1.0.9 , AreTomo2, CrYOLO 1.9.7, RELION 3.1, RELION 4.0.1, RELION 5.0.1, M, FiJi ImageJ 2.3.0, Dynamo 1.1.532, cryoSPARC v4.4.1, USCF ChimeraX 1.9, Coot 0.9, PHENIX 1.21.1, PHENIX 2.0-5884, CODI software (ONI), ZEISS Zen Black 2012 SP5 FP3

For manuscripts utilizing custom algorithms or software that are central to the research but not yet described in published literature, software must be made available to editors and reviewers. We strongly encourage code deposition in a community repository (e.g. GitHub). See the Nature Portfolio guidelines for submitting code & software for further information.

Data

Policy information about availability of data

All manuscripts must include a data availability statement. This statement should provide the following information, where applicable:

- Accession codes, unique identifiers, or web links for publicly available datasets
- A description of any restrictions on data availability
- For clinical datasets or third party data, please ensure that the statement adheres to our policy

Subtomogram averages of the 20S, PA200 and PA200-20S-PA200 were deposited in the Electron Microscopy Data Bank (EMDB) with accession codes EMD-55396, EMD-55397, and EMD-55398, respectively. Single particle cryo-EM maps for 20S, 20S-PA200, and 26S were deposited in EMDB with accession codes EMD-56072,

EMD-56074, and EMD-56075, respectively. The atomic coordinates of 20S and 20S-PA200 were deposited in the Protein Data Bank (PDB) with accession codes PDB ID: 9TMN and 9TMS, respectively.

Research involving human participants, their data, or biological material

Policy information about studies with human participants or human data. See also policy information about sex, gender (identity/presentation), and sexual orientation and race, ethnicity and racism.

Reporting on sex and gender	Male
Reporting on race, ethnicity, or other socially relevant groupings	N/A
Population characteristics	N/A
Recruitment	N/A
Ethics oversight	Human mature sperm cells were purchased from the European Sperm Bank (https://www.europeanspermbank.com/en). The samples had been previously processed, tested by the bank according to the requirement of the national competent authorities, GMP and WHO standards, as well as EU directives: 2006/86/EC, 2006/17/EC, 2004/23/EC, 2015/565. Sperm cells were screened for morphology, genetic diseases, blood-borne viruses and motility. Consent to use the samples for research purposes was given to the European Sperm Bank. All the human tissue experimental procedures were performed under REC approval (REC Ref. 24/WM/0226, IRAS Project ID: 340922) and the Imperial College Healthcare Tissue bank (Research Ethics Committee approval numbers: 22/WA/0214; ICHTB HTA license: 12275).

Note that full information on the approval of the study protocol must also be provided in the manuscript.

Field-specific reporting

Please select the one below that is the best fit for your research. If you are not sure, read the appropriate sections before making your selection.

☒ Life sciences ☐ Behavioural & social sciences ☐ Ecological, evolutionary & environmental sciences

For a reference copy of the document with all sections, see nature.com/documents/nr-reporting-summary-flat.pdf

Life sciences study design

All studies must disclose on these points even when the disclosure is negative.

Sample size	Human tissue: over 10 human donors<br>Human Sperm cells: 25 donors
Data exclusions	N/A
Replication	Sperm cells were obtained from 25 different donors. Multiple technical replicates were performed for both cryo-EM and light microscopy data. Human tissue samples from >10 donors were used for immunohistochemistry data collection, with multiple technical replicates.
Randomization	N/A
Blinding	N/A

Reporting for specific materials, systems and methods

We require information from authors about some types of materials, experimental systems and methods used in many studies. Here, indicate whether each material, system or method listed is relevant to your study. If you are not sure if a list item applies to your research, read the appropriate section before selecting a response.

Materials & experimental systems

n/a	Involved in the study
☐	☒ Antibodies
☒	☐ Eukaryotic cell lines
☒	☐ Palaeontology and archaeology
☒	☐ Animals and other organisms
☒	☐ Clinical data
☒	☐ Dual use research of concern
☒	☐ Plants

Methods

n/a	Involved in the study
☒	☐ ChIP-seq
☒	☐ Flow cytometry
☒	☐ MRI-based neuroimaging

Antibodies

Antibodies used

mouse anti-proteasome β7, Santa Cruz Biotechnology (sc-365725, 1:50 dil)
Rabbit anti-PSMA8, Proteintech (14022-1-AP, 1:100 dil)
mouse anti-PSMA8 (143-242) clone 5A8, Abnova (H00143471-M04A, 1:100 dil)
rabbit anti-PSMC1, Abcam (ab223765, 1:100 dil)
rabbit anti-PSME4/PA200, Sigma (HPA060922, 1:100 dil)
rabbit anti-PSMA7, Abcam (ab133502, 1:100 dil)
mouse anti-PSMA7 (OTI8F9), InsightBio/OriGene (TA500694, 1:100 dil)
mouse anti-PSMA3, ENZO (BML-PW8110, 1:100 dil)
rabbit anti-PSMD2 (Invitrogen, PA5-27663, 1:100 dil)
rabbit anti-PRM1 (Invitrogen, PA5-63074, dil. 1:100)
acetylated alpha-tubulin antibody (gift from Emmanuel Derivery, MRC-Laboratory of Molecular Biology, Cambridge, 1:250 dil)
Donkey F(ab')2 Anti-Mouse IgG H&L (Alexa Fluor 488), Abcam (ab181289, 1:500 dil)
Donkey Anti-Mouse IgG H&L (Alexa Fluor 647) preadsorbed, Abcam (ab150111, 1:500 dil)
Donkey F(ab')2 Anti-Rabbit IgG H&L (Alexa Fluor 488) preadsorbed, Abcam (ab181346, 1:500 dil)
Donkey F(ab')2 Anti-Rabbit IgG H&L (Alexa Fluor 647) preadsorbed, Abcam (ab181347, 1:500 dil)
Goat Anti-Mouse IgG-Atto 488, Merck (62197, 1:500 dil)
Goat Anti-Rabbit IgG-Atto 488, Merck (18772, 1:500 dil)
anti-rabbit F(ab')2 secondary antibody labelled with AZ647 (ONI, # 800-00090, 1:100dil)
anti-mouse F(ab')2 secondary antibody labelled with CF583R (ONI, cat. # 800-00086, 1:100 dil)

Validation

mouse anti-proteasome β7, Santa Cruz Biotechnology (sc-365725) is a monoclonal primary antibody validated by the manufacturer and recommended for use with western blotting, immunofluorescence, immunoprecipitation.
Rabbit anti-PSMA8, Proteintech (14022-1-AP) is a polyclonal primary antibody, validated by the manufacturer for use in immunohistochemistry, western blotting, immunofluorescence
mouse anti-PSMA8 clone 5A8, Abnova (H00143471-M04A) is a monoclonal primary antibody raised against a partial recombinant PSMA8, validated by the manufacturer and recommended for use in western blotting.
rabbit anti-PSMC1, Abcam (ab223765) is a polyclonal primary antibody validated by the manufacturer and recommended for use in immunohistochemistry, western blotting and immunofluorescence
rabbit anti-PSME4/PA200, Sigma (HPA060922) is a polyclonal primary antibody validated by the manufacturer and recommended for use in immunofluorescence and western blotting.
rabbit anti-PSMA7, Abcam (ab133502) is a monoclonal primary antibody, validated by the manufacturer and recommended for use in immunohistochemistry and western blotting.
mouse anti-PSMA7 (OTI8F9), InsightBio/OriGene (TA500694) is a monoclonal antibody validated by the manufacturer and recommended for use in immunofluorescence and western blotting.
mouse anti-PSMA3, ENZO (BML-PW8110) is a primary antibody validated by the manufacturer for use in immunohistochemistry and western blotting.
rabbit anti-PSMD2 (Invitrogen, PA5-27663) is a polyclonal primary antibody validated by the manufacturer and recommended for use in immunofluorescence.
rabbit anti-PRM1 (Invitrogen, PA5-63074) is a polyclonal primary antibody validated by the manufacturer for use in Immunohistochemistry and immunocytochemistry.
acetylated alpha-tubulin antibody clone C3B9 (gift from Emmanuel Derivery, MRC-Laboratory of Molecular Biology, Cambridge) from HPA cultures is described and characterised in Derivery et al., Nature 2015 (PMID: 26659188)
Donkey F(ab')2 Anti-Mouse IgG H&L (Alexa Fluor 488), Abcam (ab181289) is a polyclonal secondary antibody validated by the manufacturer and recommended for immunohistochemistry, immunofluorescence, ELISA, flow cytometry
Donkey Anti-Mouse IgG H&L (Alexa Fluor 647) preadsorbed, Abcam (ab150111) is a polyclonal secondary antibody validated by the manufacturer and recommended for immunohistochemistry, immunofluorescence, ELISA, flow cytometry
Donkey F(ab')2 Anti-Rabbit IgG H&L (Alexa Fluor 488) preadsorbed, Abcam (ab181346) is a polyclonal secondary antibody validated by the manufacturer and recommended for immunohistochemistry, immunofluorescence, ELISA, flow cytometry
Donkey F(ab')2 Anti-Rabbit IgG H&L (Alexa Fluor 647) preadsorbed, Abcam (ab181347) is a polyclonal secondary antibody validated by the manufacturer and recommended for immunohistochemistry, immunofluorescence, ELISA, flow cytometry
Goat Anti-Mouse IgG-Atto 488, Merck (62197) is a polyclonal secondary antibody validated by the manufacturer and recommended for use in immunofluorescence
Goat Anti-Rabbit IgG-Atto 488, Merck (18772) is a polyclonal secondary antibody validated by the manufacturer and recommended for use in immunofluorescence and immunohistochemistry
anti-rabbit F(ab')2 secondary antibody labelled with AZ647 (ONI, # 800-00090) and anti-mouse F(ab')2 secondary antibody labelled with CF583R (ONI, cat. # 800-00086) are secondary antibodies validated by the manufacturer for use in STORM measurements

Plants

Seed stocks	N/A
Novel plant genotypes	N/A
Authentication	N/A
