# Supplementary material for: Molecular architecture and spatial organization of proteasomes in the human sperm nucleus
Source: Nat Struct Mol Biol. 2026 Sep 1;33(9):1367–80. doi: 10.1038/s41594-026-01870-z (PMC13561864; doi:10.1038/s41594-026-01870-z)
Supplement: Supplementary file 2 [file 41594_2026_1870_MOESM2_ESM.pdf]

nature portfolio

Peer Review File

Molecular architecture and spatial organisation of proteasomes in the human sperm nucleus

Corresponding Author: Dr Matteo Allegretti

Version 0:

Decision Letter:

16th Dec 2025

Dear Dr. Allegretti,

Thank you again for submitting your manuscript "Molecular architecture and spatial organisation of proteasomes in the human sperm nucleus". I apologise for the delay in sharing our decision. We have decided to send your manuscript for peer review, but several points require your attention before we can proceed with peer review and start trying to recruit experts (given the the winter holidays will soon start, we will only start recruiting referees at the beggining of January).

I am re-opening the manuscript submission system for you to resubmit your manuscript with all associated files needed for peer review directly, within 2-3 business days if possible. If you require longer than that given that the winter holidays are almost here, please let me know and it would be more than fine to take the necessary time. Please follow the link at the bottom of this email to upload the documents listed below. If you have any issues, please reach out to us before completing the submission.

1. STRUCTURAL DATA:
We require official wwPDB validation reports for newly described atomic structures per journal policy. We also request that authors provide cryo-EM maps, half-maps and models, as well as maps and models obtained from subtomogram averaging, to help the reviewers in assessing the work. We very strongly recommend the use of figshare in our system, which allows for provision of anonymous access links for the referees (https://www.springernature.com/gp/authors/research-data/figshare-integration). Alternatively, please upload .zip folders directly with the submission.

Manuscripts reporting new structures should contain a table summarizing structural and refinement statistics. Please include these tables for both new cryo-EM and cryo-ET data (https://www.nature.com/documents/nr-tables-cryo-em.doc and modifying this file for ET). For cryo-EM structures, a representative micrograph showing individual particles should be provided in the figures alongside a processing workflow (please see our Editorial for more information: https://www.nature.com/articles/s41594-025-01567-9 and here for an example: https://www.nature.com/articles/s41594-025-01586-6/figures/7).

2. REPORTING SUMMARY:
We want to ensure that the methods and statistics reporting in our papers are of the highest quality. To that end, we ask authors to fill out a Reporting Summary that collects information on experimental design and reagents. If your paper includes ChIP-seq or flow cytometry data, we ask that you take special care to complete those sections of the Reporting Summary as this data will aid greatly in the review of your manuscript.

Reporting Summary:
https://www.nature.com/documents/nr-reporting-summary.pdf

3- PROTEOMICS AND SEQUENCING:
Please provide accession numbers and reviewer tokens to access sequencing or proteomics datasets if any unpublished datasets are part of your study. Please add this information to your manuscript file. Proteomics data should be deposited to any ProteomeXchange member repository (http://www.proteomexchange.org/) and DNA sequencing, RNA sequencing, genome assembly data should be deposited to any INSDC member repository (https://www.insdc.org/) or Genome Sequence Archive.

4- CODE, MD SIMULATIONS, AND MACHINE LEARNING REPORTING:

Please provide the code used to analyse the data if newly developed (unpublished) code was used in the work. For the reviewers to evaluate the work adequately, they must be able to test the software/review the code themselves. If you have not yet provided the software, we therefore request that you provide a single compressed zip file containing the software with a readme.txt file or other user manual containing complete instructions for installing and running the software. If appropriate, please provide example data and expected output. Sufficient material should be provided for referees to directly test the performance of the software/algorithm. Alternatively, you can upload the code to GitHub and provide the link directly in the manuscript.

Please fill out and upload the code and software submission checklist that will be made available to editors and reviewers. This form is a dynamic ‘smart pdf’ and must therefore be downloaded and completed in Adobe Reader, instead of opening it in a web browser.
https://www.nature.com/documents/nr-software-policy.pdf

If molecular dynamics (MD) simulations were performed, please refer to this Editorial and provide a completed MD simulations checklist with your manuscript: https://www.nature.com/articles/s42003-023-04653-0

Lastly, if it applies, please complete the completed machine learning checklist with your submission and upload it for review: https://www.nature.com/documents/machine-learning-checklist.pdf

Please use the link below to submit the files. **Please also remember to move forward all other files associated with this version of the paper.**

Link Redacted

Note: This URL links to your confidential home page and associated information about manuscripts you may have submitted, or that you are reviewing for us. If you wish to forward this email to co-authors, please delete the link to your homepage.

We look forward to seeing the revised manuscript and thank you for the opportunity to review your work. Please note that as we are entering the winter holiday period during which recruiting reviewers is very difficult, in order not to deplete the pool of potential reviewers, we would start trying to recruit potential reviewers at the beginning of January 2026.

Sincerely,

Dimitris Typas
Senior Editor
Nature Structural & Molecular Biology
ORCID: 0000-0002-8737-1319

Version 1:

Decision Letter:

9th Feb 2026

Dear Dr. Allegretti,

Thank you again for submitting your manuscript "Molecular architecture and spatial organisation of proteasomes in the human sperm nucleus". We now have comments (below) from the 4 reviewers who evaluated your paper. Please note that Reviewer #2 co-reviewed the manuscript with one of the other experts, as part of the Early Career Research initiative taking place at the Nature portfolio. In light of these reports, we remain interested in your study and would like to see your response to the comments of the referees in the form of a revised manuscript and detailed point-by-point response to all points raised.

You will see that though all reviewers appreciate the potential biological insight imparted by the study and how well supported the conclusions are, they still raise important concerns that must be comprehensively addressed in a revised manuscript. In particular, Reviewer #4 brings up technical concerns with respect to the need for different fitting and improved, more convincing models in certain cases, as well as clarifying certain ambiguous claims. On the other hand, Reviewer #1 and #3 request that you solidify the functional conclusions of the study by providing evidence for the cellular localisation and physiological relevance of the α4s* subunit, its relative abundance and connection to α4 and α4s, as well as convincingly substantiating its composition, including using mass spectrometry. Finally, the experts point out the need to improve figure presentation and correct at places both textual and figure errors.

Our standard revision period is 4 months. We appreciate that the requested revisions are extensive, particularly given the request to provide sufficient functional validation of the findings. We are committed to providing a fair and constructive peer-review process and encourage you to contact us if you would like to discuss the reviews or anticipate any delay with the resubmission.

We will be happy to consider your revision as long as nothing similar has been accepted for publication at NSMB or published elsewhere. Should your manuscript be substantially delayed without notifying us in advance and your article is eventually published, the received date would be that of the revised, not the original, version.

REPORTING AND DATA AVAILABILITY: Please provide updated files as follows with your revision. As you already know, we put great emphasis on ensuring that the methods and statistics reported in our papers are correct and accurate.

1. REPORTING SUMMARY: Please provide an updated file:

https://www.nature.com/documents/nr-reporting-summary.pdf

This form is a dynamic 'smart pdf' and must be downloaded and completed in Adobe Reader.

2. REPORTING OF STRUCTURAL DATA: If there are additional or modified structures in the revision, please submit the corresponding PDB validation reports with your other files, and use the figshare integration system to provide access to maps, half-maps, and models.

Manuscripts reporting new structures should contain a table summarizing structural and refinement statistics. Please include these tables for new cryo-EM or -ET (https://www.nature.com/documents/nr-tables-cryo-em.doc and modifying this file for ET), NMR (https://www.nature.com/documents/nr-tables-nmr.doc) and X-ray crystallography (https://www.nature.com/documents/nr-tables-xray.doc). To facilitate assessment of the quality of the structural data, a stereo image of a portion of the electron density map (for crystallography papers) or of the superimposed lowest energy structures (>10; for NMR papers) should be provided with the submitted manuscript. If the reported structure represents a novel overall fold, a stereo image of the entire structure (as a backbone trace) should also be provided. For cryo-EM structures, a representative micrograph showing individual particles should be provided in the figures alongside a processing workflow (please see our Editorial for more information: https://www.nature.com/articles/s41594-025-01567-9).

DATA DEPOSITION: We require deposition of coordinates (and, in the case of crystal structures, structure factors) into the Protein Data Bank with the designation of immediate release upon publication (HPUB). Electron microscopy-derived density maps and coordinate data must be deposited in EMDB and released upon publication. Deposition and immediate release of NMR chemical shift assignments are requested. To avoid delays in publication, dataset accession numbers must be supplied with the revised manuscript and appropriate release dates must be indicated at the galley proof stage.

3. REPORTING OF LIGHT MICROSCOPY DATA: For any revision that includes light microscopy data, we ask our authors to please include a completed light microscopy reporting table [https://www.nature.com/documents/Light_microscopy_reporting_table.xlsx] to ensure the methods are described thoroughly. The table will be available to reviewers and ultimately published, should the manuscript be accepted at the journal. When submitting the revised version of your manuscript, please pay close attention to our href="https://www.nature.com/nature-portfolio/editorial-policies/image-integrity">Digital Image Integrity Guidelines.

4. CODE AND COMPUTATIONAL WORK: If newly developed, unpublished code was used in this work, it must be provided with the revision and access to it must be disclosed in the Code Availability Statement in the manuscript. Please also provide the completed software submission checklist. This form is a dynamic 'smart pdf' and must be downloaded and completed in Adobe Reader, instead of opening it in a web browser: https://www.nature.com/documents/nr-software-policy.pdf

If molecular dynamics (MD) simulations were performed, please refer to this Editorial and provide a completed MD simulations checklist with your revision: https://www.nature.com/articles/s42003-023-04653-0

Lastly, if it applies, please complete and upload the completed machine learning checklist with your submission for re-review: https://www.nature.com/documents/machine-learning-checklist.pdf

5. SOURCE GEL IMAGES: Unprocessed scans should be provided for all gels and western blots presented in figures and should be clearly labelled with the figure number in the file title. The source gel images should be uncropped, unmodified images, with molecular weight markers. Uncropped, unmodified gel images from existing experimental repeats, in addition to the data shown in the figures, can also be presented in source data. Please provide these files as PDF with your revision.

Please ensure that all control panels for gels and western blots are appropriately described as loading or IP controls. Lastly, please ensure that all images in the paper are checked for duplication of panels and for splicing of gel lanes.

6. SOURCE NUMERICAL DATA: we urge authors to provide, in tabular form in excel files, the data underlying the graphical representations used in figures. This is to further increase transparency in data reporting, as detailed in this editorial

(http://www.nature.com/nsmb/journal/v22/n10/full/nsmb.3110.html). Please provide one excel file per figure max, with one panel per tab. When submitting files, the title field should indicate which figure the source data pertains to.

7. DATA AVAILABILITY: this journal strongly supports public availability of data. All data used in accepted papers should be available via a public data repository, or alternatively, as Supplementary Information. If data can only be shared on request, please explain why in your Data Availability Statement and in the correspondence with your editor.

For some data types, deposition in a public repository is mandatory as detailed below:

https://www.nature.com/nature-research/editorial-policies/reporting-standards#availability-of-data

Finally, please ensure that you retain unprocessed data and metadata files after publication, ideally archiving data in perpetuity, as these may be requested during the peer review and production process or after publication if any issues arise.

EXTENDED DATA FIGURES

When re-submitting your manuscript, please ensure that any supplementary figures and tables that are crucial to the manuscript's conclusions are converted into Extended Data figures and tables to increase visibility of these data. Extended Data figures and tables are online-only (present in the online PDF and full-text HTML versions of the paper), peer-reviewed display items that provide essential background to the article but are not included in the main article due to space constraints. A maximum of ten Extended Data display items (figures and tables) is permitted.

While we encourage the use of color in preparing figures, please note that this will incur a charge to partially defray the cost of printing. Information about color charges can be found at http://www.nature.com/nsmb/authors/submit/index.html#costs

Nature Structural & Molecular Biology is committed to improving transparency in authorship. As part of our efforts in this direction, we are now requesting that all authors identified as 'corresponding author' on published papers create and link their Open Researcher and Contributor Identifier (ORCID) with their account on the Manuscript Tracking System (MTS), prior to acceptance. This applies to primary research papers only. ORCID helps the scientific community achieve unambiguous attribution of all scholarly contributions. You can create and link your ORCID from the home page of the MTS by clicking on 'Modify my Springer Nature account'. For more information please visit please visit www.springernature.com/orcid.

Please use the link below to submit your revised manuscript and related files:

Link Redacted

Note: This URL links to your confidential home page and associated information about manuscripts you may have submitted, or that you are reviewing for us. If you wish to forward this email to co-authors, please delete the link to your homepage.

We look forward to seeing the revised manuscript and thank you for the opportunity to review your work.

Sincerely,

Dimitris Typas
Senior Editor
Nature Structural & Molecular Biology
ORCID: 0000-0002-8737-1319

Reviewers' Comments:

Reviewer #1 (Remarks to the Author):

This manuscript presents an impressive and technically rigorous structural and imaging analysis of nuclear proteasome organization and function in mammalian sperm, combining in situ cryo-ET, single-particle cryo-EM, and tissue-level imaging. The identification of large proteasome-rich nuclear lacunae, the structural characterization of native human sperm nuclear 20S and 20S–PA200 complexes at high resolution, and the observation of a substrate-like density in the β2 active site together provide novel and biologically meaningful insights into proteasome specialization during spermatogenesis. The work is carefully executed, and the conclusions are generally well supported by the data.

The authors note that the 26S proteasome was only observed near the sperm tail, but do not provide data supporting this. The authors should include this data in a figure in the manuscript showing where they are located in the tomograms and in what quantity. Additionally, the authors purify the proteasome complexes using size exclusion chromatography, but only show data corresponding to 2 fractions. The authors should include the data (immunoblot, negative stain EM) for other fractions to demonstrate that other proteasomal complexes (such as 26S) are not missed during sample purification.

One of the most intriguing aspects of the manuscript is the observation of what appears to be a peptide in the beta2 active site. The authors model the peptide as containing an arginine but do not provide additional validation of this. Mass spectrometry of the sample could help to confirm the identity of the peptide and strengthen the author's conclusions. Furthermore, mass spectrometry could also reveal if there are post-translational modifications on the proteasome that could be contributing to the trapping of what appears to be a cleavage intermediate, which has not be observed in other studies of native proteasomes.

Minor points

Fig. 1a, no arrows in figure

Fig. 3a, red spheres are not described in the legend

Fig. 3c, specific residues that are different should be labeled

Fig. 3h, colors should be changed to enhance clarity, text needs to be enlarged, description should be included in the main text

Fig. 4b, PDB ID for somatic: APO structure should be included in the legend

Under section "A tripeptide in the beta2 subunit active site reveals…", the authors state that the tripeptide bind similar to inhibitor-bound and propeptide-containing structures. A figure comparing these should be included.

Section "Single-particle cryo-EM reveals unique features of proteasomes isolated from human sperm cells", last paragraph, in sentence "Since the 20S proteasome complex was not detected…". Are the authors referring to 26S and not 20S?

Extended Data Fig 4, data workflow shows 151k particle images expanding to 222k particle images, is this an error?

Please verify the reference to Fig. 3c in the section "Human proteasome lacunae with 20S and PA200 form during spermatid stage of spermatogenesis", as it appears to point to a structural figure rather than tissue imaging.

Please also verify the reference to Fig. 4c in this section, as it appears to refer to a structural figure rather than immunohistochemistry data.

Reviewer #2 (Remarks to the Author):

I co-reviewed this manuscript with one of the reviewers who provided the listed reports. This is part of the Nature Structural & Molecular Biology initiative to facilitate training in peer review and to provide appropriate recognition for Early Career Researchers who co-review manuscripts.

Reviewer #3 (Remarks to the Author):

In this study, Kolata et al. performed in situ electron microscopy analyses of human sperm cells and discovered that proteasomes form distinct clusters within DNA-free cavities of the sperm nucleus. They further demonstrate that nuclear proteasomes in sperm predominantly exist as 20S core particles, with a subset forming complexes with the proteasome activator PA200. In addition, the authors identify the incorporation of a splicing variant of the testis-specific α4s subunit, α4s*, into sperm proteasomes. Based on these structural observations, the study provides important insights into the molecular mechanism by which association with PA200 enhances trypsin-like proteasome activity. The authors also document the clustering of 20S proteasomes and PA200 in post-meiotic stages of spermatogenesis, suggesting a regulated spatial organization during sperm differentiation.

These findings substantially extend previous biochemical knowledge of sperm proteasomes by revealing their detailed structural features and in situ organization. This study has high academic significance and may also have important implications for the field of reproductive biology and medicine. The experiments are generally well designed and appropriately conducted. Given the broad readership of Nature Structural & Molecular Biology, the study has strong potential for publication in this journal; however, several additional experiments and revisions to the text and figures would be necessary to more clearly convey the authors' conclusions and the significance of their findings.

Major comments

The characterization of the α4s splicing variant, α4s*, is currently limited to structural analyses, and additional validation at the gene expression level is necessary. The authors should perform RT–PCR analyses using sperm cells to directly compare the mRNA expression levels of α4, α4s, and α4s*.
In particular, given that α4s has previously been received as a sperm-specific proteasome subunit, it is important to conduct a detailed comparison of both the mRNA and amino acid sequences of α4s and α4s*. Such analyses would help clarify

whether the subunit previously annotated as α4s in earlier studies may in fact correspond to α4s*. Addressing this point would substantially strengthen the conclusions regarding the identity and specificity of the proteasome subunits present in human sperm.

Minor comments

1. In the Results section, the sentence “Since the 20S proteasome complex was not detected within nuclear lacunae (Fig. 1d), they likely originate from the sperm tail.” appears to contain an error. The reference to the 20S proteasome should likely be 19S, and this should be corrected accordingly.

2. In the Results section, the sentence “Interestingly, there was an enrichment in nuclear 20S core particles as differentiation progressed, with significantly higher levels in the nucleus of spermatocytes and spermatids, relative to spermatogonia (Fig. 3c and Extended Data Fig. 9b).” appears to cite an incorrect figure. The reference to Fig. 3c should likely be Fig. 5c, and this should be corrected accordingly.

Reviewer #4 (Remarks to the Author):

The manuscript entitled “Molecular architecture and spatial organization of proteasomes in the human sperm nucleus” by Piotr Kolata et al. is very interesting, has solid results, and makes an impression with its illustrations, even if some of them seem to be somewhat overloaded with information. The insight into the molecular architecture and its organization with subnanometer resolution of the sperm nucleus is definitely new, and the proteasome analysis has been carried out competently.
I believe that the manuscript is well suited for publication in NSMB, but I also believe that some revisions would be necessary. Below are my detailed comments and suggestions.

Introduction

I numbered the lines of each page in the document, and numbered the pages to point at he exact location.
Introdcution:
Page 1, Line 40, While I do like the Fonseca 2012 reference, here Groll et al 1997 I a much better fit, that is the original ref for the 20S.
Results:
Page 2, Line 55, The figure legend for 1A states 'arrows indicate regions with sperm lacunae' but seems to mean 'rectangles' and no arrowheads are visible. Please correct in the text an in the figure legend.
Page 3, line 2, not necessary to point to Fig 1 again - can be removed.
Page 3, line 8, It would be nice to see one example of double-capped PA200-20S in Fig. 1C. Also, given the low abundance - can the authors discuss the chances of these 0.3% to be true or false positives? Again, one or two examples in 1C would be enough.
Minor comment: Fig.1d: subunit coloring is spilling over to neighbouring subunits (e.g. the orange for a4s)
Page 3, line 12, Fig. 2b: the coloring of the lacunae is hardly visible in the lower panels. The upper panels are hard to navigate. The nucleus is only recognizable after spotting the colored dots in the lower panel. Needs better markings, better presentation. AND: the video2 uses different colors for lacunae than in the figure, why?
Page 4, line 6-7, Needs to be rephrased. “Micrographs allowed us” sounds odd. Fig.3: some panels are too densely packed, their markings are overlapping into other panels. Figure needs more space or should be better arranged to avoid overlapping. I would suggest to make Fig a, b, c, f, g and h page width large and place Fig 3 d and I somehow below.
Page 4, line 12 - 15, great finding great statement.
Page 4, line 30, Extended data Fig 7c, Labelling of alpha4-R4 in lower panel is inconclusive.
Page 4, line 33, Extended data Fig 7d-f, Water molecule color should be black, not green.
Page 4, line 34, Fig. 3f: no difference discernable because image is very small.
Page 4, line 38, Fig. 3h: Using orange and dark-orange is not different enough.
Page 4, line 41, Fig. 3i: Why is “PA200” spelled with two different colors?
Page 4, line 45-53:
This is a questionable 26S structure, even for a AAA-ATPase-only 19S. To be convinced I would need to see the fitted pdb of the AAA-Atpase aligning well with some helices of the density. This should be possible at 3.2A.
Due to flexibility? I think the 19S is rigid enough. It is more likely that a) the density is a mixture of PA200-20S and 19S-20S or b) the 19S is not fully assembled and only carries the AAA-ATPase.
Also problematic: it seems like the upper AAA-ATPase ring is almost not present, it seems to be less well occupied (which is not possible, it is the N-terminal part).
The authors should show a fit of the AAA-Atpase-ring in the proposed 26S map. This model does not look completely reliable.
Update: ext. Fig. 8e and 8f show a bit more of the AAA-Atpase density and their pdb fit seems to correlate well. This suggests, they have also 20S with AAA-Atpase - but I wouldn't call it "26S".
Page 5, line 5, Fig. 4a-c: Is'nt the rotation from b to c 180 deg and not 90 as indicated in the figure?
Fig. legend
b) “rearrangement required“ should read “rearrangement is required“

d) "the structure of β2 subunit" should read "the structure of the β2 subunit"
d) "In the left panel, the same view as in b is
used." d seems to be identical to c, not b
Page 6, line 9-10, PRMC1 is used to test for 19S localization, however, this is a AAA-Atpase subunit and does not clarify whether a fully assembled 19S or only an AAA-Atpase is present. In order to claim "19S" without further explanation I suggest to use at least one other subunit as marker (Rpn10, Rpn1, Rpn11…)
Page 7, line 7-10: Can you elaborate on the implications?
Page 7, line 55, Why is the tripeptide structure so well resolved? Shouldn't the density wash out in case different tripeptides are being processed? Why do all proteasomes show the same tripeptide?
If it is a trapped intermediate then it should be explained why this particular tripeptide is so likely to be the trap.
Page 8, line 17, '19S was enriched' - I think it is AAA-ATPase not 19S, see also comments above.
Page 8, line 21, 'our map' is missing all other subunits, including Rpn1 which is close to alpha4.
Page 9, line 37-40: It is okay to cite its own papers, however, the original protocols are derived from others. One may refer to the original publications for lamella milling. I think the authors know what I am aiming for.
Page 10, line 2, correct 'weigthed' to weighted.
Page 10, line 5, delete 'and', 'extensive'.
Page 10, line 9- : I think the information: 'to obtain a 20S map at 6.8 Å resolution; a 20S-PA200 map at 9.5 Å resolution; and a PA200-20S-PA200 map at 14.4 Å resolution.' Can be deleted out of the methods section.
Page 11, line 42: exchange picking with picks and remove change to positive not positives.

Version 2:

Decision Letter:

Our ref: NSMB-A52086B

6th Jul 2026

Dear Dr. Allegretti,

Thank you for submitting your revised manuscript "Molecular architecture and spatial orgMolecular architecture and spatial organisation of proteasomes in the human sperm nucleus anisation of proteasomes in the human sperm nucleus" (NSMB-A52086B). It has now been seen by the original referees and their comments are below. The reviewers find that the paper has improved in revision, and therefore we are happy to accept it in principle for publication in Nature Structural & Molecular Biology, pending minor revisions to satisfy the referees' final requests and to comply with our editorial and formatting guidelines.

We will be soon performing detailed checks on your paper and will send you a checklist detailing our editorial and formatting requirements in a about a week. Please do not upload the final materials and make any revisions until you receive this additional information from us.

To facilitate our work at this stage, it is important that we have a copy of the main text as a word file. If you could please send along a word version of this file as soon as possible, we would greatly appreciate it; please make sure to copy the NSMB account (cc'ed above).

Thank you again for your interest in Nature Structural & Molecular Biology Please do not hesitate to contact me if you have any questions.

Sincerely,

Dimitris Typas
Senior Editor
Nature Structural & Molecular Biology
ORCID: 0000-0002-8737-1319

Reviewer #1 (Remarks to the Author):

All issues have been addressed. The manuscript is suitable for publication.

Reviewer #2 (Remarks to the Author):

I co-reviewed this manuscript with one of the reviewers who provided the listed reports. This is part of the Nature Structural & Molecular Biology initiative to facilitate training in peer review and to provide appropriate recognition for Early Career Researchers who co-review manuscripts.

Reviewer #3 (Remarks to the Author):

The authors have addressed the reviewers' comments comprehensively and thoughtfully, and I believe that the manuscript has reached the standard required for publication. The additional experiments, analyses, and revisions have substantially strengthened the study, and I support acceptance. I have only one minor suggestion that may further strengthen the interpretation of the α4s* findings.

The authors made a reasonable effort to assess α4s* expression using RNA extracted from mature spermatozoa; however, the results were understandably inconclusive given the transcriptionally inactive state of mature sperm cells. Since the α4s* protein incorporated into sperm proteasomes must be synthesized during spermatogenesis, expression of the corresponding transcript would be expected at earlier developmental stages in the male germline.

If suitable material or publicly available transcriptomic datasets are readily available (e.g., testis tissue, spermatogenic cells, or published human/mouse RNA-seq datasets), additional evidence supporting α4s* transcript expression during spermatogenesis would further strengthen the interpretation. Alternatively, if transcript-level validation is not feasible, I encourage the authors to more explicitly discuss the structural basis for distinguishing α4s* from α4s. In particular, it would be helpful to clarify whether the local map quality and resolution in the isoform-discriminating regions are sufficient to support the assignment with high confidence, and to acknowledge any limitations if appropriate.

I do not consider this point essential for publication, as the current structural and proteomic evidence already provides substantial support for the authors' conclusions. Nevertheless, additional clarification along either of these lines would further strengthen the manuscript and help readers assess the robustness of the α4s* assignment.

Reviewer #4 (Remarks to the Author):

The revised version has been greatly improved, and most of the reviewers' comments have been addressed. In my opinion, the data is outstanding, even if some aspects still leave room for speculation. I am convinced that this manuscript is now ready for publication.

INTERNAL USE ONLY AIP modules required: ["Life Science"]

Version 3:

Decision Letter:

28th Jul 2026

Dear Dr. Allegretti,

We are now happy to accept your revised paper "Molecular architecture and spatial organisation of proteasomes in the human sperm nucleus" for publication as an Article in Nature Structural & Molecular Biology.

Acceptance is conditional on the manuscript's not being published elsewhere and on there being no announcement of this work to the newspapers, magazines, radio or television until the publication date in Nature Structural & Molecular Biology.

Over the next few weeks, your paper will be copyedited to ensure that it conforms to Nature Structural & Molecular Biology style. Once your paper is typeset, you will receive an email with a link to choose the appropriate publishing options for your paper and our Author Services team will be in touch regarding any additional information that may be required.

After the grant of rights is completed, you will receive a link to your electronic proof via email with a request to make any corrections within 48 hours. If, when you receive your proof, you cannot meet this deadline, please inform us at rjsproduction@springernature.com immediately.

You will not receive your proofs until the publishing agreement has been received through our system.

Due to the importance of these deadlines, we ask that you please let us know now whether you will be difficult to contact over the next month. If this is the case, we ask you provide us with the contact information (email, phone and fax) of someone who will be able to check the proofs on your behalf, and who will be available to address any last-minute problems.

To assist our authors in disseminating their research to the broader community, our SharedIt initiative provides all co-authors with the ability to generate a unique shareable link that will allow anyone (with or without a subscription) to read the published article. Recipients of the link with a subscription will also be able to download and print the PDF.

As soon as your article is published, you can generate your shareable link by entering the DOI of your article here: http://authors.springernature.com/share. Corresponding authors will also receive an automated email with the shareable link

Note the policy of the journal on data deposition: http://www.nature.com/authors/policies/availability.html.

Your paper will be published online soon after we receive proof corrections and will appear in print in the next available issue. You can find out your date of online publication by contacting the production team shortly after sending your proof corrections.

You may wish to make your media relations office aware of your accepted publication, in case they consider it appropriate to organize some internal or external publicity. Once your paper has been scheduled you will receive an email confirming the publication details. This is normally 3-4 working days in advance of publication. If you need additional notice of the date and time of publication, please let the production team know when you receive the proof of your article to ensure there is sufficient time to coordinate. Further information on our embargo policies can be found here: https://www.nature.com/authors/policies/embargo.html

You can now use a single sign-on for all your accounts, view the status of all your manuscript submissions and reviews, access usage statistics for your published articles and download a record of your refereeing activity for the Nature journals.

If you have not already done so, we strongly recommend that you upload the step-by-step protocols used in this manuscript to the Protocol Exchange. Protocol Exchange is an open online resource that allows researchers to share their detailed experimental know-how. All uploaded protocols are made freely available, assigned DOIs for ease of citation and fully searchable through nature.com. Protocols can be linked to any publications in which they are used and will be linked to from your article. You can also establish a dedicated page to collect all your lab Protocols. By uploading your Protocols to Protocol Exchange, you are enabling researchers to more readily reproduce or adapt the methodology you use, as well as increasing the visibility of your protocols and papers. Upload your Protocols at www.nature.com/protocolexchange/. Further information can be found at www.nature.com/protocolexchange/about.

An online order form for reprints of your paper is available at https://www.nature.com/reprints/author-reprints.html. Please let your coauthors and your institutions' public affairs office know that they are also welcome to order reprints by this method.

Authors may need to take specific actions to achieve compliance with funder and institutional open access mandates. If your research is supported by a funder that requires immediate open access (e.g. according to Plan S principles or the NIH public access policy) then you should select the gold OA route, and we will direct you to the compliant route where possible. Because authors warrant under our subscription licensing terms that they haven't committed to licensing any version of their article under a licence inconsistent with the terms of our agreement – including the applicable embargo period – publication under the subscription model isn't suitable for authors whose funders require no embargo.

In approximately 10 business days you will receive an email with a link to choose the appropriate publishing options for your paper and our Author Services team will be in touch regarding any additional information that may be required.

You will not receive your proofs until the publishing agreement has been received through our system.

If you have any questions about our publishing options, costs, Open Access requirements, or our legal forms, please contact ASJournals@springernature.com

Sincerely,

Dimitris Typas
Senior Editor
Nature Structural & Molecular Biology
ORCID: 0000-0002-8737-1319

Click here if you would like to recommend Nature Structural & Molecular Biology to your librarian: http://www.nature.com/subscriptions/recommend.html#forms

Open Access This Peer Review File is licensed under a Creative Commons Attribution 4.0 International License, which permits use, sharing, adaptation, distribution and reproduction in any medium or format, as long as you give appropriate credit to the original author(s) and the source, provide a link to the Creative Commons license, and indicate if changes were made.
In cases where reviewers are anonymous, credit should be given to 'Anonymous Referee' and the source.
The images or other third party material in this Peer Review File are included in the article’s Creative Commons license, unless indicated otherwise in a credit line to the material. If material is not included in the article’s Creative Commons license and your intended use is not permitted by statutory regulation or exceeds the permitted use, you will need to obtain permission directly from the copyright holder.
To view a copy of this license, visit https://creativecommons.org/licenses/by/4.0/

Reviewers' Comments:

Reviewer #1 (Remarks to the Author):

This manuscript presents an impressive and technically rigorous structural and imaging analysis of nuclear proteasome organization and function in mammalian sperm, combining in situ cryo-ET, single-particle cryo-EM, and tissue-level imaging. The identification of large proteasome-rich nuclear lacunae, the structural characterization of native human sperm nuclear 20S and 20S–PA200 complexes at high resolution, and the observation of a substrate-like density in the β2 active site together provide novel and biologically meaningful insights into proteasome specialization during spermatogenesis. The work is carefully executed, and the conclusions are generally well supported by the data.

We would like to thank the reviewer for highlighting the quality and significance of our work.

The authors note that the 26S proteasome was only observed near the sperm tail, but do not provide data supporting this. The authors should include this data in a figure in the manuscript showing where they are located in the tomograms and in what quantity.

We agree that this point requires clarification. Our subtomogram averaging of proteasomes within nuclear lacunae shows that they contain 20S and 20S+PA200 populations, but did not identify any 26S particles. However, single-particle analysis of proteasomes purified from whole human sperm revealed a small population of 26S. In the revised manuscript, we analysed this second population in greater detail (Extended Data Fig.8h-i; Extended Data Fig. 9).
In the original manuscript we showed, using both immunocytochemistry of ejaculated human sperm and immunohistochemistry of human testes that the 19S subunit PSMC1 localises exclusively to the tail of mature sperm cells (Extended Data Fig. 3e, Extended Data Fig. 11e-j). We therefore conclude that the small 26S population observed following whole sperm purification originates from the tail rather than from the nuclear lacunae. To further support this conclusion, we now include additional immunocytochemistry data for a second 19S subunit, RPN1 (PSMD2), which similarly localises exclusively to the sperm tail (Extended Data Fig. 3f).

We have modified the text to make this point clearer. It now reads:

'We identified a small fraction of 26S proteasomes in the 20S-PA200 sample. Since 26S proteasomes were not detected by tomography within nuclear lacunae (Fig. 1d), they likely originate from the sperm tail. This is supported by immunofluorescence and immunohistochemistry data showing enrichment of 19S subunits in the tail of both ejaculated human sperm and mature spermatozoa in human testis tissue (Extended Data Fig. 3e-f; Extended Data Fig. 11e-j).'

Additionally, the authors purify the proteasome complexes using size exclusion chromatography, but only show data corresponding to 2 fractions. The authors should include the data (immunoblot, negative stain EM) for other fractions to demonstrate that other proteasomal complexes (such as 26S) are not missed during sample purification.

We now include both immunoblotting and negative-stain EM data showing that only two fractions were clearly enriched in proteasomes (Extended Data Fig. 4a-d). Although a small number of 26S-like particles could be observed in the B3 fraction by negative-stain EM, their abundance was insufficient for detection by immunoblotting (Extended Data Fig. 4d) or for preparation of cryo-EM samples.

One of the most intriguing aspects of the manuscript is the observation of what appears to be a peptide in the beta2 active site. The authors model the peptide as containing an arginine but do not provide

additional validation of this. Mass spectrometry of the sample could help to confirm the identity of the peptide and strengthen the author's conclusions.
Furthermore, mass spectrometry could also reveal if there are post-translational modifications on the proteasome that could be contributing to the trapping of what appears to be a cleavage intermediate, which has not be observed in other studies of native proteasomes.

We attempted to perform mass spectrometry. However, as also mentioned in our response to Reviewers #2 and #3 due to the low amount of sample available to us, we obtained very poor peptide coverage of proteasomal subunits. Specifically, for the beta2 subunit we obtained 3 unique peptides, and only 12% coverage. With this low abundance, we were not able to detect an additional covalent intermediate peptide at the catalytic site. We have added the following sentence to the Discussion section addressing the nature of the trapped tripeptide:

'However, further work will be required to validate this hypothesis and determine the biochemical identity of the peptide.'

Regarding our assignment of an Arg to the density protruding from the catalytic Thr in the beta2 subunit, this was based both on the structural features of our 1.85Å cryo-EM map and the known trypsin-like activity of this subunit. Importantly, beta2 preferentially cleaves peptide bonds on the carboxyl-terminal side of basic amino acids, primarily Arg or Lys, therefore we modelled both Lys and Arg into the density protruding from the catalytic Thr, which is shown in the figure below. In the top panel **(a)** we show the fit of this density to a Lys and in the bottom panel **(b)** we show the fit to an Arg residue. From our data, the density is more consistent with the guanidino group of an Arg than the amino group of Lys. Hence, we interpret the trapped tripeptide as containing an Arg residue. We have added to the Results section a brief description of this modelling which led to the assignment of Arg to the cryo-EM density:

'The β2 subunit exhibits trypsin activity, cleaving peptide bonds at the carboxy-terminal of basic amino acid residues, primarily Arg and Lys. For this reason, and based on our cryo-EM map, we modelled both Arg and Lys to the density. The fit obtained was consistent with an Arg rather than a Lys (data not shown) and, therefore we modelled the density as an Arg-containing tripeptide (Fig. 4a-c).'

Due to the limited peptide coverage of proteasomal subunits in our mass spectrometry data, we were unable to assess whether post-translational modifications might contribute to peptide trapping. We also examined our cryo-EM map for additional features extending from side chains at the pore that could indicate such modifications, but did not identify any plausible candidates. We have added the following statement to the Discussion:

'Post-translational modifications at the pore region or in the active site, acquired during sperm cell differentiation, could also affect product entry or release. However, close inspection of these regions did not reveal additional densities protruding from side chains. Nevertheless, further biochemical analysis and mass spectrometry studies might be important to answer this question.'

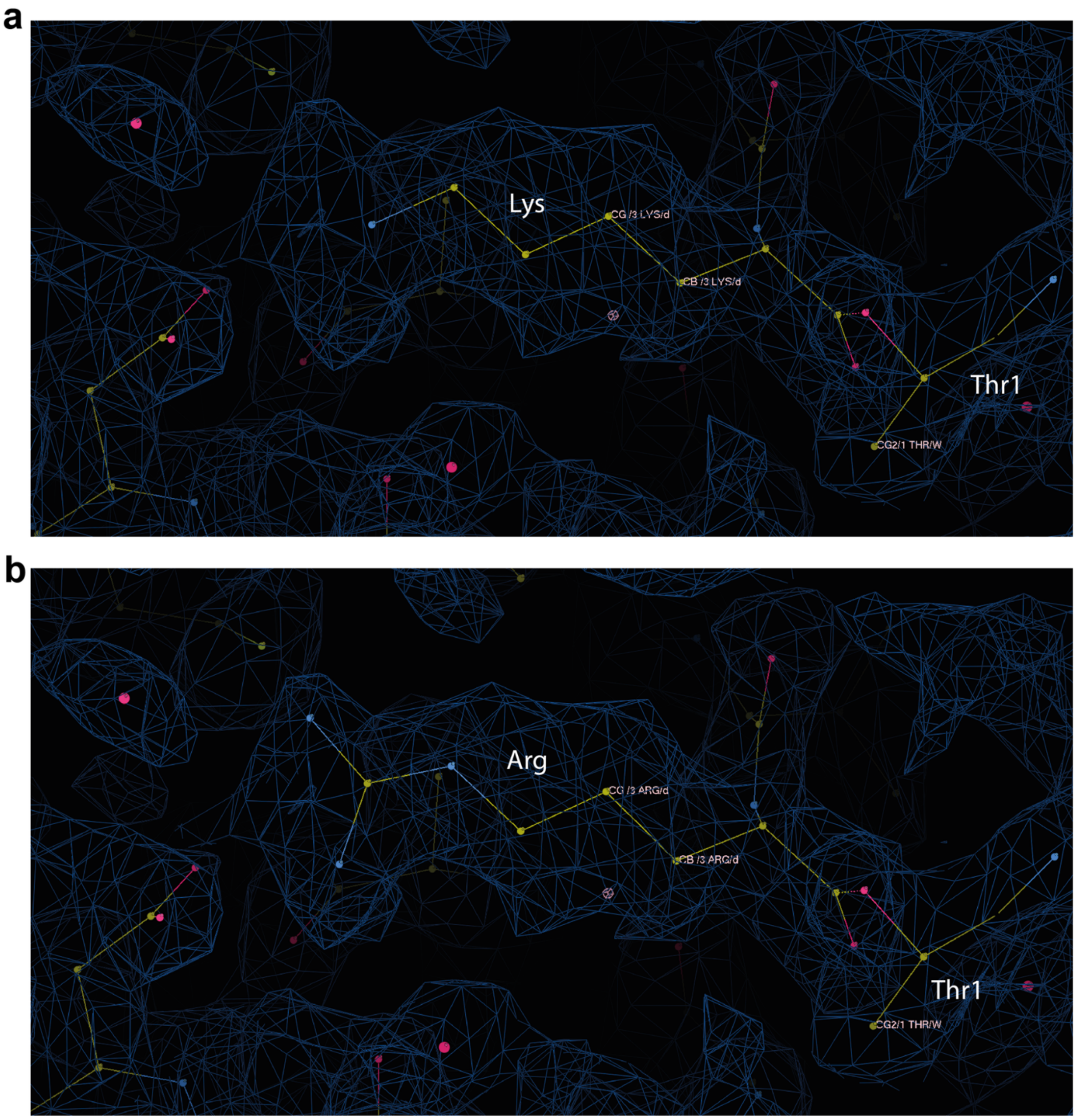

Figure showing fitting of density present at the catalytic subunit of beta2. **(a)** shows fitting of a Lys residue; **(b)** shows fitting of an Arg residue.

Minor points

Fig. 1a, no arrows in figure

We have corrected the legend for Fig. 1a legend.

Fig. 3a, red spheres are not described in the legend

The figure legend has been modified to explain that red spheres correspond to water molecules.

Fig. 3c, specific residues that are different should be labeled

We have modified Fig. 3c accordingly.

Fig. 3h, colors should be changed to enhance clarity, text needs to be enlarged, description should be included in the main text

We have adjusted the original Fig. 3h (now Fig. 3g in the revised manuscript) and added a description of the conformational changes in alpha4s in the main text (shown below):

'Gate opening is mediated via conformational changes in all α-subunits, equivalent to those in somatic 20S-PA200 (Fig. 3e-g, Extended Data Fig. 8a-d). α4s gets displaced (Extended Data Fig. 8b), its 3-18$^{\alpha4s*}$ and 117-127$^{\alpha4s*}$ loops become disordered, and the 49-63$^{\alpha4s*}$ loop shifts downwards by 6 Å (Fig. 3g, Extended Data Fig. 8b).'*

Fig. 4b, PDB ID for somatic: APO structure should be included in the legend

We have added this information to the figure legend.

Under section "A tripeptide in the beta2 subunit active site reveals…", the authors state that the tripeptide bind similar to inhibitor-bound and propeptide-containing structures. A figure comparing these should be included.

We have added a new figure to the new version of the manuscript, comparing the three binding modalities (Extended Data Fig. 10 is shown below).

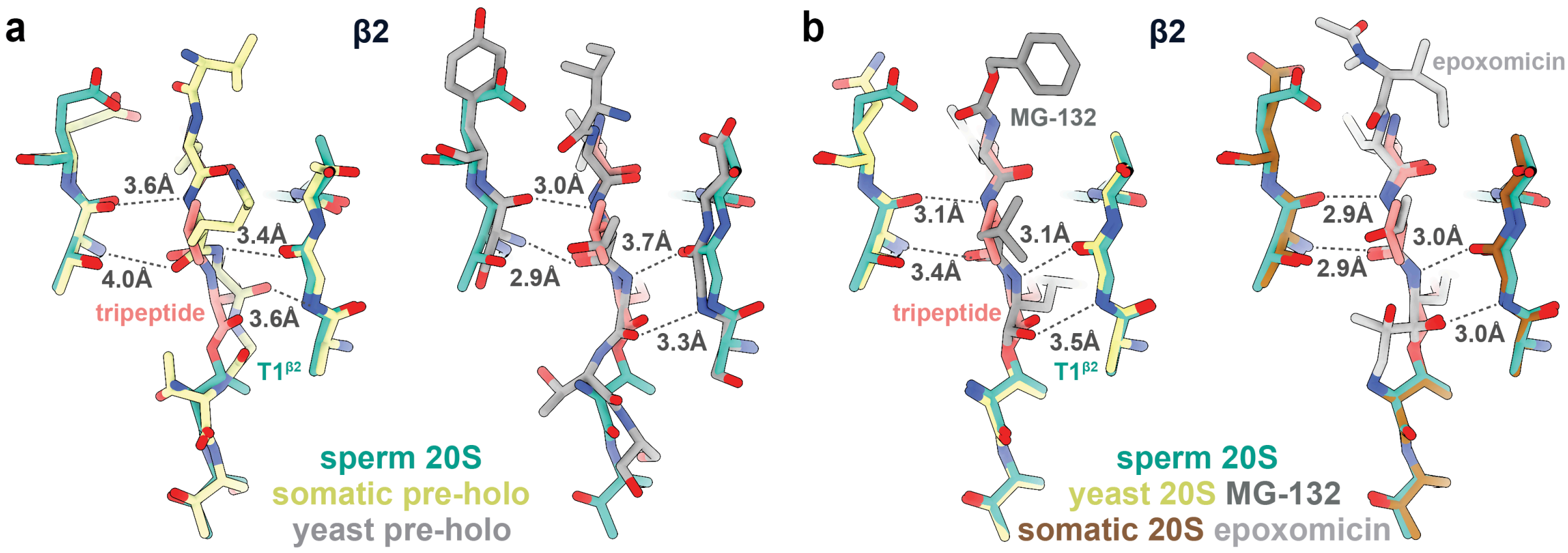

Extended Data Fig. 10. – The tripeptide in the β2 subunit is coordinated similarly to β2 propeptide and proteasomal inhibitors. a, the β2 propeptides in human pre-holo somatic proteasome (yellow, PDB ID: 8IYI) and yeast pre-holo proteasome (grey, PDB ID: 8U6Y) are coordinated similarly to tripeptide substrate (pink) in sperm 20S core particle (cyan). **b,** proteasomal inhibitors, MG-132 (dark grey, bound to the yeast 20S proteasome, yellow, PDB ID: 5FGH) and epoxomicin (light grey, bound to the human 20S somatic proteasome, brown, PDB ID: 5LF0) are coordinated similarly to tripeptide substrate (pink) in sperm 20S core particle (cyan). In **a** and **b,** the dashed lines indicate hydrogen bonding (distances shown).

Section "Single-particle cryo-EM reveals unique features of proteasomes isolated from human sperm cells", last paragraph, in sentence "Since the 20S proteasome complex was not detected…". Are the authors referring to 26S and not 20S?

We have corrected the text, which now reads:

We identified a small fraction of 26S proteasomes in the 20S-PA200 sample. Since 26S proteasomes were not detected by tomography within the nucleus (Fig. 1d), they likely originate from the sperm tail. This is supported by immunofluorescence and immunohistochemistry data showing enrichment of 19S subunits

in the tail of both ejaculated human sperm and mature spermatozoa in human testis tissue (Extended Data Fig. 3e-f; Extended Data Fig. 11e-j).

Extended Data Fig 4, data workflow shows 151k particle images expanding to 222k particle images, is this an error?

We agree that this point requires clarification. We have now edited the schematics in Extended Data Fig. 4 to explain that the difference in particle number between the two processing steps results from the merging of 20S particles from all datasets.

Please verify the reference to Fig. 3c in the section "Human proteasome lacunae with 20S and PA200 form during spermatid stage of spermatogenesis", as it appears to point to a structural figure rather than tissue imaging.

We have changed the text to now reference the correct figure - Fig. 5c.

Please also verify the reference to Fig. 4c in this section, as it appears to refer to a structural figure rather than immunohistochemistry data.

We have also changed the text accordingly. It now references the correct figure - Extended Data Fig. 3c.

Reviewer #2 (Remarks to the Author):

I co-reviewed this manuscript with one of the reviewers who provided the listed reports. This is part of the Nature Structural & Molecular Biology initiative to facilitate training in peer review and to provide appropriate recognition for Early Career Researchers who co-review manuscripts.

Reviewer #3 (Remarks to the Author):

In this study, Kolata et al. performed in situ electron microscopy analyses of human sperm cells and discovered that proteasomes form distinct clusters within DNA-free cavities of the sperm nucleus. They further demonstrate that nuclear proteasomes in sperm predominantly exist as 20S core particles, with a subset forming complexes with the proteasome activator PA200. In addition, the authors identify the incorporation of a splicing variant of the testis-specific α4s subunit, α4s*, into sperm proteasomes. Based on these structural observations, the study provides important insights into the molecular mechanism by which association with PA200 enhances trypsin-like proteasome activity. The authors also document the clustering of 20S proteasomes and PA200 in post-meiotic stages of spermatogenesis, suggesting a regulated spatial organization during sperm differentiation.

These findings substantially extend previous biochemical knowledge of sperm proteasomes by revealing their detailed structural features and in situ organization. This study has high academic significance and may also have important implications for the field of reproductive biology and medicine. The experiments are generally well designed and appropriately conducted. Given the broad readership of Nature Structural & Molecular Biology, the study has strong potential for publication in this journal; however, several additional experiments and revisions to the text and figures would be necessary to more clearly convey the authors' conclusions and the significance of their findings.

We would like to thank the reviewer for the positive assessment of our work and for taking the time to read our manuscript and provide comments.

Major comments

The characterization of the α4s splicing variant, α4s*, is currently limited to structural analyses, and additional validation at the gene expression level is necessary. The authors should perform RT–PCR analyses using sperm cells to directly compare the mRNA expression levels of α4, α4s, and α4s*. In particular, given that α4s has previously been received as a sperm-specific proteasome subunit, it is important to conduct a detailed comparison of both the mRNA and amino acid sequences of α4s and α4s*. Such analyses would help clarify whether the subunit previously annotated as α4s in earlier studies may in fact correspond to α4s*. Addressing this point would substantially strengthen the conclusions regarding the identity and specificity of the proteasome subunits present in human sperm.

We performed qPCR analyses on RNA extracted from human sperm cells to test for transcripts specific for α4s*, α4s and canonical α4 (PSMA7). The caveat of this experiment is that mature sperm cells are transcriptionally inactive (PMID: 17903313), and most mRNA is degraded during spermatid differentiation (PMID: 24787618). Consistent with this, RNA yields from sperm were substantially lower than those obtained from parallel extractions from somatic cells, using human skin fibroblasts as a control.

To provide a positive control for α4s* we also generated a human fibroblast cell line stably expressing the α4s* splice isoform. We then proceeded with qPCR analysis for α4s*, α4s and α4 transcripts. For all experiments we used the same initial amount of input RNA for cDNA synthesis and subsequent qPCR analyses. WT fibroblasts and α4s*-expressing fibroblasts were analysed in parallel with sperm samples by qPCR. As expected, α4 (PSMA7) transcripts were readily detected in WT fibroblasts and in α4s*-expressing fibroblasts (shown in the figure below, panel **a)**. Low levels of α4 transcripts could also be detected in sperm cells, although expression was substantially lower and more variable between replicates (n=4, shown in the figure below, panel **a**). In contrast, we were unable to detect transcripts for PSMA8 isoforms (α4s* or α4s) in sperm samples (shown in the figure below, panels **b** and **c)**. Importantly, α4s* transcripts were robustly amplified from fibroblasts stably expressing α4s*, validating our primer and assay design, while WT fibroblasts showed no amplification of either α4s* or α4s, as expected (shown in the figure below, panel **b** and **c)**. As an additional control, we amplified the transcript for the sperm-enriched protein protamine, PRM1 (shown in figure below, panel **d**), despite the low RNA yield, validating our protocol for low-yield sperm RNA. As a further control, we also analysed LMNA. Lamin A/C is abundantly expressed at the sperm nuclear envelope (preprint DOI: 10.1101/2024.10.30.620797 and figure below, panel **f**). LMNA transcripts could be detected in sperm samples, albeit at substantially lower abundance and with greater variability than in WT and α4s*-expressing fibroblast controls (data shown in figure below, panel **e**).

Overall, these qPCR analyses did not provide conclusive evidence regarding the relative expression of PSMA8 isoforms in mature sperm cells. Given the transcriptionally inactive state of sperm cells and the limited abundance residual mRNA, we believe that the mature sperm RNA is not suitable for reliable assessing levels of PSMA8 splice variants, as such, transcript abundance in sperm cells does not appear to directly correlate with protein abundance.

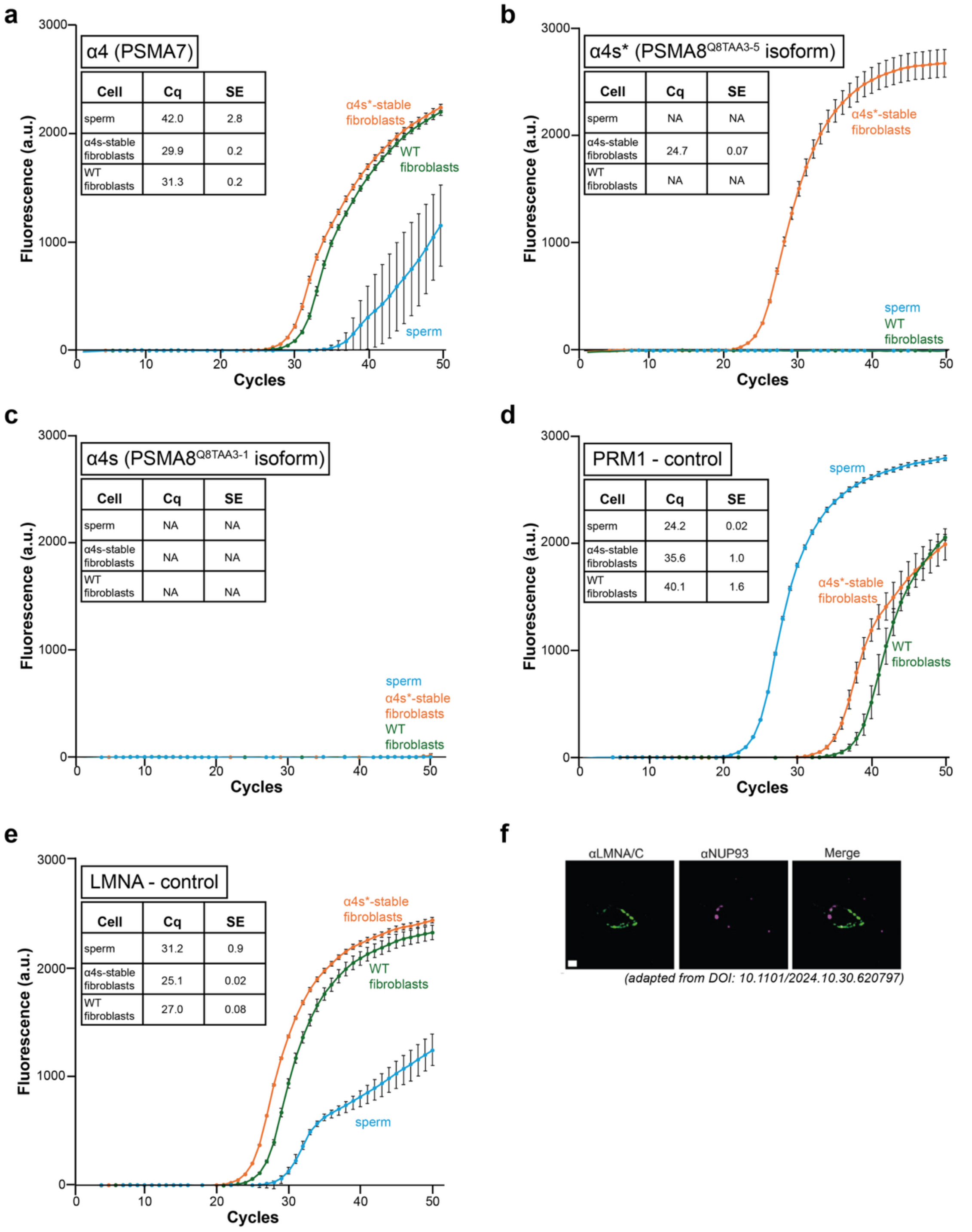

Figure showing qPCR analyses of RNA extracted from human sperm cells, human skin fibroblasts and human skin fibroblasts stably expressing α4s*. Transcripts for **a.** α4; **b.** α4s*; **c.** α4s; **d.** PRM1 and **e.** LMNA were analysed. Calculated Cq values are provided for each cell type with standard error (SE), n=4. **f.** SIM image of human sperm cell with immunostaining for Lamin A/C (green) showing abundant expression at the nuclear envelope and co-stained with nucleoporin NUP93 (magenta) which is present at the rear of the nucleus. Adapted from DOI: 10.1101/2024.10.30.620797.

We also attempted mass spectrometry analysis of purified sperm proteasomes, to identify unique peptides for α4s*/α4s. However, the amount of material available for these experiments was limited, as our study exclusively uses donor sperm samples that have been cleared for in vitro fertilisation and approved for research use following quality control procedures, including morphology assessment, swim-up selection and screening for blood-borne viruses. Consequently, the quantity of purified proteasomes was insufficient to confidently detect and validate low-abundance isoform-specific

unique peptides by mass-spectrometry. For example, for α4s*/α4s we obtained one unique peptide (not able to distinguish between the two isoforms), with 14% sequence coverage, and for α4 we obtained two unique peptides at 20% sequence coverage.

However, a more careful search through the literature revealed a published proteomics dataset for human sperm cells (PMID: 31244561) where 18 peptides for the α4s* splice variant were found. Conversely, in this same study, no unique peptides for α4s were found. We have added a table summarising these results below (table 1, *adapted from* PMID: 31244561) and now cite this study in the Discussion. This previously published dataset is consistent with our structural observations and provide independent support for the presence of α4s* and not α4s in mature human sperm proteasomes. Interestingly, the authors also found unique peptides for α4 in their proteomics data (PMID: 31244561). Our 1.85Å 20S map revealed, unambiguously, a proteasome population with α4s*. For the 26S map, whilst we can rule out the presence of α4s, at the present resolution, we cannot distinguish between the presence of the canonical α4 or α4s*. We have added this discussion point to our revised manuscript (new text shown below):

'Our near-atomic resolution cryo-EM structure of the 20S proteasome CP (Fig. 3a-d; Extended Data Fig. 5f; Extended Data Fig. 6; Extended Data Fig. 7) reveals the exclusive presence of a specific splice variant in human, which we term α4s. Interestingly, a published proteomics dataset from human sperm cells identified unique peptides for the same splice variant (α4s*) but did not identify unique peptides for α4s in human spermatozoa (Li et al., 2019), consistent with our structural observations.'*

We also added to the Results section the following sentence, regarding our 26S cryo-EM map:

'Due to the resolution, we could not distinguish between the presence of α4 or α4s in our map, but we can exclude the presence of α4s.'*

Table 1 - *Extracted from Additional file 1: Table S1 of PMID:31244561*. Proteasome-related hits from total proteins idenfied in human sperm by proteomics.

Name and isoform (if applicable)	Accession	Peptides	%coverage
Proteasome subunit alpha type-1 OS=Homo sapiens GN=PSMA1 PE=1 SV=1	sp|P25786|PSA1_HUMAN	23	72.2
Proteasome subunit alpha type-2 OS=Homo sapiens GN=PSMA2 PE=1 SV=2	sp|P25787|PSA2_HUMAN	22	64.5
Proteasome subunit alpha type-3 OS=Homo sapiens GN=PSMA3 PE=1 SV=2	sp|P25788|PSA3_HUMAN	21	58.0
Proteasome subunit alpha type-4 OS=Homo sapiens GN=PSMA4 PE=1 SV=1	sp|P25789|PSA4_HUMAN	18	51.0
Proteasome subunit alpha type-5 OS=Homo sapiens GN=PSMA5 PE=1 SV=3	sp|P28066|PSA5_HUMAN	25	69.3
**Proteasome subunit alpha type-7 OS=Homo sapiens GN=PSMA7 PE=1 SV=1**	sp|O14818|PSA7_HUMAN	17	62.9
**Isoform 3 of Proteasome subunit alpha type-7-like OS=Homo sapiens GN=PSMA8**	**sp|Q8TAA3-5|PSA7L_HUMAN**	**18**	**72**
Proteasome subunit beta type-1 OS=Homo sapiens GN=PSMB1 PE=1 SV=2	sp|P20618|PSB1_HUMAN	20	59.3
Proteasome subunit beta type-2 OS=Homo sapiens GN=PSMB2 PE=1 SV=1	sp|P49721|PSB2_HUMAN	8	47.3
Proteasome subunit beta type-3 OS=Homo sapiens GN=PSMB3 PE=1 SV=2	sp|P49720|PSB3_HUMAN	14	42.4
Proteasome subunit beta type-4 OS=Homo sapiens GN=PSMB4 PE=1 SV=4	sp|P28070|PSB4_HUMAN	21	57.2
Proteasome subunit beta type-5 OS=Homo sapiens GN=PSMB5 PE=1 SV=3	sp|P28074|PSB5_HUMAN	17	58.9
Proteasome subunit beta type-6 OS=Homo sapiens GN=PSMB6 PE=1 SV=4	sp|P28072|PSB6_HUMAN	10	46.0
Proteasome subunit beta type-7 OS=Homo sapiens GN=PSMB7 PE=1 SV=1	sp|Q99436|PSB7_HUMAN	12	55.6
Proteasome subunit beta type OS=Homo sapiens GN=PSMB9 PE=2 SV=1	tr|A2ACR1|A2ACR1_HUMAN	1	26.5
Proteasome subunit beta type-10 OS=Homo sapiens GN=PSMB10 PE=1 SV=1	sp|P40306|PSB10_HUMAN	2	20.9
Proteasome activator complex subunit 4 OS=Homo sapiens GN=PSME4 (PA200) PE=1 SV=2	sp|Q14997|PSME4_HUMAN	35	27.4
26S proteasome non-ATPase regulatory subunit 1 OS=Homo sapiens GN=PSMD1 PE=1 SV=2	sp|Q99460|PSMD1_HUMAN	37	48.7
26S proteasome non-ATPase regulatory subunit 2 OS=Homo sapiens GN=PSMD2 PE=1 SV=3	sp|Q13200|PSMD2_HUMAN	34	49.0
26S proteasome non-ATPase regulatory subunit 3 OS=Homo sapiens GN=PSMD3 PE=1 SV=2	sp|O43242|PSMD3_HUMAN	23	53.9
26S proteasome non-ATPase regulatory subunit 4 OS=Homo sapiens GN=PSMD4 PE=2 SV=1	tr|Q5VWC4|Q5VWC4_HUMAN	11	43.1
26S proteasome non-ATPase regulatory subunit 5 OS=Homo sapiens GN=PSMD5 PE=1 SV=3	sp|Q16401|PSMD5_HUMAN	19	49.6
26S proteasome non-ATPase regulatory subunit 6 OS=Homo sapiens GN=PSMD6 PE=2 SV=1	tr|C9IZE4|C9IZE4_HUMAN	9	43.4
26S proteasome non-ATPase regulatory subunit 7 OS=Homo sapiens GN=PSMD7 PE=1 SV=2	sp|P51665|PSMD7_HUMAN	14	47.5
26S proteasome non-ATPase regulatory subunit 8 OS=Homo sapiens GN=PSMD8 PE=1 SV=2	sp|P48556|PSMD8_HUMAN	9	50.0
26S proteasome non-ATPase regulatory subunit 9 OS=Homo sapiens GN=PSMD9 PE=1 SV=3	sp|O00233|PSMD9_HUMAN	2	35.9
26S proteasome non-ATPase regulatory subunit 10 OS=Homo sapiens GN=PSMD10 PE=2 SV=1	tr|B1AJY7|B1AJY7_HUMAN	5	49.2
26S proteasome non-ATPase regulatory subunit 11 OS=Homo sapiens GN=PSMD11 PE=1 SV=3	sp|O00231|PSD11_HUMAN	27	65.2
26S proteasome non-ATPase regulatory subunit 12 OS=Homo sapiens GN=PSMD12 PE=1 SV=3	sp|O00232|PSD12_HUMAN	25	56.8
Isoform 2 of 26S proteasome non-ATPase regulatory subunit 13 OS=Homo sapiens GN=PSMD13	sp|Q9UNM6-2|PSD13_HUMAN	20	55.8
26S proteasome non-ATPase regulatory subunit 14 OS=Homo sapiens GN=PSMD14 PE=1 SV=1	sp|O00487|PSDE_HUMAN	12	42.3
Proteasome activator complex subunit 1 OS=Homo sapiens GN=PSME1 PE=2 SV=1	tr|H0YNE3|H0YNE3_HUMAN	8	41.6
Proteasome activator complex subunit 2 OS=Homo sapiens GN=PSME2 PE=1 SV=4	sp|Q9UL46|PSME2_HUMAN	3	26.4
Proteasome activator complex subunit 3 OS=Homo sapiens GN=PSME3 PE=1 SV=1	sp|P61289|PSME3_HUMAN	1	34.3
Proteasome assembly chaperone 1 OS=Homo sapiens GN=PSMG1 PE=1 SV=1	sp|O95456|PSMG1_HUMAN	2	15.3
Proteasome assembly chaperone 2 OS=Homo sapiens GN=PSMG2 PE=1 SV=1	sp|Q969U7|PSMG2_HUMAN	3	33.7
Proteasome assembly chaperone 3 OS=Homo sapiens GN=PSMG3 PE=1 SV=1	sp|Q9BT73|PSMG3_HUMAN	2	18.9
Proteasome inhibitor PI31 subunit OS=Homo sapiens GN=PSMF1 PE=2 SV=2	tr|Q5QPM7|Q5QPM7_HUMAN	5	39.9
Proteasome-associated protein ECM29 homolog OS=Homo sapiens GN=ECM29 PE=1 SV=2	sp|Q5VYK3|ECM29_HUMAN	24	27.8

Minor comments

1. In the Results section, the sentence "Since the 20S proteasome complex was not detected within nuclear lacunae (Fig. 1d), they likely originate from the sperm tail." appears to contain an error. The reference to the 20S proteasome should likely be 19S, and this should be corrected accordingly.

We have corrected this paragraph, which now reads:

'We identified a small fraction of 26S proteasomes in the 20S-PA200 sample. Since 26S proteasomes were not detected by tomography within the nucleus (Fig. 1d), they likely originate from the sperm tail. This is supported by immunofluorescence and immunohistochemistry data showing enrichment of 19S subunits in the tail of both ejaculated human sperm and mature spermatozoa in human testis tissue (Extended Data Fig. 3e-f; Extended Data Fig. 11e-j).'

2. In the Results section, the sentence "Interestingly, there was an enrichment in nuclear 20S core particles as differentiation progressed, with significantly higher levels in the nucleus of spermatocytes and spermatids, relative to spermatogonia (Fig. 3c and Extended Data Fig. 9b)." appears to cite an incorrect figure. The reference to Fig. 3c should likely be Fig. 5c, and this should be corrected accordingly.

The reviewer is correct. We have edited the referenced figure accordingly.

Reviewer #4 (Remarks to the Author):

The manuscript entitled "Molecular architecture and spatial organization of proteasomes in the human sperm nucleus" by Piotr Kolata et al. is very interesting, has solid results, and makes an impression with its illustrations, even if some of them seem to be somewhat overloaded with information. The insight into the molecular architecture and its organization with subnanometer resolution of the sperm nucleus is definitely new, and the proteasome analysis has been carried out competently. I believe that the manuscript is well suited for publication in NSMB, but I also believe that some revisions would be necessary. Below are my detailed comments and suggestions.

We would like to thank the reviewer for the supportive evaluation of our manuscript and for taking the time to provide detailed corrections.

Introduction

I numbered the lines of each page in the document, and numbered the pages to point at the exact location.
Introdcution:
Page 1, Line 40, While I do like the Fonseca 2012 reference, here Groll et al 1997 I a much better fit, that is the original ref for the 20S.

We agree with the reviewer and have corrected this reference.

Results:
Page 2, Line 55, The figure legend for 1A states 'arrows indicate regions with sperm lacunae' but seems to mean 'rectangles' and no arrowheads are visible. Please correct in the text an in the figure legend.

The reviewer is correct and we have changed the figure legend accordingly.

Page 3, line 2, not necessary to point to Fig 1 again - can be removed.

Page 3, line 8, It would be nice to see one example of double-capped PA200-20S in Fig. 1C. Also, given the low abundance - can the authors discuss the chances of these 0.3% to be true or false positives? Again, one or two examples in 1C would be enough.

We have now added two examples of double-capped particles. to Fig.1c (also added below).

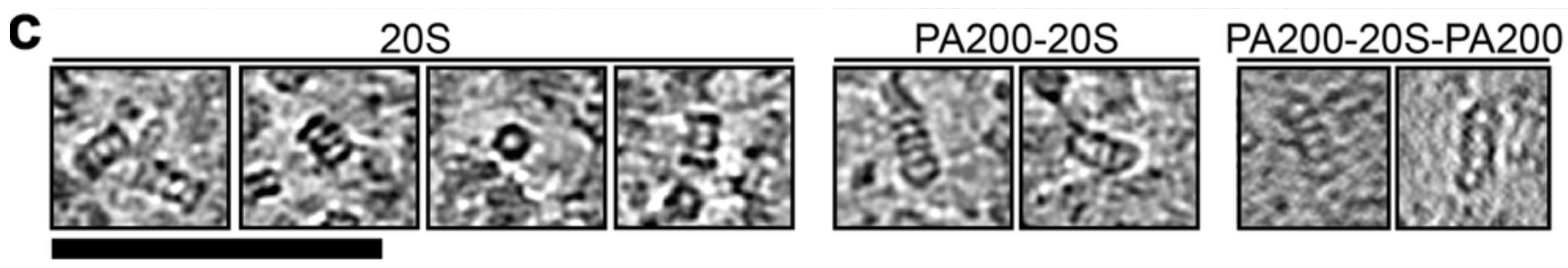

Minor comment: Fig.1d: subunit coloring is spilling over to neighbouring subunits (e.g. the orange for a4s)

We have attempted to improve colouring for our subtomogram average maps. However, upon closer inspection, the “spilling over” effect occurs due to the limited resolution. Particularly, our maps at 7-14 Å resolution have poor separation of non-helical features such as extended loops and beta-strands, which often form contacts between subunits, leading to inaccuracies in colouring. As we use single-particle maps for structure analysis, we believe that this minor inaccuracy will not confuse the reader.

Page 3, line 12, Fig. 2b: the coloring of the lacunae is hardly visible in the lower panels. The upper panels are hard to navigate. The nucleus is only recognizable after spotting the colored dots in the lower panel. Needs better markings, better presentation.

We have now improved the colouring of the lacunae in Fig.2b. For clarity, we have also drawn dashed lines around each sperm nucleus in Fig.2b for easier recognition.

AND: the video2 uses different colors for lacunae than in the figure, why?

We have modified Fig.2b so that the colour of lacunae is consistent with the one used in the video.

Page 4, line 6-7, Needs to be rephrased. “Micrographs allowed us” sounds odd.

We rephrased this sentence to:

‘To investigate the structure of sperm-specific proteasomes, we isolated native proteasomes from human sperm and performed cryo-EM analyses (Extended Data Fig. 4a-e).’

Fig.3: some panels are too densely packed, their markings are overlapping into other panels. Figure needs more space or should be better arranged to avoid overlapping. I would suggest to make Fig a, b, c, f, g and h page width large and place Fig 3 d and I somehow below.

We rearranged and improved this figure for clarity.

Page 4, line 12 - 15, great finding great statement.
Page 4, line 30, Extended data Fig 7c, Labelling of alpha4-R4 in lower panel is inconclusive.

We have modified the figure to highlight the arginine.

Page 4, line 33, Extended data Fig 7d-f, Water molecule color should be black, not green.

We changed the water molecule colour to dark grey.

Page 4, line 34, Fig. 3f: no difference discernable because image is very small.

In the new version of Fig. 3, panel 3f has been removed. Instead, Extended Data Fig. 8b features the same panel with more extensive annotation highlighting the conformational changes in the alpha subunits that occur upon PA200 binding.

Page 4, line 38, Fig. 3h: Using orange and dark-orange is not different enough.

We modified panel 3h (panel 3g in the new version of this figure) for clarity. We changed the colours to yellow and dark orange and highlighted the main differences between the two structures to improve clarity.

Page 4, line 41, Fig. 3i: Why is “PA200” spelled with two different colors?

This is to highlight that PA200 is shown as an electrostatic surface

Page 4, line 45-53:
This is a questionable 26S structure, even for a AAA-ATPase-only 19S. To be convinced I would need to see the fitted pdb of the AAA-Atpase aligning well with some helices of the density. This should be possible at 3.2A.

Due to flexibility? I think the 19S is rigid enough. It is more likely that a) the density is a mixture of PA200-20S and 19S-20S or b) the 19S is not fully assembled and only carries the AAA-ATPase. Also problematic: it seems like the upper AAA-ATPase ring is almost not present, it seems to be less well occupied (which is not possible, it is the N-terminal part).
The authors should show a fit of the AAA-Atpase-ring in the proposed 26S map. This model does not look completely reliable.
Update: ext. Fig. 8e and 8f show a bit more of the AAA-Atpase density and their pdb fit seems to correlate well. This suggests, they have also 20S with AAA-Atpase - but I wouldn't call it "26S".

We thank the reviewer for the critical insight into our 26S structure. Indeed, the poor definition of other 19S subunits in the map was unexpected. However, the fit of the PDB ID: 5L4G structure into our density was sufficient to interpret this map as corresponding to at least a fragment of the 26S proteasome (Extended Fig. 8f).
As the reviewer noted, this may appear an overinterpretation of the cryo-EM data. As a result, we have now renamed this map to 20S-AAA ATPase in Extended Fig 8e, as requested (shown below). We have also included a model-map cross-correlation value in the figure legend, which is especially high for unrefined coordinates, showing that the fit is reliable. We have also improved the presentation of Extended Fig. 8 and added panels h-i (shown below).
We investigated the source of the poor definition of the other 19S subunits in our cryo-EM map. One possibility was that the box size used prevented reconstruction of the entire particle. Therefore, we re-processed these data with an extended box size (see Extended Data Fig 8h and 9 and Materials & Methods). Initially, we obtained a similar 20S-AAA ATPase map, but further classification revealed multiple classes with low-resolution ‘19S-like’ densities extending out of the AAA-ATPase region. One of the classes allowed confident fitting of the entire 26S proteasome (PDB ID: 6MSB) (Extended Data Fig. 8i). Other classes showed similar features, but with different conformations and/or with some elements missing. Unfortunately, our small number of ‘full’ 19S particles (1,842) was not sufficiently homogeneous to define high resolution features.

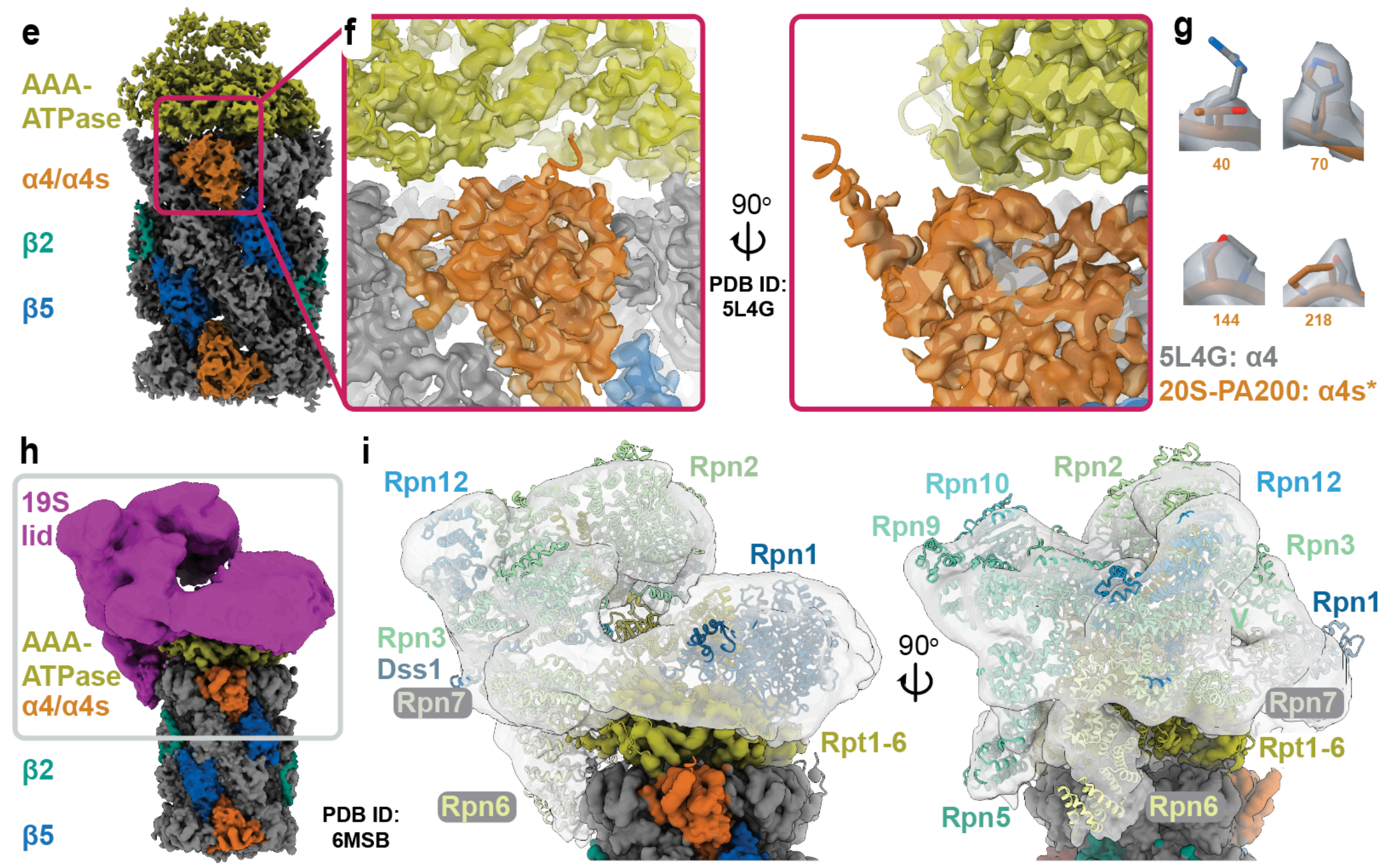

Extended Fig.8 e-i - **e,** cryo-EM map of the 20S AAA-ATPase complex isolated from human sperm. The AAA-ATPase (Rpt1-6, green), α4/α4s* (orange), β2 (cyan) and β5 (blue) subunits are indicated. **f,** the interface between AAA-ATPase subunits and α4/α4s*. The 20S-AAA ATPase proteasome structure (PDB ID: 5L4G) was rigid-body fitted in the map (model-map cross-correlation = 0.7759) and shown as cartoons. **g,** side-chain densities in the sperm cryo-EM 26S proteasome map with modelled α4 (grey, PDB ID: 5L4G) and α4s* (orange, 20S-PA200 coordinates) side chains. **h,** cryo-EM reconstruction of the 26S proteasome isolated from human sperm. The segmented, local-resolution filtered volume is shown. The same subunits as in **e,** as well as the remainder of the 19S activator (purple), are indicated. **i,** the fit of the entire 26S proteasome structure (PDB ID: 6MSB, shown as cartoons) in the sperm 26S map. The 20S-AAA ATPase density is opaque and coloured as in (**h**), while that corresponding to the remainder of the 19S activator is in transparent grey. The 19S subunits are indicated. Model-map cross-correlation = 0.622. In all panels, amino acids are marked using single-letter amino acid code.

Page 5, line 5, Fig. 4a-c: Isn't the rotation from b to c 180 deg and not 90 as indicated in the figure?

The rotation of 90 degrees is correct in this specific case

Fig. legend
b) "rearrangement required" should read "rearrangement is required"
d) "the structure of β2 subunit" should read "the structure of the β2 subunit"
d) "In the left panel, the same view as in b is
used." d seems to be identical to c, not b

We have corrected these errors and typos.

Page 6, line 9-10, PRMC1 is used to test for 19S localization, however, this is a AAA-Atpase subunit and does not clarify whether a fully assembled 19S or only an AAA-Atpase is present. In order to claim "19S" without further explanation I suggest to use at least one other subunit as marker (Rpn10, Rpn1, Rpn11…)

We performed immunocytochemistry against Rpn1 (PSMD2) in human sperm cells followed by SIM. The localisation of PSMD2 was consistent with our earlier results for PSMC1, with signal detected in the sperm tail but not in the nucleus (shown below). We have added these data to the revised manuscript (Extended Data Fig. 3f).

Interestingly, we also found a published proteomics dataset (PMID: 31244561) from human sperm cells where many of the 19S subunits were detected, including PSMC1 and PSMD2 (Table1 below, showing data extracted from PMID: 31244561). This, together with our data, strongly points to the possibility of fully assembled 19S particles in sperm cells.

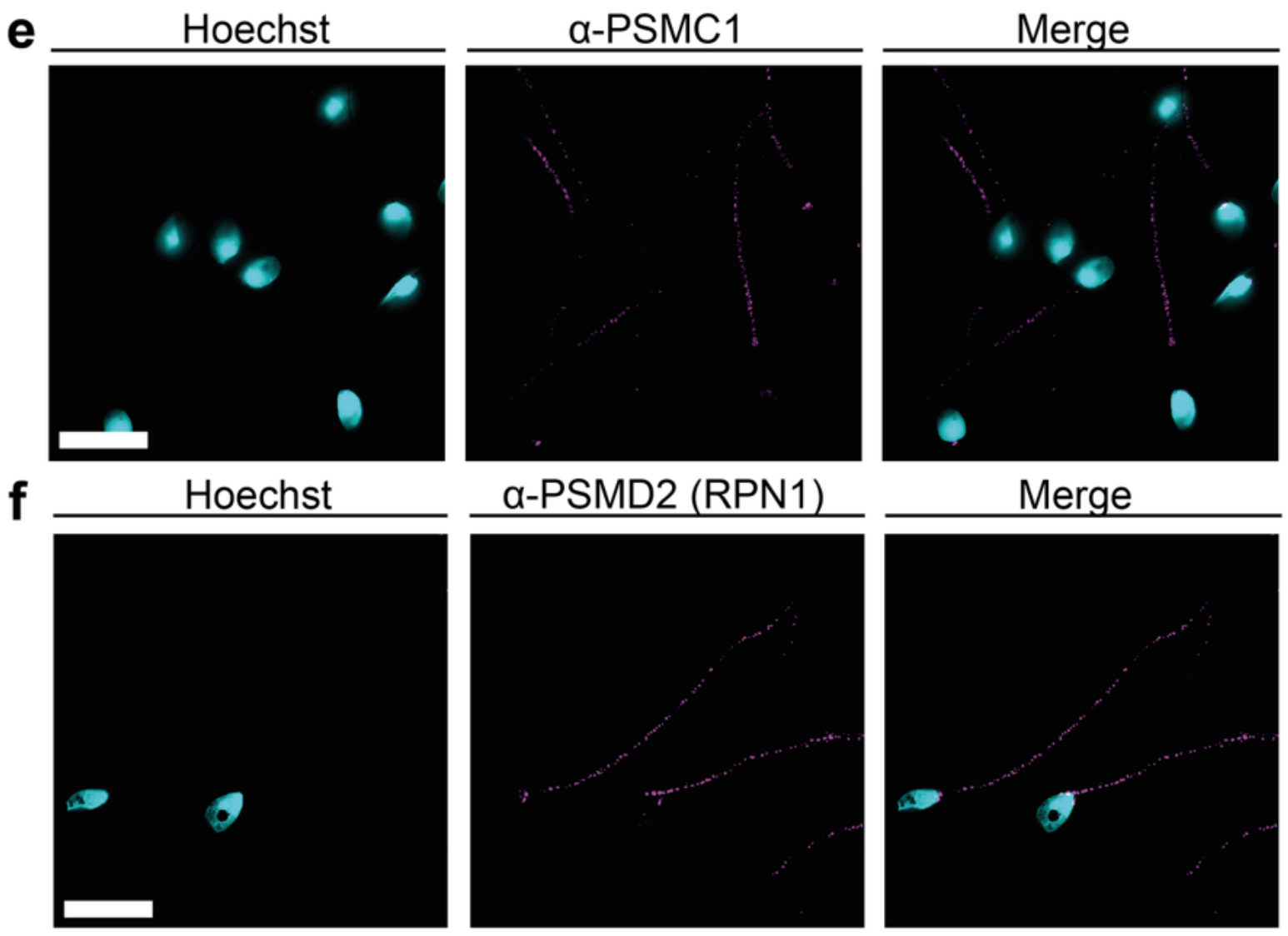

We have also added the following description to our results section

'Interestingly, we did not detect nuclear signal for the 19S regulatory particle in ejaculated sperm, using the AAA-ATPase PSMC1 and PSMD2 (Rpn1) as markers. Instead, both proteins were enriched in the tail region (Extended Data Fig. 3e and f). In mouse sperm, PSMC1 signal was substantially weaker overall and was also absent from the nucleus (Extended Data Fig. 3c).'

and to our discussion section:

'Interestingly, in human testis tissue, the 19S AAA-ATPase subunit PSMC1 was enriched in the tail of differentiating spermatids and tails of mature spermatozoa (Extended Data Fig. 11f-j). Furthermore, both PSMC1 and PSMD2 (Rpn1), another 19S subunit, localised exclusively to the tail of ejaculated sperm cells (Extended Data Fig. 3e-f).'

Table 1 - *Extracted from Additional file 1: Table S1 of PMID:31244561*. Proteasome-related hits from total proteins idenfied in human sperm by proteomics.

Name and isoform (if applicable)	Accession	Peptides	%coverage
Proteasome subunit alpha type-1 OS=Homo sapiens GN=PSMA1 PE=1 SV=1	sp|P25786|PSA1_HUMAN	23	72.2
Proteasome subunit alpha type-2 OS=Homo sapiens GN=PSMA2 PE=1 SV=2	sp|P25787|PSA2_HUMAN	22	64.5
Proteasome subunit alpha type-3 OS=Homo sapiens GN=PSMA3 PE=1 SV=2	sp|P25788|PSA3_HUMAN	21	58.0
Proteasome subunit alpha type-4 OS=Homo sapiens GN=PSMA4 PE=1 SV=1	sp|P25789|PSA4_HUMAN	18	51.0
Proteasome subunit alpha type-5 OS=Homo sapiens GN=PSMA5 PE=1 SV=3	sp|P28066|PSA5_HUMAN	25	69.3
Proteasome subunit alpha type-7 OS=Homo sapiens GN=PSMA7 PE=1 SV=1	sp|O14818|PSA7_HUMAN	17	62.9
Isoform 3 of Proteasome subunit alpha type-7-like OS=Homo sapiens GN=PSMA8	sp|Q8TAA3-5|PSA7L_HUMAN	18	72
Proteasome subunit beta type-1 OS=Homo sapiens GN=PSMB1 PE=1 SV=2	sp|P20618|PSB1_HUMAN	20	59.3
Proteasome subunit beta type-2 OS=Homo sapiens GN=PSMB2 PE=1 SV=1	sp|P49721|PSB2_HUMAN	8	47.3
Proteasome subunit beta type-3 OS=Homo sapiens GN=PSMB3 PE=1 SV=2	sp|P49720|PSB3_HUMAN	14	42.4
Proteasome subunit beta type-4 OS=Homo sapiens GN=PSMB4 PE=1 SV=4	sp|P28070|PSB4_HUMAN	21	57.2
Proteasome subunit beta type-5 OS=Homo sapiens GN=PSMB5 PE=1 SV=3	sp|P28074|PSB5_HUMAN	17	58.9
Proteasome subunit beta type-6 OS=Homo sapiens GN=PSMB6 PE=1 SV=4	sp|P28072|PSB6_HUMAN	10	46.0
Proteasome subunit beta type-7 OS=Homo sapiens GN=PSMB7 PE=1 SV=1	sp|Q99436|PSB7_HUMAN	12	55.6
Proteasome subunit beta type OS=Homo sapiens GN=PSMB9 PE=2 SV=1	tr|A2ACR1|A2ACR1_HUMAN	1	26.5
Proteasome subunit beta type-10 OS=Homo sapiens GN=PSMB10 PE=1 SV=1	sp|P40306|PSB10_HUMAN	2	20.9
Proteasome activator complex subunit 4 OS=Homo sapiens GN=PSME4 (PA200) PE=1 SV=2	sp|Q14997|PSME4_HUMAN	35	27.4
26S proteasome non-ATPase regulatory subunit 1 OS=Homo sapiens GN=PSMD1 PE=1 SV=2	sp|Q99460|PSMD1_HUMAN	37	48.7
26S proteasome non-ATPase regulatory subunit 2 OS=Homo sapiens GN=PSMD2 PE=1 SV=3	sp|Q13200|PSMD2_HUMAN	34	49.0
26S proteasome non-ATPase regulatory subunit 3 OS=Homo sapiens GN=PSMD3 PE=1 SV=2	sp|O43242|PSMD3_HUMAN	23	53.9
26S proteasome non-ATPase regulatory subunit 4 OS=Homo sapiens GN=PSMD4 PE=2 SV=1	tr|Q5VWC4|Q5VWC4_HUMAN	11	43.1
26S proteasome non-ATPase regulatory subunit 5 OS=Homo sapiens GN=PSMD5 PE=1 SV=3	sp|Q16401|PSMD5_HUMAN	19	49.6
26S proteasome non-ATPase regulatory subunit 6 OS=Homo sapiens GN=PSMD6 PE=2 SV=1	tr|C9IZE4|C9IZE4_HUMAN	9	43.4
26S proteasome non-ATPase regulatory subunit 7 OS=Homo sapiens GN=PSMD7 PE=1 SV=2	sp|P51665|PSMD7_HUMAN	14	47.5
26S proteasome non-ATPase regulatory subunit 8 OS=Homo sapiens GN=PSMD8 PE=1 SV=2	sp|P48556|PSMD8_HUMAN	9	50.0
26S proteasome non-ATPase regulatory subunit 9 OS=Homo sapiens GN=PSMD9 PE=1 SV=3	sp|O00233|PSMD9_HUMAN	2	35.9
26S proteasome non-ATPase regulatory subunit 10 OS=Homo sapiens GN=PSMD10 PE=2 SV=1	tr|B1AJY7|B1AJY7_HUMAN	5	49.2
26S proteasome non-ATPase regulatory subunit 11 OS=Homo sapiens GN=PSMD11 PE=1 SV=3	sp|O00231|PSD11_HUMAN	27	65.2
26S proteasome non-ATPase regulatory subunit 12 OS=Homo sapiens GN=PSMD12 PE=1 SV=3	sp|O00232|PSD12_HUMAN	25	56.8
Isoform 2 of 26S proteasome non-ATPase regulatory subunit 13 OS=Homo sapiens GN=PSMD13	sp|Q9UNM6-2|PSD13_HUMAN	20	55.8
26S proteasome non-ATPase regulatory subunit 14 OS=Homo sapiens GN=PSMD14 PE=1 SV=1	sp|O00487|PSDE_HUMAN	12	42.3
Proteasome activator complex subunit 1 OS=Homo sapiens GN=PSME1 PE=2 SV=1	tr|H0YNE3|H0YNE3_HUMAN	8	41.6
Proteasome activator complex subunit 2 OS=Homo sapiens GN=PSME2 PE=1 SV=4	sp|Q9UL46|PSME2_HUMAN	3	26.4
Proteasome activator complex subunit 3 OS=Homo sapiens GN=PSME3 PE=1 SV=1	sp|P61289|PSME3_HUMAN	1	34.3
Proteasome assembly chaperone 1 OS=Homo sapiens GN=PSMG1 PE=1 SV=1	sp|O95456|PSMG1_HUMAN	2	15.3
Proteasome assembly chaperone 2 OS=Homo sapiens GN=PSMG2 PE=1 SV=1	sp|Q969U7|PSMG2_HUMAN	3	33.7
Proteasome assembly chaperone 3 OS=Homo sapiens GN=PSMG3 PE=1 SV=1	sp|Q9BT73|PSMG3_HUMAN	2	18.9
Proteasome inhibitor PI31 subunit OS=Homo sapiens GN=PSMF1 PE=2 SV=2	tr|Q5QPM7|Q5QPM7_HUMAN	5	39.9
Proteasome-associated protein ECM29 homolog OS=Homo sapiens GN=ECM29 PE=1 SV=2	sp|Q5VYK3|ECM29_HUMAN	24	27.8

Page 7, line 7-10: Can you elaborate on the implications?

We have expanded this section, which now reads:

'Selective enrichment of 19S or 26S proteasomes at these regions would provide a distinct proteostatic environment relative to the nucleus of differentiating cells. Contrastingly, in mice, we detected lower signal for 19S in the tail of mature spermatozoa (Extended Data Fig. 3c), and we could not observe co-localisation with acetylated tubulin in differentiating spermatids/spermatozoa (Extended Data Fig. 4c, Extended Data

Fig. 12d). This further highlights differences between organisms and suggests that requirements for paternal proteasomes may differ between species post-fertilisation.'

Page 7, line 55, Why is the tripeptide structure so well resolved? Shouldn't the density wash out in case different tripeptides are being processed? Why do all proteasomes show the same tripeptide? If it is a trapped intermediate then it should be explained why this particular tripeptide is so likely to be the trap.

We believe that the tripeptide is in fact, a mixture of different amino acids, as the side chains, except for the arginine, are poorly resolved. We tentatively modelled the peptide as Val-Val-Arg, to indicate that the side chain densities are more bulky amino acids than alanines, but this is speculative, as we highlight in the manuscript. We suggest that protamines (Arginine-rich proteins) might be possible degradation targets of sperm proteasomes, and that multiple Arg-containing protamine tripeptides might occupy the active site, resulting in strong polypeptide backbone density and smeared side-chain regions, as observed in our structure. We have edited the discussion to better clarify these points:

'Remarkably, our high-resolution 20S structure shows a plausible Arg-containing tripeptide covalently linked to the catalytic threonine of β2 subunits, a feature not previously reported to our knowledge (Fig. 4). There are several possible reasons for this. First, our gentler purification method, compared with previous studies (Harshbarger et al., 2015; Schrader J., 2016), preserves proteasomes in their active state throughout the process and may also supply substrate up to the cryo-EM sample preparation step (Extended Data Fig. 4 a-e). In particular, nuclease treatment at the beginning of the purification, combined with a reducing environment throughout the process, likely released monomeric protamines. As small peptides, these could enter the 20S CP for degradation, and remain visible in the active site due to low activity of the 20S CP (Coux, 1996). While the sperm 20S gate in our structure adopts a closed conformation, various studies indicate that it can be opened by oxidised and unfolded proteins with exposed hydrophobic patches (Grune, 2003 ; Jung et al., 2014; Latham et al., 2014; Orlowski, 2003) or by peptides containing the C-terminal HbYX (hydrophobic-tyrosine-any amino acid) motif (Opoku-Nsiah et al., 2022; Smith, 2007). Indeed, although protamines are highly positively charged proteins, they do include a version of the XbYX motif in their sequence (residues 15-18, Tyr-Tyr-Arg; UniProt ID: P04553). During proteolysis by the β2 subunit, the protamine degradation product containing the Tyr-Tyr-Arg motif at the C-terminus could be formed and released, opening the proteasomal gate. Similarly, an early study suggested that polycationic substances, including protamines, enhance proteasomal activity (Mellgren, 1990), and more recent data propose proteasomal activation by polyamines (Kudriaeva et al., 2020). This supports the idea that protamines could be degraded by the sperm 20S CP. We hypothesise that the active site of the β2 subunit in our map might be occupied by one of the protamine fragments, e.g. residues 6-8, Cys-Cys-Arg motif or other Arg-containing motifs. However, further work will be required to validate this hypothesis and determine the biochemical identity of the peptide.'

Page 8, line 17, '19S was enriched' - I think it is AAA-ATPase not 19S, see also comments above. Page 8, line 21, 'our map' is missing all other subunits, including Rpn1 which is close to alpha4.

We rephrased this section to take this suggestion into account. It now reads:

'Interestingly, in human testes, the 19S AAA-ATPase subunit PSMC1 was enriched in the tail of differentiating spermatids (Extended Data Fig. 11f-j). Furthermore, both PSMC1 and PSMD2 (Rpn1), another 19S subunit, localised exclusively to the tail of mature spermatozoa, both in testis tissue and in ejaculated sperm (Extended Data Fig. 3e-f). This enrichment was not shared by 20S subunits (Extended Data Fig. 10a-d). Several studies have proposed that α4s confers sperm 20S preference for 19S (Wang et al., 2025; Zivkovic et al., 2022). However, our structural data does not support this notion, as there are no interactions between α4/α4s and AAA-ATPase subunits of 19S in our map (Extended Data Fig. 8e-g).'*

Page 9, line 37-40: It is okay to cite its own papers, however, the original protocols are derived from others. One may refer to the original publications for lamella milling. I think the authors know what I am aiming for.

We agree with the reviewer, and we now cite two early publications on the method: PMID: 22392984, PMID: 24090931.

Page 10, line 2, correct 'weigthed' to weighted.
Page 10, line 5, delete 'and', 'extensive'.

These typos have been corrected.

Page 10, line 9- : I think the information: 'to obtain a 20S map at 6.8 Å resolution; a 20S-PA200 map at 9.5 Å resolution; and a PA200-20S-PA200 map at 14.4 Å resolution.' Can be deleted out of the methods section.

This has now been deleted from the methods section.

Page 11, line 42: exchange picking with picks and remove change to positive not positives.

We have corrected the text.

Reviewers' Comments:

Reviewer #3:
Remarks to the Author:
The authors have addressed the reviewers' comments comprehensively and thoughtfully, and I believe that the manuscript has reached the standard required for publication. The additional experiments, analyses, and revisions have substantially strengthened the study, and I support acceptance. I have only one minor suggestion that may further strengthen the interpretation of the α4s* findings.

The authors made a reasonable effort to assess α4s* expression using RNA extracted from mature spermatozoa; however, the results were understandably inconclusive given the transcriptionally inactive state of mature sperm cells. Since the α4s* protein incorporated into sperm proteasomes must be synthesized during spermatogenesis, expression of the corresponding transcript would be expected at earlier developmental stages in the male germline.

If suitable material or publicly available transcriptomic datasets are readily available (e.g., testis tissue, spermatogenic cells, or published human/mouse RNA-seq datasets), additional evidence supporting α4s* transcript expression during spermatogenesis would further strengthen the interpretation. Alternatively, if transcript-level validation is not feasible, I encourage the authors to more explicitly discuss the structural basis for distinguishing α4s* from α4s. In particular, it would be helpful to clarify whether the local map quality and resolution in the isoform-discriminating regions are sufficient to support the assignment with high confidence, and to acknowledge any limitations if appropriate.

I do not consider this point essential for publication, as the current structural and proteomic evidence already provides substantial support for the authors' conclusions. Nevertheless, additional clarification along either of these lines would further strengthen the manuscript and help readers assess the robustness of the α4s* assignment.

We would like to thank the reviewer for highlighting our efforts during the revisions and for supporting the acceptance of our work. We looked at publicly available testis RNA-Seq datasets in the Genotype-Tissue Expression (GTEx v8) project (DOI: 10.1126/science.aaz177) and found that the α4s* splice variant is the predominant isoform of α4s transcribed during spermatogenesis, providing direct support to our structural findings. We now cite these data in our discussion (page 8, lines 1-2), and shown below:

'Consistent with our structural observations, this splice variant is the predominant α4s isoform transcribed during spermatogenesis[50], and a published proteomics dataset from human sperm cells identified unique peptides for this variant and not for α4s[51]'

We have also added Extended Data Fig. 5b-c to better show the local map quality and resolution in the isoform-discriminating region. We describe these edits on page 4, lines 26-28, and reproduced below:

'The density region adjacent to the missing loop is exceptionally well resolved, and no residual signal corresponding to the loop is visible, corroborating that the region is compositionally homogeneous.'
