# Supplementary material for: Molecular architecture and spatial organization of proteasomes in the human sperm nucleus
Source: Nat Struct Mol Biol. 2026 Sep 1;33(9):1367–80. doi: 10.1038/s41594-026-01870-z (PMC13561864; doi:10.1038/s41594-026-01870-z)

Source Data – Extended Data Fig. 4c

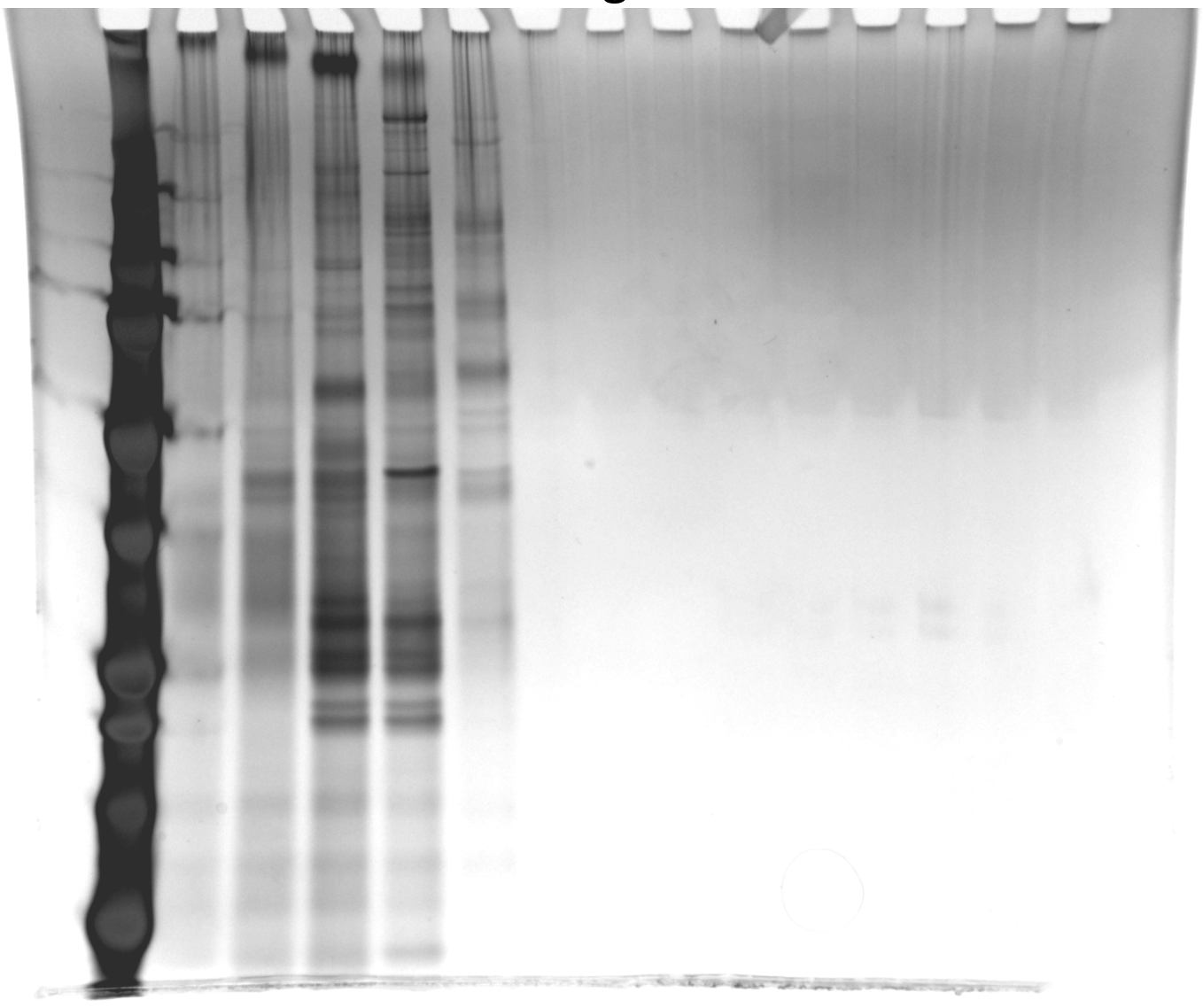

Source Data – Extended Data Fig. 4d

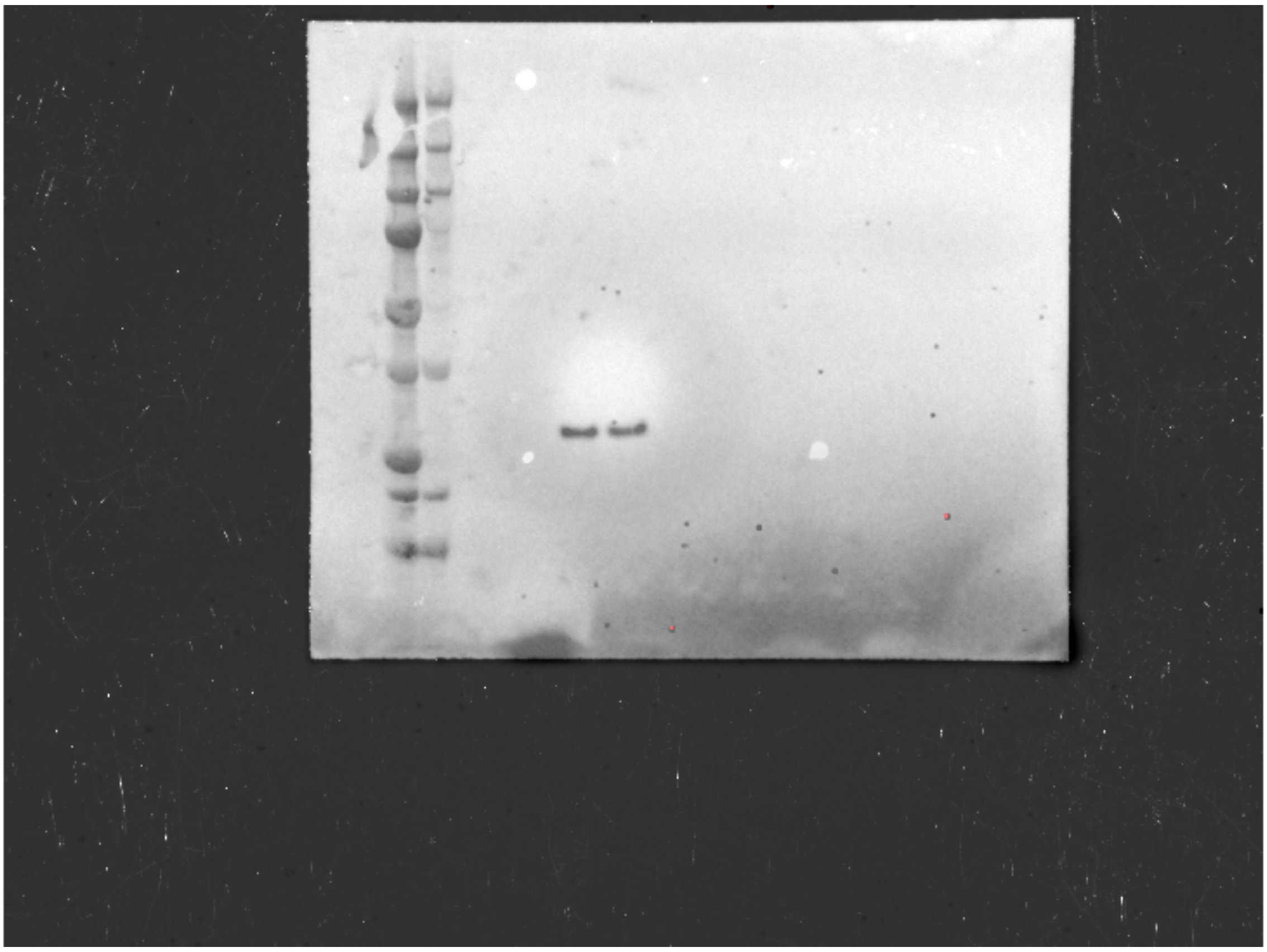

Supplement: Supplementary file 3 [file 41594_2026_1870_MOESM8_ESM.pdf]
